# Supplementary material for: Multilevel design and construction in nanomembrane rolling for three-dimensional angle-sensitive photodetection
Source: Nat Commun. 2024 Apr 9;15:3066. doi: 10.1038/s41467-024-47405-2 (PMC11004118; doi:10.1038/s41467-024-47405-2)
Supplement: Supplementary file 1 — Supplementary Information [file 41467_2024_47405_MOESM1_ESM.pdf]

Supplementary Information for

Multilevel design and construction in nanomembrane rolling for three-dimensional angle-sensitive photodetection

Ziyu Zhang, Binmin Wu, Yang Wang, Tianjun Cai, Mingze Ma, Chunyu You, Chang Liu, Guobang Jiang, Yuhang Hu, Xing Li, Xiangzhong Chen, Enming Song, Jizhai Cui, Gaoshan Huang, Suwit Kiravittaya and Yongfeng Mei

Email: yfm@fudan.edu.cn

List of Contents

Supplementary Notes 3
Supplementary Figures 51
Supplementary Tables 110
Supplementary References 112

Supplementary Notes

Supplementary Note 1. Reaction-diffusion model for H_2O_2/Ge system

FEM simulation was realized via COMSOL. For ultrathin nanomembrane materials, the boundary movement hardly changes with the thickness, so we use a two-dimensional model to reduce the calculation amount while ensuring the accuracy of the result. The model is calculated by coupling the mass transfer and the laminar flow field to simulate the diffusion and reaction process of the etchant to the interface of the sacrificial layer. The diffusion is determined by Fick's law, and the laminar flow field is determined by the Stokes equation, continuity equation, convective diffusion equation and chemical reaction equation[4-6]. For the etching of the sacrificial layer by the etchant, the amount of mass transfer in the normal direction of the interface is converted into the corresponding normal moving speed of the mesh. The movement of the boundary is realized by the moving grid module. To verify the relationship between etching direction, vertex of sacrificial layer is selected to track the movement during etching process. (Supplementary Figures 1**a, b**). Affect by the asymmetric in the fixed end and opposite edge of sacrificial layer, the velocity ratio is increased with the decrease of W/L. The large velocity ratio in small W/L indicate the anisotropic etching and change in rolling direction, result in the formation of pre-strained nanomembranes.

After obtaining the calculation results of the sacrificial layer moving boundary with time, each node on the boundary at multiple discrete time points will be exported as coordinate parameters and converted into the geometric features of the subsequent segmentation model (Figure 2**a**). Diffusion and chemical reaction involve many environmental conditions, reflecting in some key physical parameters. We explored parameters that mainly effect the reaction-diffusion process[1]. Here, we will discuss the applicability of model which simulate the boundary movement of the sacrificial layer in etching process, and the feasibility to the H_2O_2/Ge etching model. In this model, etching time is normalized to compare the morphology in whole process. To analysis the morphology changes of sacrificial layer in different condition, we compare them in the

change of whole boundary and displacement in feature points AA' in normalized time. we take 5/10/20/40/80×40 μm^2 pattern as example to present the boundary change, and show displacement of points in various widths. In following experiment, we assume T = 293 K, D = 10^{-9} m^2 s^{-1}, $k_{273K} \approx 10^{-6}$, c = 10 mol L^{-1}, and no flow field during etching if the condition is not specially mentioned[2].

(1) Rate control steps

Etching rate of the reaction-diffusion model can be expressed as:

$$\frac{1}{v} = \frac{1}{kC} + \frac{1}{D\Delta C/\delta} \quad (1)$$

where is k reaction rate coefficient, C is concentration of etchant, D is diffusion coefficient, and δ is diffusion length. The total etching rate depend on the slow one more. That is, when the diffusion step is very slow, the etching rate approximately equal to the corresponding mass transfer rate of diffusion step, which is the same for chemical reaction step. From Supplementary Figure 2**a** we can see that with the decrease of k, the movement of boundary depend more on the geometric morphology, which is also the transition of rate control from diffusion-control process to reaction-control process. When k is small enough, the boundary will completely depend on the geometric morphology, which is mismatch with the experimental result, indicating the model are practicable to diffusion-control process reaction.

(2) Temperature

The change of temperature could have drastic effects on the diffusion coefficient and reaction rate coefficient, leading to changes in reaction-diffusion process. In the reaction of Ge/H_2O_2, diffusion activation energy $Q \approx$ 4-20 kJ mol^{-1}, reaction activation energy $E_a \approx$ 46.035 kJ mol^{-1}, we set T from 273 K to 373 K to research the influence. However, changes of boundary movements from 273 K to 373 K are indistinct. In Ge/H_2O_2 model,

change in temperature (273-373 K) are unable to affect the reaction control factor. However, if the temperature difference is large enough or the ΔQ and ΔE_{a} is large enough, the transition from diffusion-controlled reaction to chemical dynamic-controlled reaction is very possible.

(3) Etchant concentration

From the equation of chemical reaction rate, we know that reaction rate directly depends on the concentration. We compared boundary movement of various etchant concentration from to, finding that etchant with an extreme high concentration will affect the boundary movement, but it is acceptable in experiment concentration. We also emphasized that the default parameters are under diffusion control, which fit with our experimental condition than the chemical reaction control.

(4) Flow field

If the velocity field of etchant is considered, the Fick's model will be changed into:

$$\frac{\partial C}{\partial t} + \mathbf{u} \cdot \nabla C = D \nabla^2 C \quad (2)$$

And the flow field in the etching process can be defined as[3]:

$$\rho \frac{\partial \mathbf{u}}{\partial t} + \rho(\mathbf{u} \cdot \nabla)\mathbf{u} = \nabla \cdot [-p\mathbf{I} + \mu(\nabla \mathbf{u} + (\nabla \mathbf{u})^T)] \quad (3)$$

we consider a velocity field in y direction with a constant velocity $\mathbf{u_y}$ to observe the effect on the morphology. When the velocity field is larger than the 10^{-4} m s^{-1}, morphology feature will obviously change. It is worth noting that bubble generated by heating will inevitably causing the flow in fluid under high temperature, which will make the model unpredictable. Thus, boundary movement model is not applicable to the etching model containing bubble and high-speed turbulence.

(5) Thickness of sacrificial layer

During the etching process, etching rate will be different in the different position of sacrificial layer in thickness direction due to the spatial asymmetry in real. Here we take 3D model to explore the morphology feature and the displacement feature point displacement to judge the rationality of 2D model. The top layer of sacrificial layer is exposed in etchant, while the bottom layer is bonded to substrate, causing the different diffusion process. For a thickness much smaller than width and length, the morphology of top layer and bottom layer is almost the same, which is able to be simplified into 2D model.

(6) Insoluble product generated on surface

In some solid-liquid chemical reaction, insoluble product will be generated on the solid surface to hinder the etching process. we estimate an ideal model with a series of k change over time, where

$$k = 10^{-\alpha t} k_{\mathrm{start}}\,, (0 \le t \le 1) \quad (k_{\mathrm{start}} = 10^{-2}) \tag{4}$$

$$k_{\mathrm{end}} = 10^{-\alpha} k_{\mathrm{start}}\,, (t = 1) \quad \Delta k = 10^{-\alpha} \tag{5}$$

When Δk is smaller, the etching is hindered more by product. Herein, a new boundary movement model for insoluble product should be considered in sequential studies.

(7) Type of material and etchant

For soluble solid-liquid chemical reaction, the different material and etchant can be attributed to different D and k. Under diffusion control condition, we simulated boundary movement with D from 10^{-14} to 10^{-9} m^2 s^{-1}, in which the most solid-liquid reactions occur. (For most diffusion in liquid, $D \sim 10^{-10}$ to 10^{-9} m^2 s^{-2}.) Under diffusion control and liquid

condition, diffusion coefficient hardly effect the boundary movement.

In general, Ge/H_2O_2 etching is a solid-liquid chemical reaction without dense surface products, which is also diffusion-controlled reaction. Besides, there is no fast flow field or turbulence, because solution is not boiling in 70 ℃, and it will not generate gas severely. At last, the thickness of nanomembranes is much smaller than length and width. Herein, the Ge/H_2O_2 system is well adapted for reaction-diffusion model.

Supplementary Note 2. Model selection for sacrificial layer

To simulate chemical wet etching happened in Ge sacrificial layer with high accuracy, a proper mass transfer and reaction model is necessary. As shown in Supplementary Figure 5, we arranged the two-phase mass transfer system contain solid phase to discuss the availability to sacrificial layer. Classified by the phase of another reaction, we sorted three kinds of chemical reaction: solid-solid reaction, liquid-solid reaction, and gaseous-solid reaction. For solid-solid reaction, parabola velocity formula is mostly used to express granular solid reaction under molten state[4]. However, there is no diffusion exist in solid matter, which is inapplicable to etchant. As for liquid-solid and gaseous-liquid reaction, they both meet the feature of diffusion and reaction, and the essential difference between them is only density, diffusion coefficient, and some hydrodynamic parameters[5,6]. Considering reaction type, we keep diving reaction model into physical reaction and chemical reaction. Mass transfer in dissolution model only concentrate on the dissolution rate of reactant[7], which is incomplete for Ge/H_2O_2 system that contain chemical reaction rate. With the addition of chemical reaction equation, a group of equations that complete reflect chemical wet etching has been established. The balance reached when the mass transferred by diffusion equal to the mass consumed by chemical reactions. Moreover, the time dependency of etchant concentration also affects the simulation of model, so we set two group of equations —— unsteady diffusion reaction model and steady diffusion model. For unsteady reaction model, difference method is a common method to calculate the change in etchant concentration. In FEM modeling software, we replace the method with designated concentration on boundary, where the concentration data comes from experiment of H_2O_2 thermal decompose rate.

Supplementary Note 3. FEM modeling for unidirectional rolling

We set up two types of FEM models to release behavior of nanomembrane with equidistant movement and etching process movement, respectively. As shown in Supplementary Figure 6., when the boundary movement of the sacrificial layer over time is not considered in conventional equidistant movement FEM, the Si/Cr bilayer will eventually roll into a tubular structure regardless of width, which is obviously different from the experimental results.

Supplementary Note 4. FEM modeling for different patterns

When the geometry of the nanomembrane changes, its boundary conditions will affect the concentration distribution of the etchant, but the effect on the flow velocity and concentration will be minimal, because the concentration distribution of the etchant in the hydrostatic state mainly depends on its diffusion coefficient and concentration layer. Moreover, the direction of the etchant flux will always follow the change of the concentration gradient, which also reflects the broad applicability of multilevel FEM.

First, we studied the concentration distribution and etchant flow direction of different forms of sacrificial layers. As shown in the Supplementary Figure 7, the concentration gradient and etchant flow direction during etching of triangles, semicircles, parallelogram strips and rectangles are all perpendicular to the sacrificial layer boundary, and the etchant concentration decreases as the distance between the sacrificial layer interface decreases. It shows that mass transfer mainly occurs at the solid-liquid interface.

Subsequently, we analyzed the etchant concentration at the solid-liquid interface, as shown in the Supplementary Figures 8, 9. It can be seen that for various sacrificial layer patterns and different sizes, the concentration of the etchant at the solid-liquid interface is extremely low compared with the concentration of the etchant at the initial condition (10 mol L^{-1}) (Supplementary Figure 9**d**), indicating that etching near the interface The liquid has reacted completely. This data also shows that the chemical reaction rate in the H_2O_2/Ge system is greater than the diffusion rate, and it is a diffusion-controlled solid-liquid reaction system. For various sacrificial layer patterns, the area where the concentration gradient of the etchant exists is about 50-70 μm (Supplementary Figures 7,8). This diffusion distance is only affected by the ratio of chemical reaction rate and diffusion rate. When the reaction rate is slow, the amount of material consumed will be less than the diffusion rate, resulting in a shorter concentration gradient area, as shown in Supplementary Figure 4**a**. In summary, although the shapes of the sacrificial layer will affect the local distribution of concentration, it will always follow the principle of maximizing the flux of the etchant along the normal direction of the solid-liquid interface. Its overall distribution only depends on the ratio of chemical reaction to diffusion rate,

proving broad applicability of this multi-layer FEM model.

Secondly, we have used FEM to demonstrate the impact on etching when there is a high-speed external flow field, which will not occur in the usual wet etching process (Supplementary Figure 3**a**). We first analyzed the flow velocity field during etching in a completely stationary liquid, where the reference point is shown as the red dot in Supplementary Figure 10. FEM results show that the total flow rate is negligible during completely stationary etching (Supplementary Figure 9**b**). However, in the actual process, the generation and disappearance of bubbles and other disturbances will make this situation difficult to be achieved. Therefore, we established a transient model based on the changes in the liquid flow velocity field when the bubbles rise to observe the parts that have the most obvious impact on the model in actual experiments. Taking the rising rate of water bubbles in a water tank as an example, we established a piecewise function to simulate the influence of the flow field near the nanomembrane during the rising process of bubbles (Supplementary Figure 9**a**). Therefore, the flow velocity field defined is as shown in the function, and the field is 200 μm from the reference point boundary[8,9] :

$$\mathbf{v}_{\mathrm{flow}} = \begin{cases} 0.02\mathbf{x} + 0.02\mathbf{y}\ \mathrm{m\ s^{-1}}, & 0\ \mathrm{s} \le t \le 0.02\ \mathrm{s} \\ 0\ \mathrm{m\ s^{-1}} & 0.02\ \mathrm{s} \le t \le 1\ s \end{cases}, \tag{6}$$

where $\mathbf{x}$ and $\mathbf{y}$ are unit vector in x and y directions. The first section of the function is used to simulate the flow velocity field when the bubble rises, and the second section simulates the flow velocity field when the bubble disappears. At this time, we can see that when the bubbles rise, the flow velocity near the sacrificial layer is only 1 cm s^{-1}. As the bubbles disappear, the flow velocity near the sacrificial layer decreases rapidly. The flow velocity only takes 0.05-0.1 s to drop to the corresponding level, which will only cause minor influence on self-assembly. Therefore, the multilevel FEM model is applicable to sacrificial layers of various shapes (Supplementary Figure 9**c**). The etching process of the sacrificial layer usually lasts for tens of minutes, and the number of oxygen bubbles generated is small, so the impact of the flow rate on the self-assembly will be negligible.

For different pattern designs, we tried triangles (Supplementary Figures 10, 11), semicircles (Supplementary Figures 10, 12), and various parallelograms (Supplementary Figures 10, 13, 14). In triangles and semicircles, the multilevel model only gets tubular structures. In parallelograms with different angles (Supplementary Figure 13) and aspect ratios (Supplementary Figure 14), the model is also designed by designing corresponding etching paths, in which the asymmetry in pattern leads to the assembly of helix structure, and the experimental results once again verified the feasibility of this design and process.

Supplementary Note 5. FEM modeling for various material systems

For the multilevel FEM model, its universality to different material systems will be important for the future development of electronic devices and microrobots. Therefore, we selected three other representative material systems in micro-nano devices for feasibility verification: low-frequency/high-frequency silicon nitride (HF/LF SiN_x) for optical resonators and capacitors[10,11], NiTi nanomembranes for microrobots[12], and VO_2/Cr nanomembrane for bolometers and actuators [13,14]. These three systems include several on-chip and off-chip applications realized by micro-nano 3D structures, and there are also great differences in strain states. The experimental results are also consistent with the simulation of the FEM model.

First, we will demonstrate self-assembly based on HF/LF SiN_x nanomembranes (Supplementary Figure 15). We take this system into the multilevel FEM model for analysis, and the result shows a transition of ring-taper-tube structure. As can be seen from the SEM images, the system based on dual-frequency silicon nitride exhibits a ring-helix-taper-tube structure with size from 2×20 μm^2 to 80×20 μm^2 (2-20/22-28/30-44/46 - 80 μm) and stress gradient of 1500 MPa[15]. Among them, the structural characteristics of the helix are mainly reflected in the asymmetry of the rolling process. We obtained experiment results that are highly consistent with the FEM model, verifying that the structure of self-assembled HF/LF SiN_x is controlled by the boundary and size of the sacrificial layer at the same time. The multilevel model provides an effective means for high-precision design and potential performance control of microstructure inductors and optical microcavity devices.

Subsequently, we demonstrated the preparation of self-assembled structures of NiTi alloy nanomembranes (Supplementary Figure 16). The usual preparation method of NiTi alloy microstructure is based on oblique deposition of photoresist sacrificial layer. Among them, grazing angle deposition can provide a fixed end and a free end for the photoresist through the shadow effect[16]. We use the difference in deposition rate during electron beam evaporation of NiTi nanomembranes to introduce strain differences and use the interlayer strain gradient to achieve self-assembly of the nanomembranes. Since

the etching rate of photoresist in acetone is extremely fast and it is an etching model dominated by the diffusion process, the boundary conditions of the sacrificial layer will be similar to the Si/Cr system. In the FEM model, it is worth noting that under large strains (2~3%[12], 2.5% for FEM model), even if the boundary conditions cause obvious long-side rolling at the lateral sides. When the patterns are fully released, they will still roll unidirectionally from the short end due to the deformation limit of the fixed end, resulting in most of the self-assembly, and the results are all unidirectional tubes. In the experiment, the structures we obtained are all ring-tube structures, and few helix-type structures existed, which verified the applicability of this model to situations where one end is dominant under diffusion-reaction equilibrium. Introducing this multilevel design method will facilitate the mass production of microrobots and the refined design of actuators.

We used VO_2, a type of heat-sensitive functional material, combined with the Cr layer for verification (Supplementary Figure 17). We studied the self-assembled 120 nm VO_2/30 nm Cr system and conducted structural design and prediction of the self-assembled VO_2 nanomembrane patterns of 200×200 μm and 50×500 μm. Due to the large thickness of vanadium oxide, the radii of its ring and tube will increase accordingly. However, patterns with an aspect ratio = 1:1 still exhibit a tube structure. This is because the VO_2 strain is relatively large (0.6~0.8%[14], 0.8% for FEM), which can still cause a significant strain gradient even when the Cr layer is thin, allowing the taper to overcome both ends. The competitive energy barriers of multidirectional rolling in the etching process are finally realized to assemble the tubular structure. Among them, the 200×200 μm self-assembled nanomembrane exhibits the shape of taper and tube, most of which are tube-type structures.

Supplementary Note 6. FEM applicability for different sizes

For 2D nanomembranes, the size effects involved in shrinking and amplification are quite different. For the two-dimensional patterns involved in this manuscript with a size of 2 to 80 μm, we will discuss the two cases separately. When the overall size is reduced by 10 times, the minimum size will be close to 200 nm, while the thickness of the nanomembrane system is about 100 nm, which means that the self-rolling theory and the plane strain/plane stress theory in the simulation will no longer apply. In this condition, the strain perpendicular to the thickness direction will not be negligible (Supplementary Figure 18)[17], that is,

$$\varepsilon_{\mathrm{z}} = \frac{1}{E}\left(\sigma_{\mathrm{z}} - \mu\left(\sigma_{\mathrm{x}} + \sigma_{\mathrm{y}}\right)\right) \neq 0 \tag{7}$$

When the width is equal to the thickness ($W = 0.4$ μm， $t_{\mathrm{Si}} + t_{\mathrm{Cr}} = 0.1$ μm), which will lead to a significant increase in the radius of curvature (Supplementary Figure 19). At the same time, the length of the cantilever beam will be significantly shortened, and its large deformation behavior will also be significantly weakened. It can be seen from the FEM that the patterns with aspect ratio $\frac{W}{L}$ from 0.1 to 2 all exhibit uniaxial bending, and their small deformation and a small distance between the fixed end make it difficult to form multi-directional rolling during the release process.

At the same time, the surface tension and liquid flow in the nano-scale structure will be significantly enhanced, resulting in a significant deterioration in the forming stability and difficulty to control the rolling direction[18], which will also introduce additional considerations for the simulation. Moreover, the sharp reduction in sizes makes the structure preparation a new problem because the conventional photolithography process will make it difficult to process sub-micron-scale patterns, and other processes (such as electron beam lithography) will further increase the process time and cost.

When the size is scaled to 0.5 times, their pattern sizes are considerably increased compared to 0.1 times, but the length of the released cantilever beam is still short, which cannot make the self-assembled Si/Cr nanomembrane roll in a stable state from multiple

directions. Therefore, it will be challenging to fabricate a taper-type structure at this scale (Supplementary Figure 20).

According to the simulation of the multilevel design model, when the pattern size is enlarged 5-10 times, the patterns will tend to roll unidirectionally in multiple directions from each edge, eventually forming a complex 3D structure composed of various tubular structures and local bending (Supplementary Figures 21, 22). For the FEM simulation of large-size patterns, we did not use the contact module. When considering the contact module, the mesh needs to be as small as possible to avoid the problem of the nanomembrane being unable to roll due to collisions of self-rolling meshes, which will significantly extend the calculation time to decades of days. Moreover, we only used the incomplete etching time points in the FEM simulation to show the corresponding insufficient etching structure in the SEM. Only when the width is small (20~40 μm), the nanomembrane tends to form a multi-turn rolled structure. Since the cantilever beams are too long, the multi-turn rolled structure will be difficult to maintain a tight structure, thus forming a structure between helix and ring (Supplementary Figure 21**a**). In the experiment, we observed multi-turn rolled structures and long-side unidirectional rolled structures in the smaller width area, which also means that the disturbance during the release process of the sacrificial layer at large sizes will have a more significant impact on the self-assembly process. At the same time, for broader nanomembrane patterns, the larger bending moment caused by the long edge rolling during the rolling process will cause the Si/Cr nanomembrane and the edge of the Si nanomembrane to tear, thus further affecting the yield.

In summary, the size effect will significantly affect the self-assembly process of pre-strained nanomembranes. At smaller sizes, we need to consider the failure of plane elasticity theory and the weakening of large deformation effects, limiting the diversity of nanomembrane structure assembly. When the size is larger, we need to consider the occurrence of local rolling in multiple directions and possible problems such as tearing and breakage of nanomembrane during experiments, which usually result in lower yields. Although the distribution of microstructures will be affected by size effects, our multilevel model still performs good applicability for structural design and prediction in

the scale range of hundreds of nanometers to hundreds of micrometers.

Supplementary Note 7. Fabrication of Si/Cr bilayer

The fabrication process for the Si/Cr bilayer nanomembranes is shown in Supplementary Figure 23. The samples were fabricated via consist of Ge/Si/Cr (50 nm/60 nm/40 nm) multilayer nanomembrane by e-beam deposition on a 4-inch silicon wafer, in which the Ge layer is used as the sacrificial layer. The lithography step defines the one-end fixed rectangular sacrificial layer area to produce a window for sacrificial layer etching. The length of patterns ranges from 20 to 80 μm with a step of 10 μm, when the width range is 2-80 μm with a step of 2 μm. The photoresist was removed via acetone immersion and supersonic cleaning to avoid surface residue of organic solvents. After rinsing the surface in DI and drying, the Ge sacrificial layer was etched to realize Si/Cr bilayer release. Ge sacrificial layer was removed by wet etching (30% H_2O_2, 75 °C). When the Si/Cr bilayers in protruding area were released, they will be transferred into acetone and dried in critical point dryer in CO_2 to prevent morphological defect caused by conventional drying. SEM imaging was immediately executed to prevent chemical reactions to the exposed Cr and Si, which will introduce unpredictable morphological change over time.

Supplementary Note 8. FEM modeling and strain analysis for different Si/Cr thickness

For the bilayer nanomembrane system, the self-rolling moment is contributed by the strain distribution in the thickness direction and the integral of the thickness between the nanomembranes, so the thickness of Si and Cr will affect the radius structure after the moment balance of the system. Therefore, we designed a series of Si/Cr nanomembrane models with different thickness combinations and calculated the radius distribution under different thicknesses of Si and Cr layers with the same pre-strain using Nikishkov's curvature formula[19]. The Si/Cr systems we designed with different parameters are Si nanomembranes (15/30/90 nm, Supplementary Figures 24, 25, 26, respectively) and Cr nanomembranes (10/20/60 nm, Supplementary Figures 27, 28, 29, respectively) of different thicknesses. The above samples are shown in Supplementary Table 1.

We then conducted self-assembly experiments on samples from the above parameters and studied the relationship between morphology-size distribution and tube radius-thickness distribution.

First, we observed the ring-arch-helix-taper-tube structural transition in various types of self-assembled Si/Cr nanomembranes by multilevel models and experiments (Supplementary Figures 24-29). However, according to the structure-width relationship diagram (Supplementary Figure 30), it can be seen that there are obvious differences in the transition regions of each structure. For example, in sample #3, the taper-tube transition occurs at a narrower width W = 36~38 μm, and in sample #4, there is almost no tube structure. As the thickness of the Si nanomembrane continues to increase, the Si/Cr nanomembrane will be prone to forming helix and arch structures (samples #4-#6). It indicates that the structural distribution is significantly related to the system strain during the self-assembly of nanomembranes. Therefore, we started with the radius of various tubular structures to study the strain state of the Si/Cr bilayer nanomembrane.

We used the above experiments and the force-moment balance[19,20] formula to estimate the strain magnitude in the Si/Cr layer, that is

$$R = \frac{(E_{\mathrm{Si}})^2 t_{\mathrm{Si}}^4 + (E_{\mathrm{Cr}})^2 t_{\mathrm{Cr}}^4 + 2E_{\mathrm{Si}}E_{\mathrm{Cr}}t_{\mathrm{Si}}t_{\mathrm{Cr}}\left(2t_{\mathrm{Si}}^2 + 2t_{\mathrm{Cr}}^2 + 3t_{\mathrm{Si}}t_{\mathrm{Cr}}\right)}{6E_{\mathrm{Si}}E_{\mathrm{Cr}}t_{\mathrm{Si}}t_{\mathrm{Cr}}(t_{\mathrm{Si}}+t_{\mathrm{Cr}})\Delta\varepsilon}, \quad (8)$$

where E is Young's modules, t is thickness of nanomembrane, and$\Delta\varepsilon = \varepsilon_{\mathrm{Cr}} - \varepsilon_{\mathrm{Si}}$ is the initial strain difference between Si and Cr layer We substitute the set thickness and tube radius distributions into this formula for fitting. The relationship between the tube radius and the thickness of the nanomembrane we obtained is shown in the Supplementary Figures 31**a, b**, in which the prestrain of the Cr layer obtained by fitting is $\varepsilon_{\mathrm{Cr}} \sim 0.65\%$, the pre-strain of the Si layer is approximately$\varepsilon_{\mathrm{Si}} \sim -0.55\%$. The corresponding stress gradient is approximately$\Delta\sigma = \varepsilon_{\mathrm{Cr}}E_{\mathrm{Cr}} - \varepsilon_{\mathrm{Si}}E_{\mathrm{Si}} \approx 2600$ MPa, which is consistent with the preset conditions in our simulation ($\Delta\sigma = 2000$ MPa) is highly close, providing an important guarantee for high-accuracy structural design. We can see that as the thicknesses of Si and Cr nanomembranes continue to increase, the Si/Cr tube radius shows a decreasing-increasing trend. As shown in Supplementary Figure 31**a**, when $t_{\mathrm{Si}} = 60\,\mathrm{nm}$, the Cr thickness continues to increase, the radii of the assembled tubes are 13.21±0.55 μm (sample #1), 8.13±0.34 μm (sample #2), 7.48±0.28 μm (standard sample), and 6.22±0.59 μm (sample #3). As shown in Supplementary Figure 31B, when $t_{Cr} = 40\,nm$ As the Si thickness continues to increase, the radii of the assembled tubes are 6.95±0.93 μm (sample #4), 3.67±0.21 μm (sample #5), 7.48±0.28 μm (standard sample), and 7.06±1.93 μm (sample #6). With the increase in thickness, maintaining the same strain layer requires a more significant strain difference between the two materials. Therefore, when the strain difference between the two materials is constant, a large layer thickness will always lead to an increase in the tube radius. We also obtained the strain distribution in the thickness direction by simulating the same prestrained Si/Cr system with different thicknesses.

In addition, the radius-thickness relationship of each sample obtained by the finite element design model also shows good consistency with experiments and formula calculations. For tubular models of various thicknesses, the multilayer design models all show larger tube radii than the theoretical models. This is because the elastic mechanics model used in the modeling process is a plane stress model, and for wider strip self-assembly, its mechanical behavior will be closer to the plane strain model, and the model can be modified as

$$R = \frac{(E'_{\mathrm{Si}})^2 t_{\mathrm{Si}}^4 + (E'_{\mathrm{Cr}})^2 t_{\mathrm{Cr}}^4 + 2E'_{\mathrm{Si}}E'_{\mathrm{Cr}}t_{\mathrm{Si}}t_{\mathrm{Cr}}(2t_{\mathrm{Si}}^2 + 2t_{\mathrm{Cr}}^2 + 3t_{\mathrm{Si}}t_{\mathrm{Cr}})}{6E'_{\mathrm{Si}}E'_{\mathrm{Cr}}t_{\mathrm{Si}}t_{\mathrm{Cr}}(t_{\mathrm{Si}} + t_{\mathrm{Cr}})\Delta\varepsilon'} \quad (9)$$

, where $E'_i = E_i/(1 - \nu_i^2), \Delta\varepsilon' = (1 + \nu_i)\Delta\varepsilon$. The correction of Young's modulus and strain gradient will improve the accuracy of the model in a wider range of situations.

We studied the influence of the thickness and initial strain of the other layer on the average strain in the nanomembrane layer when the parameters of the Si layer and the Cr layer are fixed, respectively. Among them, the average strain is calculated by the following formula:

$$\bar{\varepsilon} = \frac{\int_{t_{i-1}}^{t_i} \varepsilon(z)\, dz}{\sum_{i=1}^{2} t_i} \quad (10)$$

It is used to characterize the strain state and strain size of the entire nanomembrane. First, we chose $t_{\mathrm{Si}} = 60\ \mathrm{nm}, \varepsilon_{\mathrm{Si}} = -0.55\%$ to quantify the effect of t_{Cr} and $\varepsilon_{\mathrm{Cr}}$ (Supplementary Figure 31**c**). It can be seen from the scanning image that when the top layer is Cr, its overall strain state mainly manifests as tensile strain, which is consistent with the mechanical behavior observed in the experiment. When the Si/Cr nanomembrane is released from the substrate, the tensile strain The Cr nanomembrane in the stretched state will be released and will be subject to relative compressive strain. In contrast, the Si nanomembrane will be subject to more tensile strain. As the thickness of the Cr nanomembrane continues to increase, its own strain will dominate the overall strain state of the nanomembrane. For each sample in our experiment, as the Cr thickness increases from 10 nm to 60 nm, the average strain shows a small attenuation from 0.9% to 0.5% and maintains the tensile strain state. It is worth noting that the increase in average strain relative to prestrain mainly originates from the neutral axis position

$$y_b = \frac{\sum_{i=1}^{n} E'_i t_i (y_i + y_{i-1})}{2\sum_{i=1}^{n} E'_i t_i}, \quad (11)$$

$$y_i = \sum_{k=1}^{i} t_i, \quad (12)$$

during rolling. The change in the overall strain will also lead to a gradual increase in the tube radius as the nanomembrane thickness further increases.

Subsequently, we chose $t_{\mathrm{Cr}} = 40\ \mathrm{nm}, \varepsilon_{\mathrm{Cr}} = 0.65\%$ to quantifiy the effect of t_{Si} and $\varepsilon_{\mathrm{Si}}$ (Supplementary Figure 31**d**). The average strain relationship will show multiple rising and falling trends due to the interaction between the tube radius and the neutral axis, the neutral axis, and the thickness of each layer. When $t_{\mathrm{Si}} = 40{\sim}100\ \mathrm{nm}$,The

abnormal prestrain-average strain region that appears mainly comes from the fact that when the neutral axis is located near the Si layer, the bending moments in the layer cancel each other out. When the thickness of the Si layer further increases, the strain within the Si layer is still dominated by its pre-strained state. In the experiment, as the Si nanomembrane thickness increased from 15 nm to 90 nm, the average strain of each sample remained at a compressive strain state of -0.5%~-0.6%, which is highly consistent with the experimental verification and model design of Si/Cr double-layer nanomembranes.

Supplementary Note 9. Calculation model and verification for elastic energy

Verification of elastic energy of released bilayer in FEM modeling need appropriate equation to calculate the model in an analytical way. The calculation to analyze the energy variation during release is based on the plane stress assumption. As the parameters of layer i is set are thickness t_i, Young's modulus E_i, Poisson's ratio and initial strain ε_i^0.The density of elastic energy can be calculat0ed by integrating the elastic energy from the bottom to the top of nanomembrane[19,20]:

$$\bar{u} = \sum_{i=1}^{2} \int_{z_{i-1}}^{z_i} \frac{E_i}{2(1-v_i^2)} \left[\left(\varepsilon_{xi}^2 + \varepsilon_{yi}^2\right) + 2v_i\varepsilon_{xi}\varepsilon_{yi}\right] dz \tag{13}$$

$$z_0 = 0, z_1 = t_1, z_2 = t_2 + t_1 \tag{14}$$

As for the bilayer nanomembrane without release, the strain is

$$\varepsilon_{xi} = \varepsilon_{yi} = \varepsilon_i^0 \tag{15}$$

Simultaneously, we established a pre-strained bilayer with boundary constraint from all sides to simulate the condition (Supplementary Figure 33**a**). The total elastic energy and elastic energy density are shown in Supplementary Figures 33**b, c**, in which the FEM model result and calculation result under plane stress assumption show a high consistency. Besides, calculation result under plane strain assumption is also exhibited, which is higher than the experiment in the same stress. When the nanomembrane self-rolled unidirectionally, we considering that there is no strain relaxation in *y* direction, this issue can be regarded as plane strain problem. So, we have

$$\varepsilon_{yi} = \varepsilon_i^0, \varepsilon_{xi} = \varepsilon_i^0 + \varepsilon_{\text{const}} + \frac{z - z_{\text{b}}}{R} \tag{16}$$

For the strain in *x* direction, is the balanced strain after relaxation, referring to the strain change of neutral plane. And $\frac{z-z_{\text{b}}}{R}$ is the strain induced by geometric deformation, in which z is the coordinate of neutral plane and R is the radius of rolling nanomembrane. The value of $\varepsilon_{\text{const}}$ and z_{b} are given by following equations:

$$\varepsilon_{\mathrm{const}} = -\frac{\sum_{i=1}^{2} E_i^{'} t_i \eta_i \varepsilon_i^0}{\sum_{i=1}^{2} E_i^{'} t_i} \tag{17}$$

$$z_{\mathrm{b}} = \frac{\sum_{i=1}^{2} E_i^{'} t_i (z_i + z_{i-1})}{2\sum_{i=1}^{2} E_i^{'} t_i} \tag{18}$$

For plane strain case,

$$E_i^{'} = \frac{E_i}{1-v_i^2}, \eta_i = 1 + v_i \tag{19}$$

For plane stress case,

$$E_i^{'} = E_i, \eta_i = 1 \tag{20}$$

The radius of rolled-up microtubes can be calculated as

$$R = \frac{2\sum_{i=1}^{2} E_i^{'} t_i \left[z_i^2 + z_{i-1} z_i + z_{i-1}^2 - 3z_{\mathrm{b}}(z_i + z_{i-1} - z_{\mathrm{b}})\right]}{3\sum_{i=1}^{2} E_i^{'} t_i (z_i + z_{i-1} - 2z_{\mathrm{b}})(\varepsilon_{\mathrm{const}} - \eta_i \varepsilon_i^0)} \tag{21}$$

The density of elastic energy among the bilayer is considered equal everywhere ($\bar{u}_0$). Similarly, the energy density after release is also regarded as equal ($\bar{u}_{\mathrm{b}}$). Hence, the variation in total energy derived from the fixed and released parts. We assumed an ideal releasing process that the release boundary is a straight line that perpendicular to the rolling direction, which is reasonable in the rolling towards one direction. Thus, the total elastic energy calculation in Figure 4**b** and Supplementary Figure 33 can be written as:

$$E_{\mathrm{total}} = \bar{u}_{\mathrm{b}} \times S_{\mathrm{released}} + \bar{u}_0 \times S_{\mathrm{fixed}} \tag{22}$$

In unidirectional rolling model, the radius of rolling tube exhibits a decreasing trend with the increase of width. As shown in Supplementary Figure 33**e**, FEM result of rolling model is in the range of the theoretical calculation under plane strain (red dot line) and plane stress (yellow dot line) condition. Moreover, the relative elastic energy in FEM is also consistent with calculation. The elastic energy of release from different direction is collected by FEM simulation.

Supplementary Note 10. Elastic energy calculation for unilateral rolling

To find the trend of the minimal elastic energy shift in one-end fixed bilayer, we also proposed the elastic energy calculation for unilateral-rolling. The model we established is similar to unilateral-rolling in Figure 4**a**. As shown in Supplementary Figures 34**b-g**, the minimal elastic energy shifted from large release angle to small release angle with the decrease of pattern width in both FEM simulation and calculation, indicating that the morphology transition may exist in other release condition.

To verify the correctness of elastic energy calculation algorithms, a unilateral rolled-up ideal model is introduced into FEM simulation. In unilateral rolled-up model, an obvious shift of $\alpha_{\min}$ from 30° to 70° can be observed, which is relative to the proportion of released area in region I. The ideal model indicates that the unilateral-released nanomembranes are prone to roll up along the direction of the fastest energy reduction. For patterns with extremely large size in a certain dimension ($W \ll L$ or $L \gg W$), release from the long side is suitable for a lower elastic energy state, explaining the long-side rolling behavior in previous studies[21,221]. The results of unilateral rolling model obtained from the calculation shown in Supplementary Figure 35**b** closely match the simulation results, which verifies the validity of the algorithm in obtaining the elastic energy of geometric structures through FEM simulation. In contrast, there will be instability in bilateral-released ideal model because of the competition of two main rolling directions. Bifurcation and shape morphing will appear after instability phenomenon to decrease the total elastic energy. Therefore, additional models of thinner patterns of L = 4 μm and L = 8 μm are studied to further understand the change in energy.

Supplementary Note 11. SEM images for standard sample of Si/Cr bilayer

The statistical data of Figure 3**b** comes from arrays of Si/Cr bilayer with different size (*L* = 45 μm, *W* = 2-80 μm), and the SEM images of them are shown below. From the Supplementary Figures 36**a-d**, a complete transition between different morphology can be observed, indicating the morphology transition also exist in pattern with different length.

Supplementary Note 12. Relationship between occurrence possibility of bilayer nanomembrane and relative strain energy

From the statistical data in Supplementary Figure 40, there is a clear correspondence between the minimum elastic energy and the distribution of structural types, and the rolling angle corresponding to the minimum elastic energy represents the direction in which the structure is most possible to roll up. Due to the opposite trend of direction angle α movement when the width is less than W = 10 μm, another type of horizontal coordinate with opposite direction is used in the coordinate system in W = 4, 8, and 10 μm (Supplementary Figure 40**a-c**).

Supplementary Note 13. Tracking of self-assembly process of Si/Cr nanomembrane

As shown in the Figure 4**d**, we obtained the trend of α changing with etching time for various structures in the FEM. They all exhibit a trend of gradually increasing with the etching process. At the end of etching, the obtained α are ~35° (4 μm, ring), ~78° (10 μm, arch), ~64° (20 μm, helix), ~63° (40 μm, taper), ~53° (60 μm, tube), which is also close to the α angle corresponding to the lowest elastic energy we obtained from the energy calculation, which are 30°~40° (ring), 50°~80° (arch), 65°~75° (helix), 50°~65° (taper), 40°~55°(tube). We noticed that at W = 20 μm, the angle-time relationship fluctuated in the early stages of etching, which is caused by a sudden change in the rolling direction when the helix structure switched from an initial bidirectional rolling to a unidirectional rolling. Through the sacrificial layer etching video (Supplementary Movie 3), we also obtained the α-time curves of various structures above. The α obtained at the end of the etching are ~26° (4 μm, ring), ~71° (10 μm, arch), ~56° (20 μm, helix), ~64° (40 μm, taper), and ~40° (60 μm, tube), which are also in the corresponding lowest energy α region (Supplementary Figure 4**e**). It is worth noting that although the α angle at the end of etching is consistent with that predicted by the multilevel model, the α-time relationship during the process is different from the model in the initial stage of etching, which is mainly composed of two reasons. First of all, in the experiments, the etched area is not always released uniformly with the etching time. For example, the assembly of the 40 μm area into the taper shape will mainly occur at the end of etching (Supplementary Movie3). At the same time, the multilevel model stipulates the time required for each quasistatic step is the same (Supplementary Figures 41**c-e**). Secondly, in the experiment, we observed the phenomenon of α first increasing and then decreasing during the initial etching of the W = 60 μm pattern. This is due to the preferential local rolling on both sides of the rectangle during the actual experiment (Supplementary Figures 41**f-h**). It will lead to a change in the dominant rolling direction during initial etching[21,23]. As etching continued, experiments and multilevel designs finally gave similar results, but it also shows that there is room for improvement in the prediction accuracy of the model.

Supplementary Note 14. Formation and distribution of 3D structure array

The large-scale (6 mm ×6 mm) arrays of different structures with nearly 100% yield were prepared to verify the formation stability. The uniformity of structures derives from the stable minimal elastic energy state after release, indicating the relationship between morphology and elastic energy in multilayer system. The statistical data of Table 1 come from the 3D structure arrays.

Supplementary Note 15. Reaction-diffusion model and FEM in bottom etching

In fixed end etching method, the etching direction follow the sacrificial boundary movement, which start from two sides of Si/Cr bilayer. After the converge of boundary, the release direction will turn into the free end to release remaining area. During the release process, wrinkles at two sides can be observed due to the incomplete etching and compressive strain. The wrinkle will hinder the rolling from two sides.

For the wrinkles, a sinusoidal height profile is utilized to approximate the geometrical feature $\zeta_0 = A\sin(kx)$, in which A and k are the amplitude and wavenumber of the wrinkle in the y-direction. We assume the sinusoidal wrinkle feature remains during rolling, and only the amplitude of wrinkle changes, which can be written as $\zeta(\gamma) = (1-\gamma)\zeta_0$. When $\gamma = 0$, the wrinkle is in the initial state of amplitude; when $\gamma = 1$, the wrinkle is locally flat[24,25].

The initial curvature of wrinkle is $c_0(x) \approx - Ak^2\sin(kx)$, and initial in-plane strain is $\varepsilon_0 = \bar{\varepsilon} + (\Delta\varepsilon/t)(z - \zeta_0)$, where $\bar{\varepsilon} = \frac{\varepsilon_1+\varepsilon_2}{2}$, $\Delta\varepsilon = \varepsilon_1 - \varepsilon_2$ (ε_i is the strain of layer *i*), and t is the total thickness of bilayer[9,10]. Bending moment per unit width of nanomembrane is $M_0 = (1+v)D\Delta\varepsilon/t$, where v is Poisson ratio, and $D = Et^3/[12(1-v^2)]$ is bending rigidity of nanomembrane. Relative elastic energy change of flattening wrinkle and rolling from vertical direction can be written as:

$$\Delta u_1(\gamma) = -\frac{M_0^2}{2D(1+\alpha_\gamma)} + \frac{1}{4}D\gamma^2(Ak^2)^2, \quad (23)$$

where $\alpha_\gamma = 6(1-v^2)(1-\gamma^2)A^2/t^2$ represent the contribution of bending rigidity from wrinkles. And the equation includes the energy change in rolling up and wrinkle flattening. We take $A \approx 500$ nm and $k \approx 4\times10^5$ m^{-1}, and then obtain the relative elastic energy during flattening bilayer.

Meanwhile, the rolling from wrinkle direction can be calculated as

$$\Delta u_2 = -M_0^2/(2D). \qquad (24)$$

From Supplementary Figure 43**b**, an energy barrier during flattening process can be observed, indicating the vertical rolling process is blocked, while rolling from wrinkle direction is prevailed. Thus, Si/Cr bilayer rolled up in fixed end etching via wrinkle-induced elastic energy preference.

Supplementary Note 16. Multilevel design model in FEM in top etching

FEM models in free end etching released more area in initial step to recurrent the boundary movement of experiments. Compared with release area of free end, the area of two sides of bilayer can be ignored, and the simulation result is in accordance with SEM images from experiment.

Supplementary Note 17. Design and fabrication of multimorphic structures via controlled local etching

The rectangles we used in the manuscript as a demonstration are due to their moderate symmetry and variable control complexity, so the relationship between the elastic energy of the system and the assembled structure can be obtained more directly in the multilevel design method. For circular or semicircular patterns, the parameters are mainly controlled by the radius. The relationship between the self-assembly behavior lacks the aspect ratio as a parameter to diversify the structure type. Therefore, it is difficult to obtain enough information to analyze the forming mechanism. For the tilted parallelogram strip, we will introduce length L, width W, and tilt angle α_t to analyze the assembly process. The tilt angle as an additional parameter will increase the data magnitude by one dimension, which may be helpful for detailed analysis of the relationship between the self-assembly and the result, but it will increase the cost of comprehensive simulation and analysis. Here, we exhibit some multilevel design examples and experimental results with patterns of semicircle, triangle, and parallelogram strips to verify the universality of the multilevel model.

Since the circular pattern has a short connection area with the fixed end, it is easy to tear and fall off from the Si fixed end during the etching process. Therefore, we used a semicircular pattern with a longer connection area for research.

As shown in Supplementary Figure 45, we have set models for semicircles with different radii R, isosceles right triangles with different diagonal lengths D, parallelogram strips with the same included angle α_{t} and different aspect ratios $\frac{W}{L}$, and parallelogram strips with the same length-to-width ratio at different included angles. FEM analysis and experiments are conducted, confirming the broad applicability of the model (an example of the sacrificial layer etching animation of the above pattern is attached to the Supplementary Movie1 and S2). For the top etching (Supplementary Figures 45**g-i**) and bottom etching (Supplementary Figures 45**j-l**) processes, we used the same method as modeling rectangular patterns, that is, adding a preliminary release area for the top etching and stepwise etching from both sides to the top for the bottom etching.

For semicircles (Supplementary Figures 12, 46, 47) and isosceles right triangles (Supplementary Figures 11, 48, 49), we only obtained tubular structures under immersive-etched conditions in both FEM simulations and experiments, because the nanomembranes tended to roll in one direction. It is related to the fact that the etching path always tends to form local long-end rolls at the free end. It is difficult to control multiple etching directions during the etching process, and thus, it is hard for semicircles and triangles to form multi-stable microstructures. For acute-angled isosceles triangles, various types of etching also show similar results (Supplementary Figures 50-52). Among them, the helix morphology formed under immersive etching exhibits more asymmetry due to its asymmetry in all directions etching. An unstable local state is more likely to exist in the isotropic etching path, causing asymmetric rolling in the initial etching process.

We believe that polymorphic assembly can also be realized in other forms, but the premise is that there are at least two low-strain elastic energy states in the assembly structures to form a polymorphic state[26]. Meanwhile, there should be a large enough energy barrier for switching between to ensure that the states are stable[27,28]. Taking a parallelogram pattern as an example, we assume the ideal etching direction is in different etching directions β. When passing through a fixed point in the strip, if an assembling structure wants to switch from the longer diagonal side rolling to the shorter diagonal side rolling, a higher strain energy state will exist when the rolling direction is changed, hindering the transition between the two stable states (Supplementary Figure 53). Therefore, only patterns with multi-stable state and a sufficiently large strain energy barrier (such as parallelogram strips and rectangles with moderate tilt angles) can realize multiple structural designs under the same precursor. For semicircles and isosceles right triangles, the length of the free end is short within the characteristic size of 2 to 80 μm, making it difficult to form a potential barrier that effectively separates the two forms, so there is no multi-stable structure.

A parallelogram with the same angle but a changed length-to-width ratio will roll

along different diagonals due to the difference in spatial order during oblique etching (Supplementary Figures 13, 54, 55). When etching occurs from the bottom, the wide edge at the top will be fixed during the initial etching. When finally released, it will tend to exhibit a unidirectional roll perpendicular to the shorter diagonal, thus to form various types of tubes and rings (Supplementary Figure 54). When etching occurs from the top, the rapid expansion of the initial etching boundary in the obtuse angle area at the top will cause self-assembly to occur along the shorter diagonal direction, thus forming a large number of helix structures (Supplementary Figure 55). The switching between the two forms will produce high elastic energy states in other rolling directions as potential barriers, thereby achieving two types of stable structures with apparent differences. We noticed that some Cr nanomembrane areas are affected by photolithography accuracy and local penetration of chromium etchant during micro-nano processing. Therefore, the ends of the parallelogram strips will be inconsistent with the design model. At the same time, some simulations of parallelogram strips have problems that the nanomembrane moves behind the substrate that should have existed in the experiments (Supplementary Figure 54**i**). Although their morphology is consistent with the experiment, this means that for more accurate model predictions, the contact between the self-assembled nanomembrane and the substrate plane needs to be considered. When applying an improved model in the future, the multilevel FEM method can be further refined to improve the feasibility of the application.

For parallelogram strips with the same width-length ratio and different tilt angles, the structural differences will mainly exist in the area with a tilt angle of 30~60° (Supplementary Figures 14, 56, 57). When the tilt angle is too small ($10° \leq \alpha_t < 30°$), the pattern shows a strong dependence on long-side rolling, making it difficult to form various patterns, so both types of etching methods exhibit helix structures. When the tilt angle is moderate ($30° \leq \alpha_t < 60°$), the rolling in different diagonal directions form an effective strain energy barrier, thereby regulating the self-assembly process of the nanomembranes. At this time, the parallelogram strips etched on the bottom end roll along the longer diagonal and form a tube structure. In contrast, the etched tops roll wide edges from the shorter diagonal direction, mainly forming a helix structure. When the tilt

angle is large ($60°\leq\alpha_t<80°$), the length difference between the two diagonals of the parallelogram gradually reduces, and the difference in elastic energy release caused by the two etching methods is not high enough to form different types of structures. It gradually approaches the helix structures in the released rectangular shapes. However, it is worth noting that the difference in etching methods still affects the rolling direction in the initial phase, causing the pattern to form a helix structure with opposite chirality.

Supplementary Note 18. SEM images and 2D angular photodetection for Si/Cr photodetectors

With the additional design of electrode and Si channel, photodetectors with various structures are prepared via photolithography. The preparation process is the same as fabrication of Si/Cr bilayer in Supplementary Figure 23. Patterns of each structure are selected as: L = 40 μm, W = 15 μm (ring), 20 μm (arch), 30 μm (helix), 40 μm (taper), and 80 μm (tube) (Supplementary Figure 58). Due to the lack of Cr layer on Si channel (yellow area), the forming condition of the same structure usually acquire a larger width of patterns. The rules of morphology transition are still followed in the sequence of ring-arch-helix-taper-tube.

Photodetectors with six structures exhibit obvious photoresponse to 520 nm illuminated laser (Supplementary Figure 59**a**). Take the tubular photodetector as an example, the anisotropic photoresponse can be observed in different incident directions. When the laser is illuminated from y axis, the photocurrent shows a sinusoidal feature, which is similar to a planar photoresponse. In direction of x axis, the omnidirectional photoresponse indicated by a stable photocurrent from 40° to 150° (Supplementary Figures 59**b, c**).

Supplementary Note 19. Setup of incident light directional detection

In order to facilitate readers' understanding, we have supplemented the process of setting up the test platform. As shown in the Supplementary Figure 60, the laser is driven by DC power supply 1, in which the signal generator generates a square wave signal to modulate the laser to achieve the emission of optical signals of a specified frequency. Currently, the Si/Cr photodetector driven by another DC power source detects the optical signal emitted by the laser and generates the current signal. The current signal will be input into the lock-in amplifier and the oscilloscope respectively through the preamplifier, where the lock-in amplifier is used to record the photocurrent intensity, and the oscilloscope is used to record the photoresponse waveform and measure the response time. First, we fix the 3D photodetector to be tested on the optical platform, which has been connected to the PCB circuit board through the bonding machine, and the PCB circuit board is connected to the semiconductor test unit (Supplementary Figures 61**a-c**). The optical image of the omnidirectional light controller is shown in Supplementary Figure 61**d**. It consists of fixed grooves distributed according to spherical coordinates and a detachable SMA optical fiber interface. The fixed grooves are distributed at intervals of 10° according to longitude and latitude, and they interface with the SMA optical fiber. After the combination, the laser can be connected to perform photoelectric testing on the sample in the center of the spherical shell.

Then, we place the incident light controller on the optical platform and perform XY plane alignment and Z-axis alignment, respectively, to ensure that the incident light (90°, 0°) orientation is located in the center of the photodetectors on the PCB board and align the height of the z-axis at the bottom of the controller with the photodetectors. Then, connect the 520 nm laser to the required SMA port and use a semiconductor measurement unit to provide voltage to the sample for photodetection of the incident light.

Supplementary Note 20. Photodetection mechanism and performance of Si/Cr photodetectors

We characterized the photodetection properties of the prepared Si/Cr photodetector, as shown in Supplementary Figure 62. We conducted an I-V test on the arch-type Si/Cr photodetector, which showed Schottky contact-dominated carrier transport behavior in the atmospheric environment (Supplementary Figure 62**a**). According to the log**i**~log(V) curve, it can be seen that the device mainly exhibits three types of transmission behavior at 0~10 V, with slopes of 0.61, 0.99 and 1.02 respectively (Supplementary Figure 62**b**). Among them, the slope is 0.61 in the region of 0.05~0.35 V. This is because when the voltage is small, carriers will be easily trapped by surface defects before being transported to the electrode, causing the $\frac{\mathrm{d}\log(I)}{\mathrm{d}\log(V)}$ less than 1. In the region of 0.35~1.35 V, the $\frac{\mathrm{d}\log(I)}{\mathrm{d}\log(V)}$ is 0.99, and the current-voltage shows a linear relationship, but it does not indicate that Si/Cr exhibits ohmic contact. This is because the device exhibits the typical Poole-Frenkel (P-F) emission effect in Region III (1.35-10 V), which is only present in Schottky contacts. The effect mainly originates from the external electric field emitting the trapped electrons and holes to the continuous state related to the intrinsic dislocation of the Schottky barrier layer, and further directional movement of carrier generates current. When carriers in P-F emission enter the semiconductor material side from the Schottky contact metal material layer, they need to pass through the trap energy level in the potential barrier rather than through direct transition. At the same time, P-F emission mainly occurs inside the semiconductor material rather than at the metal-semiconductor interface. Its current density can be expressed as:

$$J = CE_{\mathrm{b}}\exp\left[-\frac{q(\phi_{\mathrm{B}}-\sqrt{qE_{\mathrm{b}}/\pi\varepsilon_0\varepsilon_{\mathrm{s}}})}{k_{\mathrm{B}}T}\right], \quad (25)$$

in which $\phi_B$is the voltage barrier that electrons must cross when moving from one atom to another in a material in a zero electric field, $k_{\mathrm{B}}$is Boltzmann's constant, ε_{s} is the relative dielectric constant of the barrier layer, T is the temperature, E_{b} is the external electric field strength, which is a constant. Taking the natural logarithm of both sides of this formula gives:

$$\ln(J/E_{\mathrm{b}}) = \frac{q}{kT}\sqrt{\frac{qE_{\mathrm{b}}}{\pi\varepsilon_0\varepsilon_{\mathrm{s}}}} - \frac{q\phi_{\mathrm{B}}}{kT} + ln\,C. \tag{26}$$

There is

$$\ln(J/E_{\mathrm{b}}) \propto \sqrt{E_{\mathrm{b}}}, \tag{27}$$

and combine the linear relationship of

$$J = \sigma E. \tag{28}$$

Multiply both ends by the cross-sectional area of the device A, we have

$$I = JA = \sigma AE = \sigma A\frac{V}{L} = k\sigma V, \tag{29}$$

where k is a constant. The current-voltage relationship and the current density-electric field intensity relationship can be linearly converted, so we can observe the relationship between $\ln(I/V) - \sqrt{V}$ to analyze the P-F emission in the device[29]. As shown in Supplementary Figure 62**c**, the $\ln(I/V) - \sqrt{V}$ curve in Region III shows excellent linearity, confirming that the device is a Schottky contact. Therefore, the linear region in Region II is mainly related to non-ideal factors in the device. Due to the large series resistance, the exponential current characteristics have not yet been demonstrated in this voltage region. At this time, the Schottky junction I-V characteristic equation will be written as[30]:

$$I = I_0\left[\exp\left(\frac{q(V-IR_{\mathrm{s}})}{nkT}\right) - 1\right], \tag{30}$$

$$I_0 = AA^{*}T^2\exp(-q\phi_{\mathrm{b}}/kT), \tag{31}$$

when $V \sim IR_{\mathrm{s}}$ exists, the exponential term of the above formula can be expanded as:

$$I \sim I_0 \cdot \left(\frac{q(V-IR_{\mathrm{s}})}{nkT} + 1\right) - 1 = I_0 \cdot \frac{q(V-IR_{\mathrm{s}})}{nkT}, \tag{32}$$

then $I = \frac{V}{\frac{nkT}{I_0 q} + R_{\mathrm{s}}} = c_0 V$, where c_0 is a constant. Taking the logarithm of both sides gives $\log(I) = \log(V) + \log(c_0)$, in which the slope is $\frac{\mathrm{d}\log(I)}{\mathrm{d}\log(V)} = 1$. Therefore, the larger series resistance may be the reason for the Region II slope of 1. Due to the lack of C-V testing and variable temperature testing platforms, we are currently unable to measure the barrier height of the Si-Cr Schottky contact. We hope that corresponding characterization methods can be introduced in subsequent work for further analysis. Since the voltage in Region I is small, a large number of carriers cannot be moved into the electrode area for collection, and the voltage in Region III is large, which will bring a large dark current and

noise that affects photodetection. Therefore, we chose the operating voltage of the photodetector to be 1 V.

Then, we analyzed the mechanism of the photodetector. The photodetection of this device is mainly based on the resistance change caused by the generation of carriers in the silicon nanomembrane under light rather than the built-in electric potential field of the Schottky junction. We conducted photoresponse tests on the channel area and the Si/Cr Schottky contact area. It can be seen that only the silicon channel area shows an obvious response to light, while the Si-Cr Schottky contact area exhibits no response to light, which means that the Schottky junction is not mainly involved in the photodetection process, and photo carriers mainly originate from the silicon nanomembrane channel (Supplementary Figure 62**d**). Although commercial photodetectors are mainly photovoltaic devices based on p-n junctions, photoconductive photodetectors are also widely used in the direction of photosensitive elements. In the follow-up work, we aim to further optimize device design, develop CMOS technology, and prepare microstructure photodetectors with higher performance.

The thickness of our photodetector is at the nanometer scale, which will exhibit a weak response in mid-infrared wavelength. Therefore, we focused on studying the responsivity-wavelength relationship in the visible spectrum. We tested the responsivity-wavelength relationship of the arch-type Si/Cr photodetector, as shown in Supplementary Figure 62**g**. It can be seen that the detector can effectively detect the visible light range, and its responsivity is 20~62 mA/W in the wavelength range of 400-750 nm. The responsivity shows multiple peaks, which is different from bulk Si devices. The reflection at the silicon oxide/silicon substrate interface will cause periodic fluctuations in the absorption of the Si/Cr detector. We simulated the SiO_2 (35 nm)/Ge (50 nm)/Si (62 nm)/Cr (40 nm) system and found that the absorption rate of Ge/Si/Cr is similar to our device responsivity wavelength relationship, confirming that the nanomembrane system has a certain phase length interference effect on specific wavelength incident light (Supplementary Figure 62**h**). Due to the local rolling of the arch structure, its response band will differ from that of the planar multilayer nanomembrane system.

For various 3D structured Si/Cr photodetectors, the relationship between the responsivity and power is as shown in the Supplementary Figure 62**f**. The Si/Cr 3D photodetector can detect incident light under weaker light, thanks to their 3D structures. The responsivity of photodetectors can reach up to 40 mA W^{-1}, which is slightly lower than commercial silicon-based photodetectors (Supplementary Figure 62**e**). However, various types of Si/Cr photodetectors, including planar structures, exhibit a decrease in responsivity as the power density of the incident light source increases. This may be related to the recombination of photogenerated charge carriers or the enhanced scattering rate as the carrier concentration increases at higher optical power densities. We tested the responsivity-wavelength relationship of the arch-type Si/Cr photodetector, as shown in the Supplementary Figure 62**f**. It can be seen that the detector can effectively detect the visible light range, and its responsivity will gradually increase as the wavelength increases.

At the same time, we tested the responsivity of the Si/Cr photodetector, as shown in the Supplementary Figure 64. The ring, arch, helix, taper, tube, and planar structure photodetectors showed rise times of 120 μs, 138 μs, 389 μs, 758 μs, and 192 μs respectively and decay times of 106 μs, 688 μs, 180 μs, 624 μs, and 197 μs, respectively. The response time of photodetectors is slower than commercial silicon-based detectors and is primarily limited by the lower carrier mobility and presence of impurities in amorphous silicon. It is worth noting that in nanomembranes, the thickness of the semiconductor material is very thin so that the surface defects will dominate the performance of the device, and the nanomembrane released from the sacrificial layer will be contaminated by impurities on both the front and back sides. The above disadvantages will further affect the response time of the photodetectors. Therefore, surface engineering of self-assembled structures will become an important research direction in our subsequent work to improve the performance of 3D photodetectors.

For the photodetectors, their photoconversion efficiency is usually characterized by external quantum efficiency (EQE). We obtained the EQE of the photodetectors through the responsivity calculation. The calculation formula is as follows:

$$\mathrm{EQE} = R_\lambda \cdot \frac{hv}{e} \times 100\% \quad (33)$$

It fluctuates in the range of 7~12% (Supplementary Figure 64), which can effectively detect incident light, but there is still room for improvement. The low responsivity and quantum efficiency of photodetectors are mainly caused by the following reasons. First, the silicon nanomembrane that constitutes the photodetector is thinner and absorbs fewer carriers generated by incident light, resulting in a smaller photocurrent. Secondly, the silicon nanomembrane grown by electron beam evaporation is amorphous, and wet etching during the self-assembly process will introduce additional contamination at the nanomembrane interface. The amorphous structure and a large number of surface states will significantly affect the migration rate of photogenerated carriers from the channel to the electrode and their recombination before they are collected by the electrode, thus reducing the responsivity and prolonging the response time. Our research on this type of 3D structured photodetector mainly focuses on its angle detection and identification, and there is a lot of room for improvement in performance optimization. We have compiled some performance of visible light band detector devices reported in commercial and previous studies (Supplementary Table 2).

In the follow-up research work, we also hope to improve the detection performance of the 3D photodetector further through dry etching, passivation layer coating, and high-quality thin nanomembrane epitaxy. In addition, the free-standing self-rolled microstructure has excellent potential in long-wave infrared detection. The freestanding structure can be realized by using a simple one-step self-rolling process, thereby avoiding the complicated process flow in the traditional bolometer manufacturing process[36].

Supplementary Note 21. FEM parameters, results, and experiments of steady/unsteady reaction-diffusion model

The release of the pre-strained Si/Cr bilayer nanomembrane structure is realized by Abaqus and corresponding scripts. A pre-strained Si/Cr bilayer nanomembranes model is established to simulate and study the morphology after release, in which compressive stress is applied to the Cr layer, and tensile stress was applied to the Si layer to realize the pre-strained state of bilayer. The corresponding parameter can be seen in material and methods. To apply unsteady diffusion reaction model in FEM analysis model, we measured change in etchant concentration. Due to the mass consumption of chemical reaction is relatively small to the thermal compose rate because of small sacrificial area and thickness, we concentrate on the thermal compose of H_2O_2 solution. We prepared a series of culture dishes that contain 30% H_2O_2 with caps, which are weighed before heating. After weighing, they were capped and placed on the heating plate (75 °C), and we took out a dish and opened the cap once to measure the weight loss every 10 min. Because dished were capped during the heating, solution vapor will condense at the bottom of cap, and only oxygen decomposed from H_2O_2 will escape from culture dishes when the cap is open. Herein, we are able to calculate the concentration change of H_2O_2 by measuring the weight loss of culture dishes in different heating times in equation:

$$\Delta c_{\mathrm{H_2O_2}}(\%) = \frac{\Delta m_{\mathrm{O_2}} \cdot M_{\mathrm{H_2O_2}}}{m_{\mathrm{H_2O_2}} \cdot M_{\mathrm{O_2}}} \times 100\% \qquad (34)$$

where m is the weight, and M is the molar mass. And we recorded and graphed the concentration change and weight loss of H_2O_2 during heating within 40 min as Supplementary Figure 65, exhibiting a concentration loss of ~4% hr^{-1}.

In steady diffusion reaction model, change of H_2O_2 concentration is ignored. When the concentration change is negligible, steady diffusion reaction model is also suitable for FEM modeling. We utilized steady diffusion reaction model to simulate the releasing

process of Si/Cr bilayer nanomembrane to compare with the unsteady diffusion model and experimental result. FEM modeling based on steady diffusion reaction model follow the step of Figure 2**a**. Result shows that, simulation based on steady diffusion reaction model is similar to the unsteady diffusion model one, but is less similar to experimental results (Figure 3). It claims that unsteady diffusion model is still the preference to predict the morphology of Si/Cr bilayer nanomembranes.

Supplementary Note 22. Contact module in FEM model

Here, we demonstrate some examples of the contact module in the multilevel FEM model adopted in this study. In the unidirectional rolling process of the traditional FEM model, when the contact module is not applied to the model, the self-assembled nanomembrane will partially overlap (Supplementary Figure 67**a, c**). This phenomenon has less impact on self-assembly models with a single turn or less than a single turn because the self-contact area of such models is smaller. However, the lack of a contact module will cause extremely inaccurate boundary condition results for the analysis of the multi-turn rolling model and further affect the subsequent stress-strain analysis that may be required. The large-scale Si/Cr nanomembrane self-assembly model contains a large number of model self-contacts during the deformation process. As shown in the Supplementary Figure 67**e**, when the contact module is not introduced into the model, it will cause serious distortion of the model, with multiple tubes of self-assembly in different directions, and it is difficult to stabilize. Here, we apply the general contact module to the same model and define the tangential contact mechanism as a penalty function whose form is

$$\tau = \mu\sigma, \quad (35)$$

where τ is the tangential friction force, σ is the normal stress, and the friction coefficient is defined as the dynamic friction coefficient of the Cr-Cr layer $\mu = 0.34$. The normal contact mechanism is hard contact.

As a result, it can be seen that there is no volume overlap of the nanomembranes in the calculation results (Supplementary Figure 67**b, d**). At the same time, its multi-turn self-assembled structure will quickly stabilize due to the existence of the contact volume, reflecting more realistic experimental results. After applying the contact module, we found that the self-contact problem of the model is significantly improved (Supplementary Figure 67**f**), but due to the need to calculate the contact between a large number of small-sized meshes during the quasistatic release of the component, a large amount of computing power is consumed, which will significantly extend the simulation time. Based on the above situation, when the self-assembly process is free from self-contact or only has single-axis curvature single-turn rolling, we mainly use non-contact module operations. When the self-assembly process contains complex deformation or

multi-turn rolling, we will consider the contact module to ensure the accuracy of the results.

Supplementary Note 23. TEM characterization and crystallographic analysis of the Si/Cr nanomembrane

We analyzed the samples by transmission electron microscope (TEM), and it can be seen that there is no coherent interface at the Si/Cr interface, but an obvious non-coherent phase boundary. The above evidence exhibits that the prestrain of Si and Cr bilayer nanomembranes is mainly caused by the temperature difference during deposition and the difference in thermal expansion coefficient of the bilayer nanomembranes after cooling, rather than lattice mismatch.

In order to confirm that the strain gradient between Si/Cr double-layer nanomembranes does not originate from lattice mismatch, we performed focused ion beam (FIB) cutting on the sample and used high-angle annular dark field scanning transmission electron microscopy (HAADF-STEM) and TEM to examine the cross section of the sample. Morphological characterization is performed. As can be seen from Supplementary Figure 68**a**, the sample is composed of Si/SiO_2/Ge nanomembrane/Si nanomembrane/Cr nanomembrane from bottom to top. The carbon protective layer and Pt protective layer on the Cr nanomembrane are FIB cutting samples. The carbon protective layer and Pt protective layer are sprayed on the Cr nanomembrane to protect the cross section of FIB cutting samples. To verify the elemental composition of each material, energy dispersive X-ray spectroscopy is used to conduct elemental analysis of the interface. The distribution diagram of each element is shown in Supplementary Figures 68**b-g**, in which each element shows high signal intensity in the corresponding single-element layer, confirming the elemental composition of each layer in the cross-section. From the analysis of Supplementary Figures 68**b, c**, it can be seen that there is a silicon oxide layer with a thickness of approximately 35 nm above the silicon substrate, which is obtained by thermal oxidation of the silicon wafer substrate. In addition, Supplementary Figure 68**c** shows stronger intensity in the Cr nanomembrane layer. This is because the higher temperature during electron beam evaporation will oxidize the evaporated Cr and the remaining oxygen in the cavity. But in general, chromium nanomembranes have less oxygen adsorption content, which can be seen from optical images and composition

analysis. Since silicon and germanium nanomembranes are also deposited by electron beam evaporation, their EDX images show a small amount of oxygen distribution. As shown in Supplementary Figure 68**g**, the entire nanomembrane system shows a small amount of carbon element distribution. This is due to the slight accumulation of carbon-containing substances and carbon protective layers in the sample during processing and electron beam bombardment. The samples are plasma cleaned, but contamination by carbonaceous species is not completely avoided. This cross-sectional analysis shows the clear interface between the Ge/Si/Cr three-layer nanomembranes, which can be further analyzed by selected area electron diffraction (SAED) techniques to analyze the interface and crystallization state of each material.

As shown in Supplementary Figures 69**a-d**, we used high-resolution transmission electron microscopy (HRTEM) to characterize the morphology of each layer and interfaces. It can be seen that there are obvious interfaces between each layer. In addition, we performed SAED analysis on each layer. Supplementary Figure 69**a** shows the interface diagram between the silicon substrate and silicon oxide. The images show the orderly arrangement of atoms in the silicon substrate without grain boundary. At the same time, Supplementary Figure 69**e** shows the SAED image of the silicon substrate. It can be seen that clear diffraction spots in the direction of the [011] zone axis, in which the silicon (200) and $(11\bar{1})$ crystal plane proves that the substrate is single crystal silicon. In Supplementary Figure 69**b** and Supplementary Figure 69**c**, the HRTEM images of the germanium nanomembrane and silicon nanomembrane do not show regular atomic arrangement. In the SAED images of Supplementary Figure 69**f** and Supplementary Figure 69**g**, there are no sharp diffraction rings corresponding to polycrystalline materials or discrete diffraction spots corresponding to single crystal materials, but diffused diffraction rings, confirming that the germanium nanomembrane and silicon nanomembrane deposited by electron beam evaporation are amorphous nanomembranes. Previous studies have reported growing polycrystalline silicon and polycrystalline germanium thin nanomembranes through electron beams, but it is worth noting that the substrate usually needs to be heated to 400-600 °C[37,38], which will provide sufficient energy for the movement of atoms to the liquid-solid interface during the growth of

silicon and germanium crystals. The electron beam evaporation in this study is deposited on the substrate at room temperature, which causes the atoms to cool immediately after nucleation on the substrate and become unable to continue growing, eventually forming amorphous materials. Supplementary Figure 69**d** shows the HRTEM image of the Si/Cr interface, in which the Si nanomembrane still shows a disordered atomic arrangement, and the atoms in the Cr nanomembrane show obvious grain boundary structures and grains. From the SAED characterization of Supplementary Figure 69**h**, it can be seen that the Cr nanomembrane layer exists in a polycrystalline form, which exhibits obvious diffraction rings, and the diffraction ring distance in the inverted space is highly consistent with the theoretical value[39]. Compared with Si and Ge, which are semiconductor materials, the growth of Cr grains faces a relatively small thermodynamic energy barrier, and their thermal conductivity is also good, which will facilitate the growth during electron beam evaporation and ultimately form a polycrystalline Cr nanomembrane.

In summary, we can conclude that the strain gradient between Si/Cr nanomembranes is not due to lattice mismatch because the Si nanomembranes are amorphous nanomembranes, which do not have an ordered lattice structure, and the Cr nanomembranes are polycrystalline. The lattice constants of the two materials are very different, and they cannot achieve coherent crystal planes. Therefore, we believe that the strain gradient of Si/Cr nanomembranes originates from nanomembrane cooling after electron beam evaporation and the difference in thermal strain caused by different thermal expansion coefficients during cooling process after deposition.

Supplementary Figures

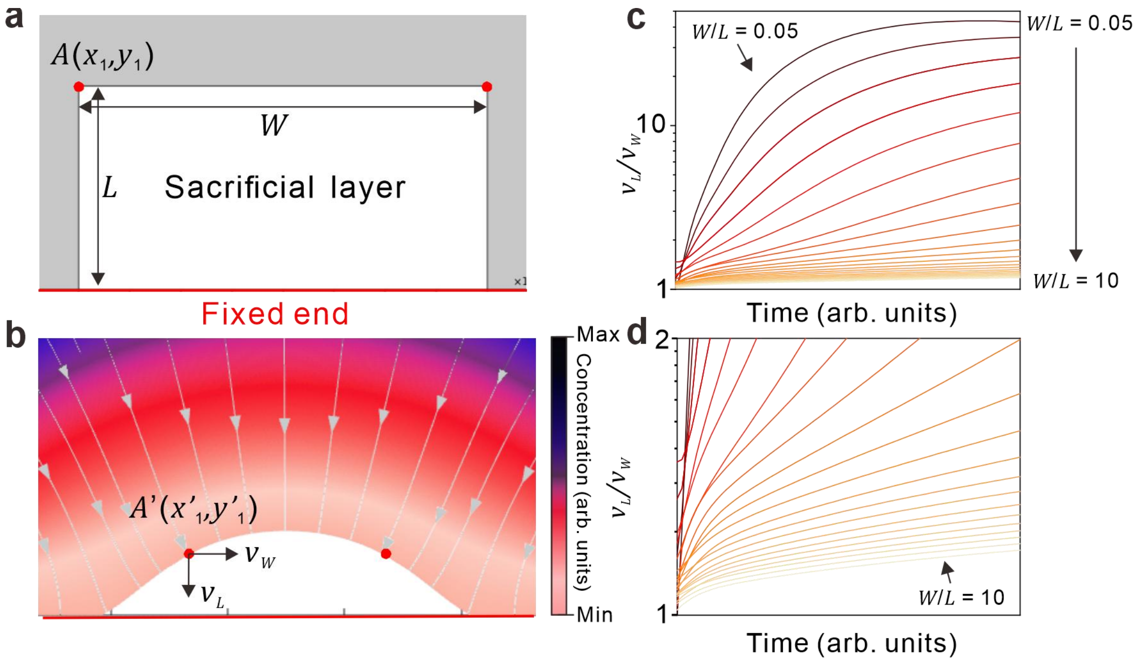

Supplementary Figure 1. Quantitative evaluation of sacrificial layer movement. a Selected mesh vertex for velocity analysis. **b** Movement of mesh vertex and velocity component of different direction. **c**, **d** Velocity ratio of patterns with different aspect ratio.

1077

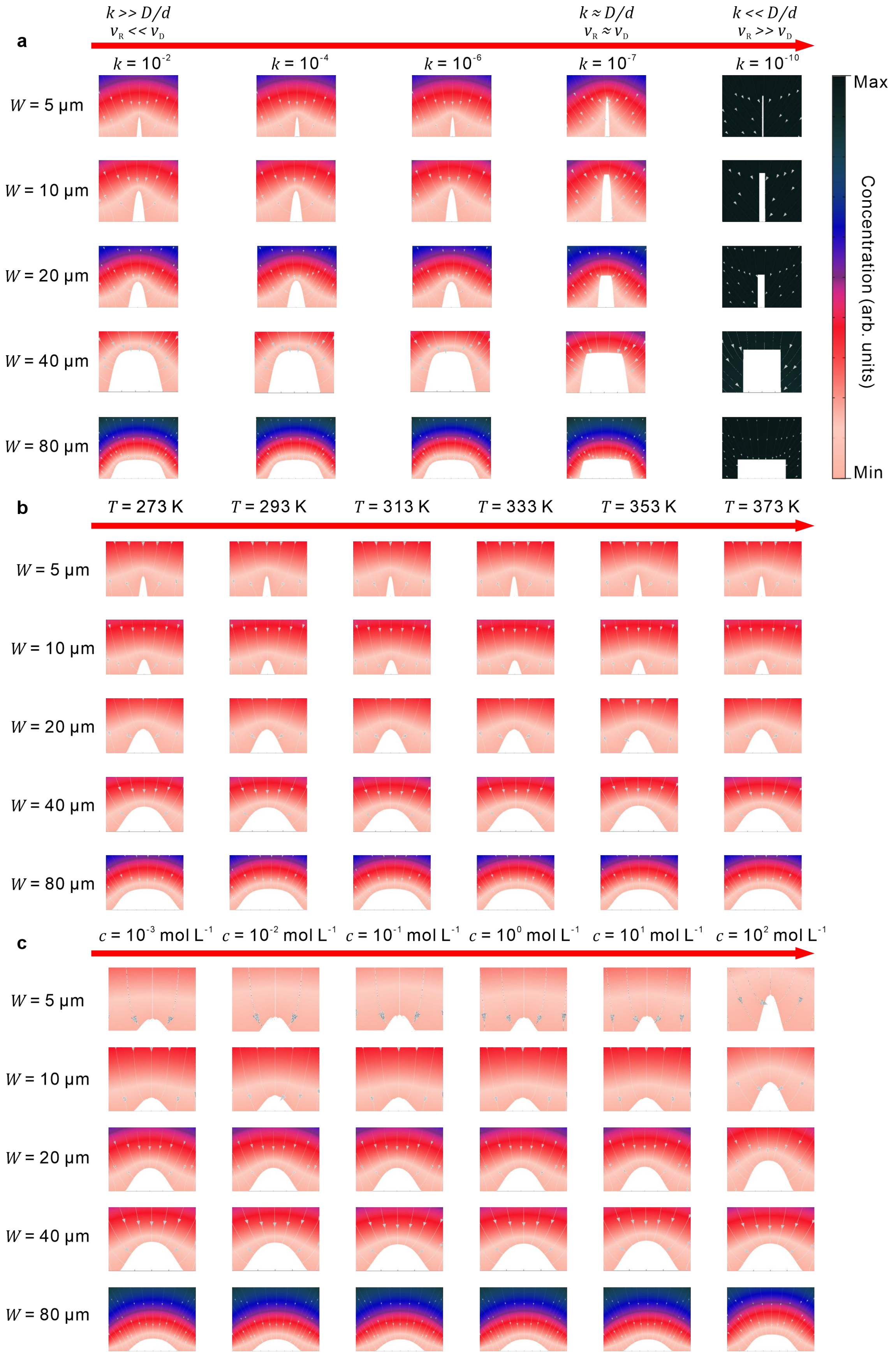

a
k >> D/d
v_R << v_D
k ≈ D/d
v_R ≈ v_D
k << D/d
v_R >> v_D
k = 10^-2
k = 10^-4
k = 10^-6
k = 10^-7
k = 10^-10
W = 5 μm
W = 10 μm
W = 20 μm
W = 40 μm
W = 80 μm
Max
Concentration (arb. units)
Min
b
T = 273 K
T = 293 K
T = 313 K
T = 333 K
T = 353 K
T = 373 K
W = 5 μm
W = 10 μm
W = 20 μm
W = 40 μm
W = 80 μm
c
c = 10^-3 mol L^-1
c = 10^-2 mol L^-1
c = 10^-1 mol L^-1
c = 10^0 mol L^-1
c = 10^1 mol L^-1
c = 10^2 mol L^-1
W = 5 μm
W = 10 μm
W = 20 μm
W = 40 μm
W = 80 μm

Supplementary Figure 2. FEM results of Ge boundary movement via reaction-diffusion model in H_2O_2/Ge system. Effect of **a** rate control steps, **b** temperature, and **c** etchant concentration to boundary of Ge sacrificial layer with different size.

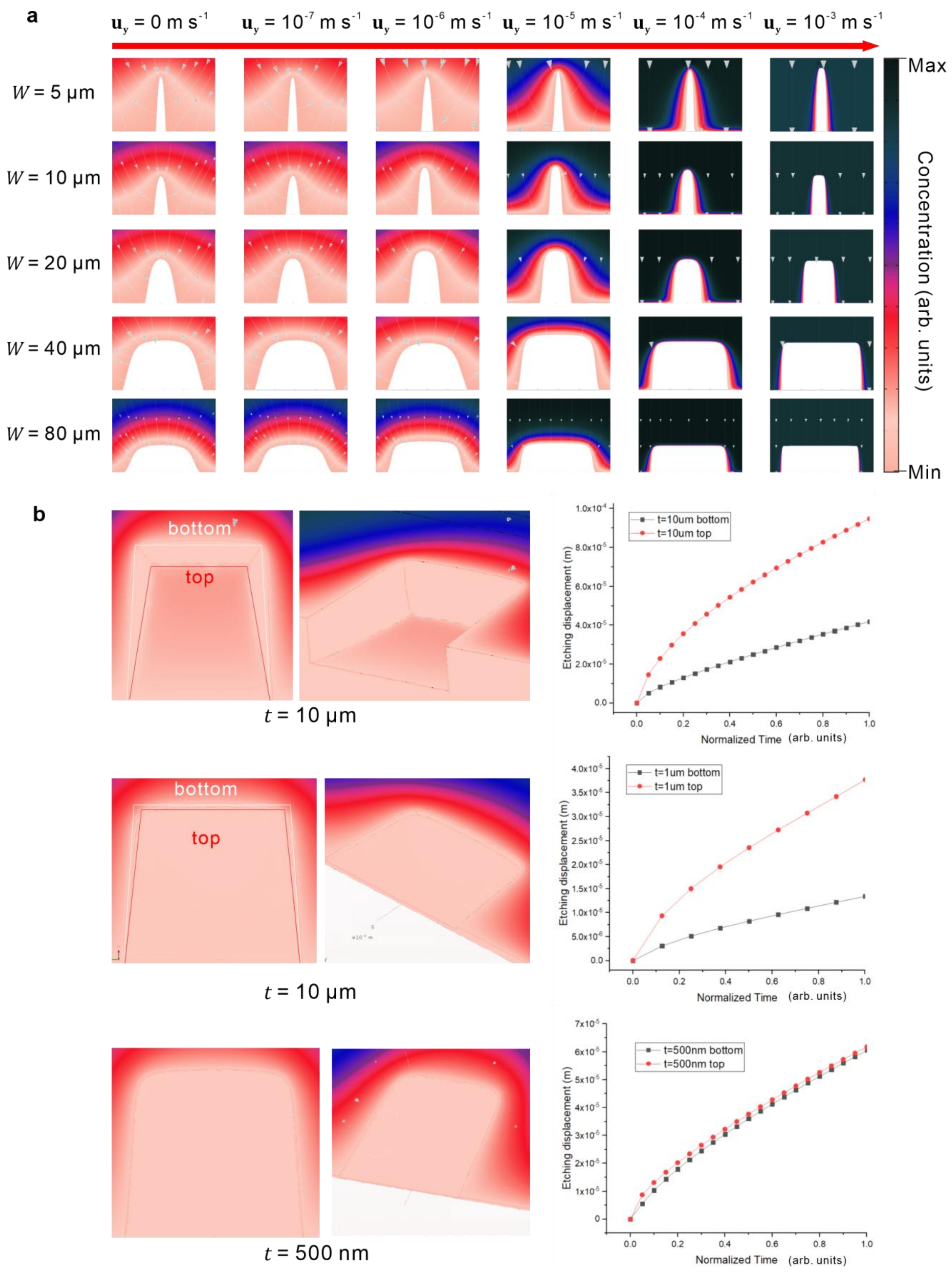

Supplementary Figure 3. FEM results of Ge boundary movement via reaction-diffusion model in H_2O_2/Ge system. Effect of **a** flow field, and **b** thickness of sacrificial

layer to boundary of Ge sacrificial layer with different size.

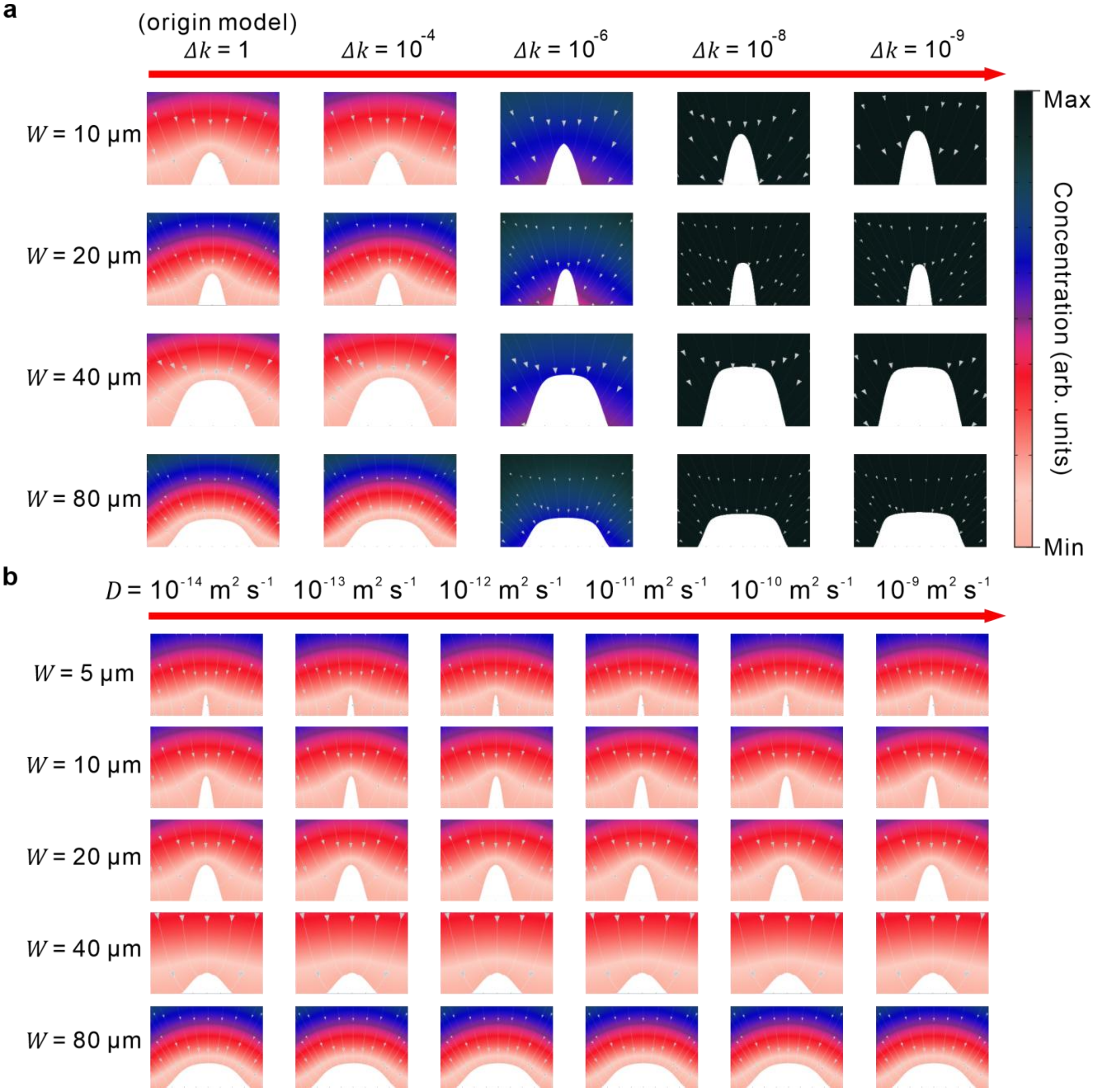

Supplementary Figure 4. FEM results of Ge boundary movement via reaction-diffusion model in H_2O_2/Ge system. Effect of **a** insoluble product generated on surface, and **b** type of material and etchant of sacrificial layer to boundary of Ge sacrificial layer with different size.

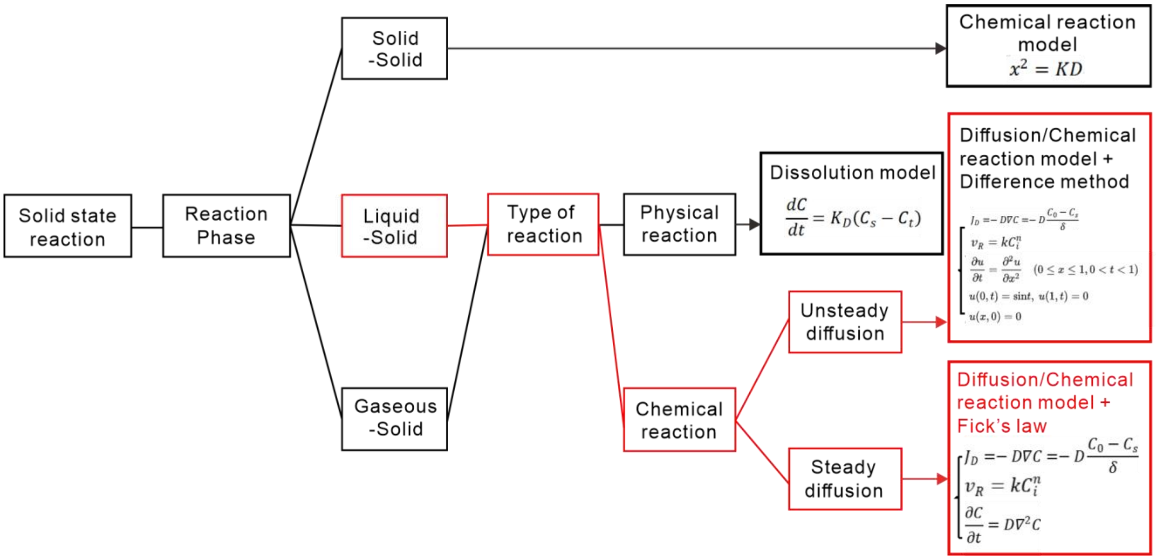

Supplementary Figure 5. Model selection of wet etching in sacrificial layer.

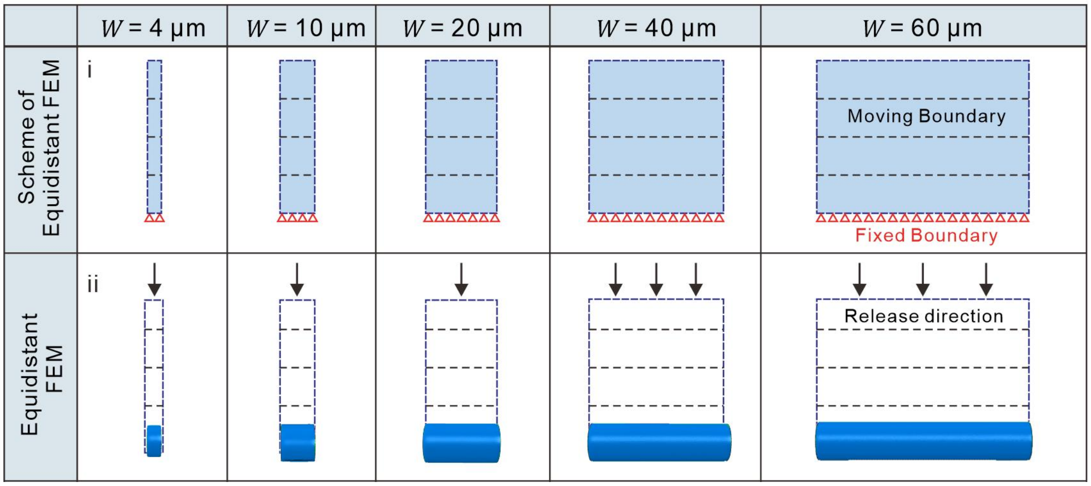

Supplementary Figure 6. Equidistant FEM modeling without quasistatic processes. This method assumes instant releasing bilayer from sacrificial layer. ii Simulation result based on equidistant FEM modeling.

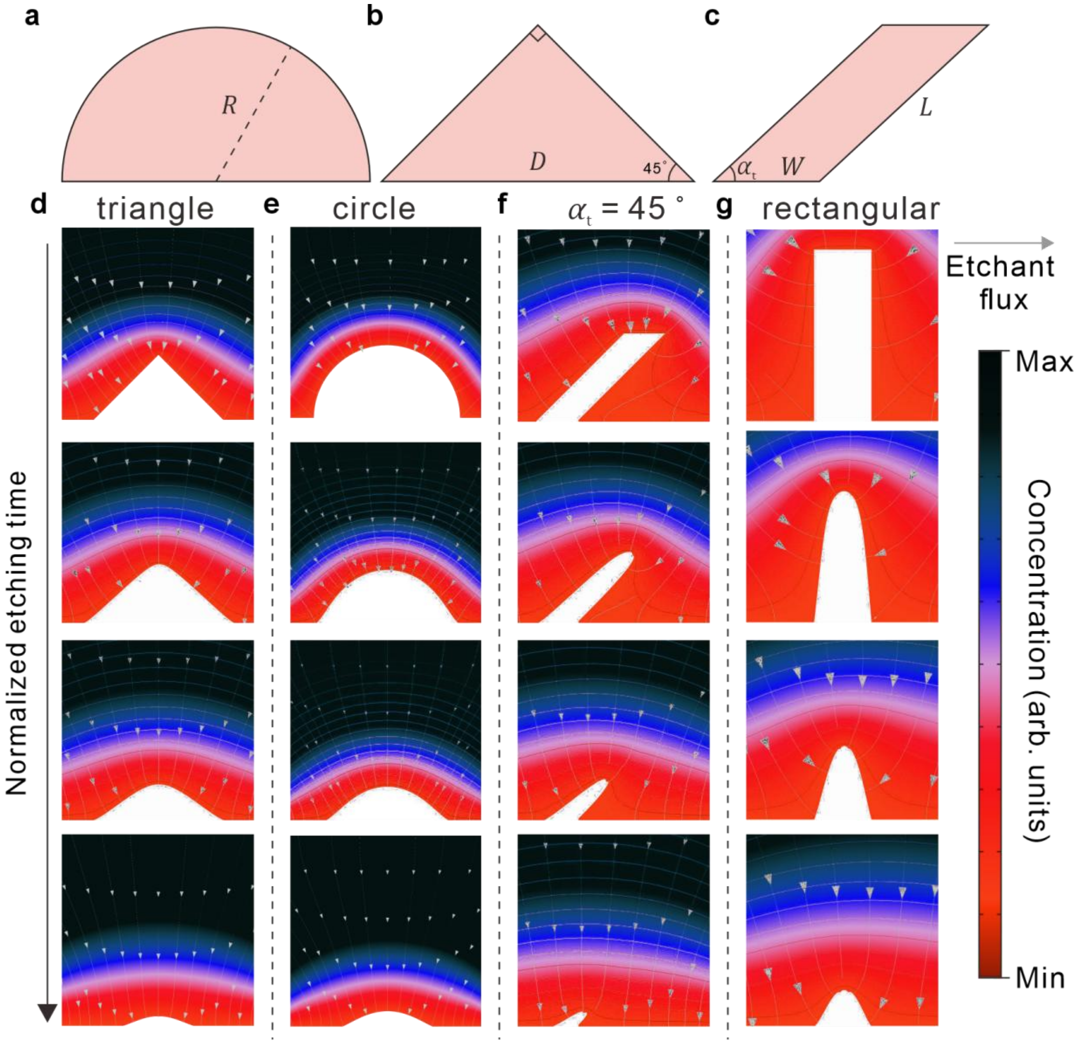

Supplementary Figure 7. Effect of etching pattern on concentration and etchant flux distribution. **a** Schematic diagram of semicircular pattern parameters. **b** Schematic diagram of triangle pattern parameters. **c** Schematic diagram of parallelogram strip parameters. Concentration and etchant flux vector distribution during the etching process of **d** triangle, **e** semicircle, **f** parallelogram, and **g** rectangular strip.

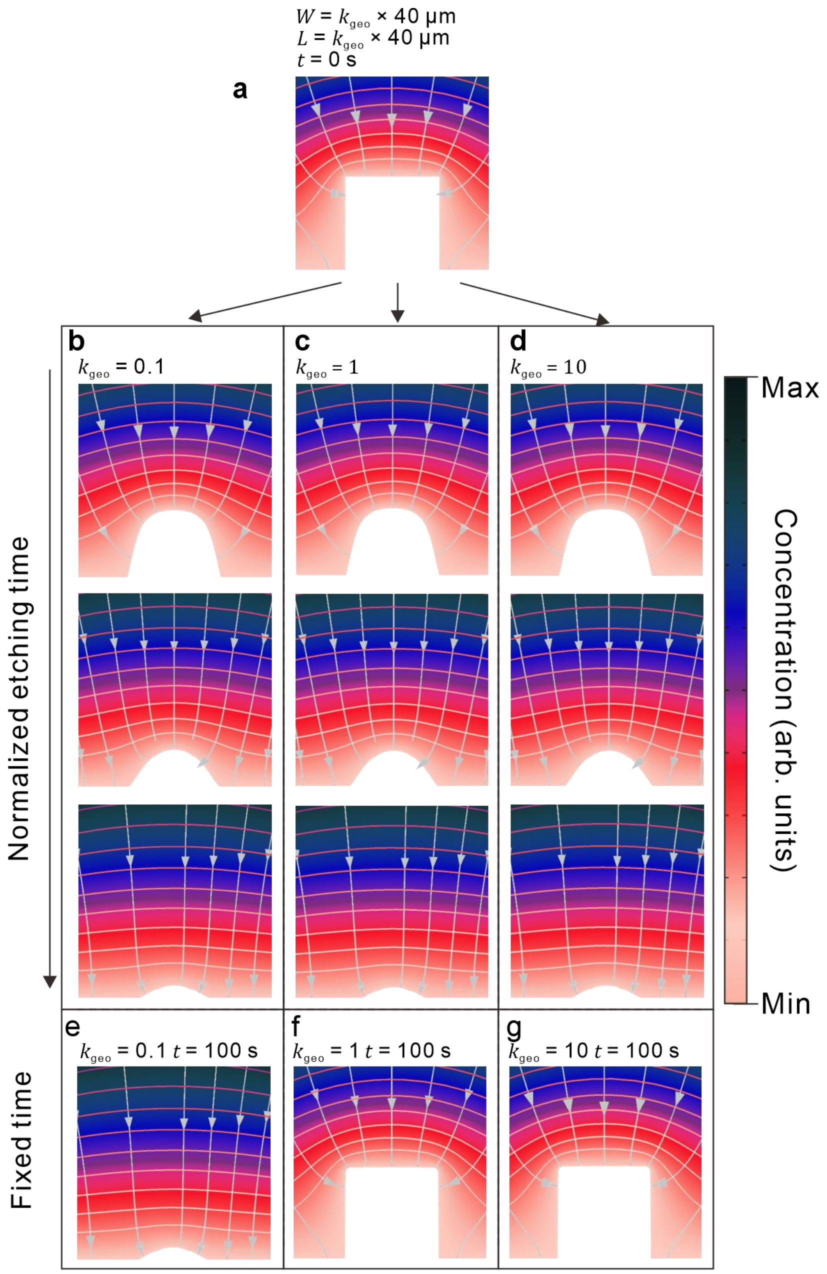

Supplementary Figure 8 Effect of pattern geometry on etching concentration and morphology. a Initial setting of etching pattern. **b** The etching morphology changes and concentration distribution when the pattern size is reduced to 0.1 times. **c** The etching morphology changes and concentration distribution for the original pattern. **d** The etching morphology changes and concentration distribution when the pattern size is reduced to 0.1 times when the pattern size is expanded 10 times. The morphological changes and concentration distribution of the sacrificial layer at the same absolute time **e** when the pattern size is reduced to 0.1 times, **f** the original pattern, and **g** the pattern size is

expanded 10 times.

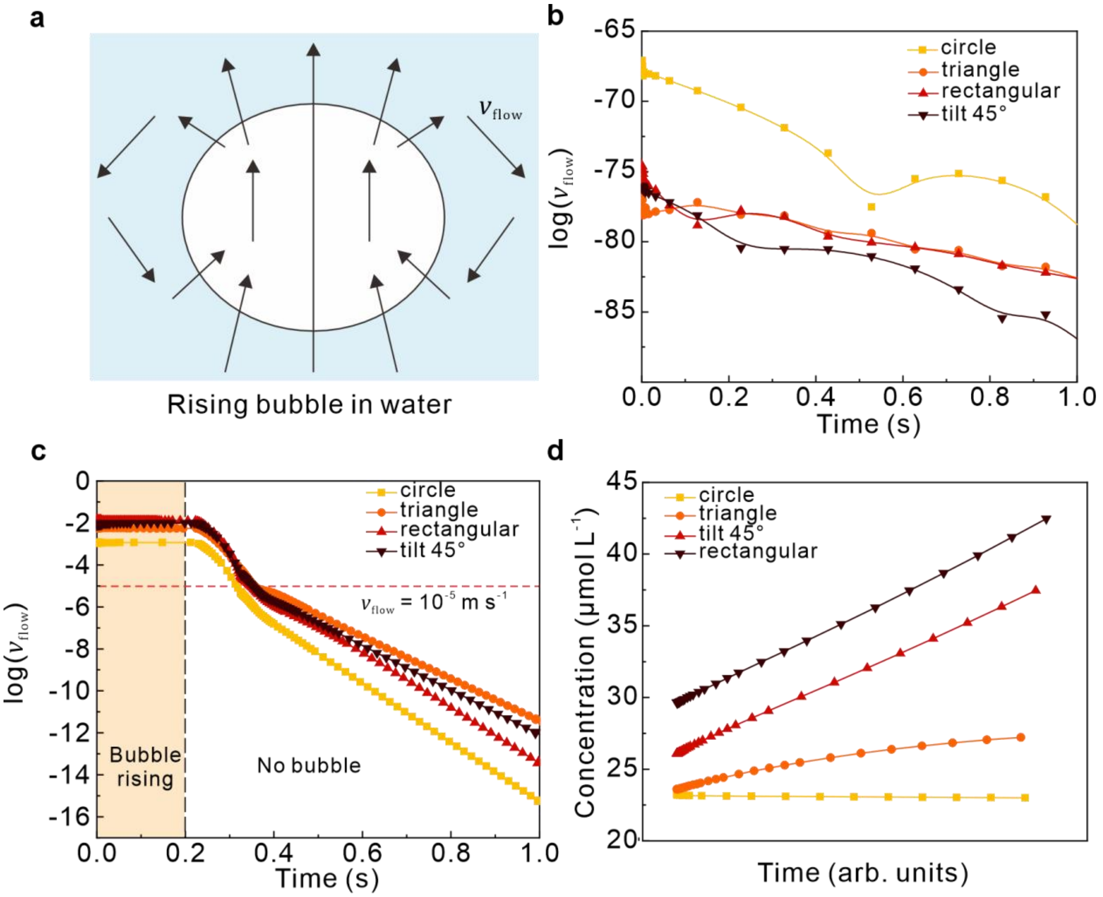

Supplementary Figure 9. Flow rate and concentration distribution of sacrificial layer etching during the actual etching process. a Schematic diagram of the flow velocity field near the bubble during its rise. **b** Flow velocity changes at the measurement point in an ideal stationary fluid environment. **c** Changes in flow velocity at the measure point in the presence of bubble disturbance. **d** Concentration-time relationship of measure points during the etching process.

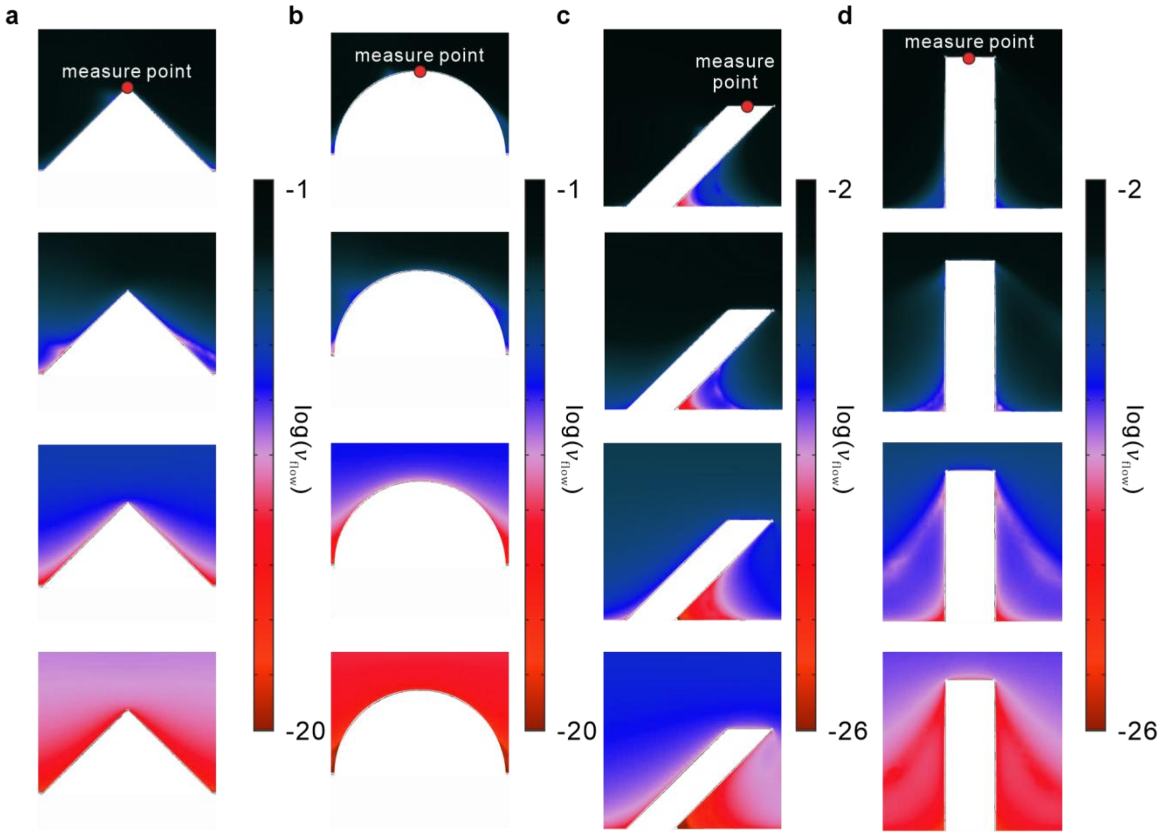

Supplementary Figure 10. Flow velocity and measure point in different patterns. The flow velocity distribution of **a** triangle, **b** semicircle, **c** parallelogram, **d** rectangular strip and corresponding coordinates of measure points during the etching and bubble rising process in Supplementary Figure 9.

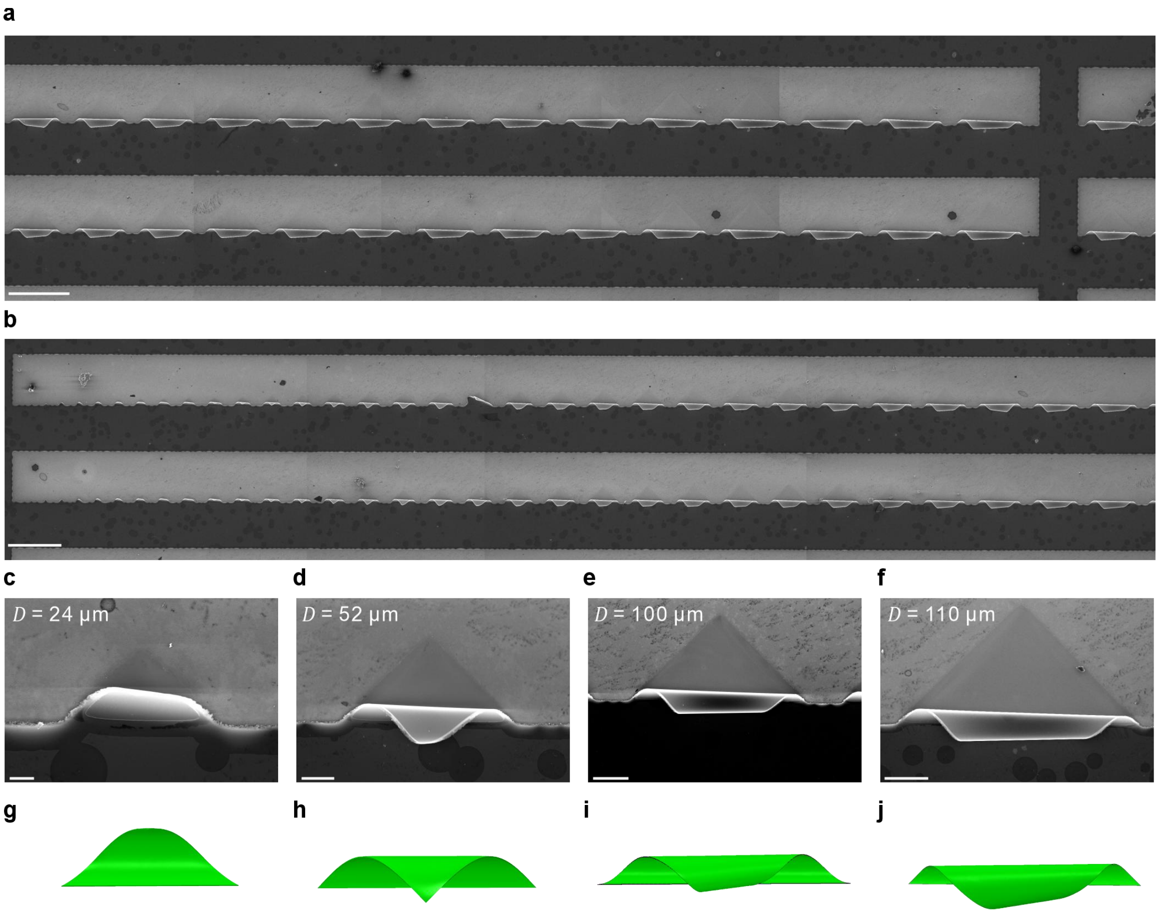

Supplementary Figure 11. SEM images of self-assembled triangle nanomembranes and FEM results of the multilevel design model. a, b Panorama SEM images of self-assembled triangle nanomembranes. Scale bars, 100 μm. SEM images of self-assembled triangle nanomembranes with sizes of **c** D = 24 μm (scale bar, 4 μm), **d** D = 52 μm (scale bar, 10 μm), **e** D = 100 μm (scale bar, 20 μm), and **f** D = 110 μm (scale bar, 20 μm). FEM simulation results of triangle nanomembranes with sizes of **g** D = 24 μm, **h** D = 52 μm, **i** D = 100 μm, and **j** D = 110 μm.

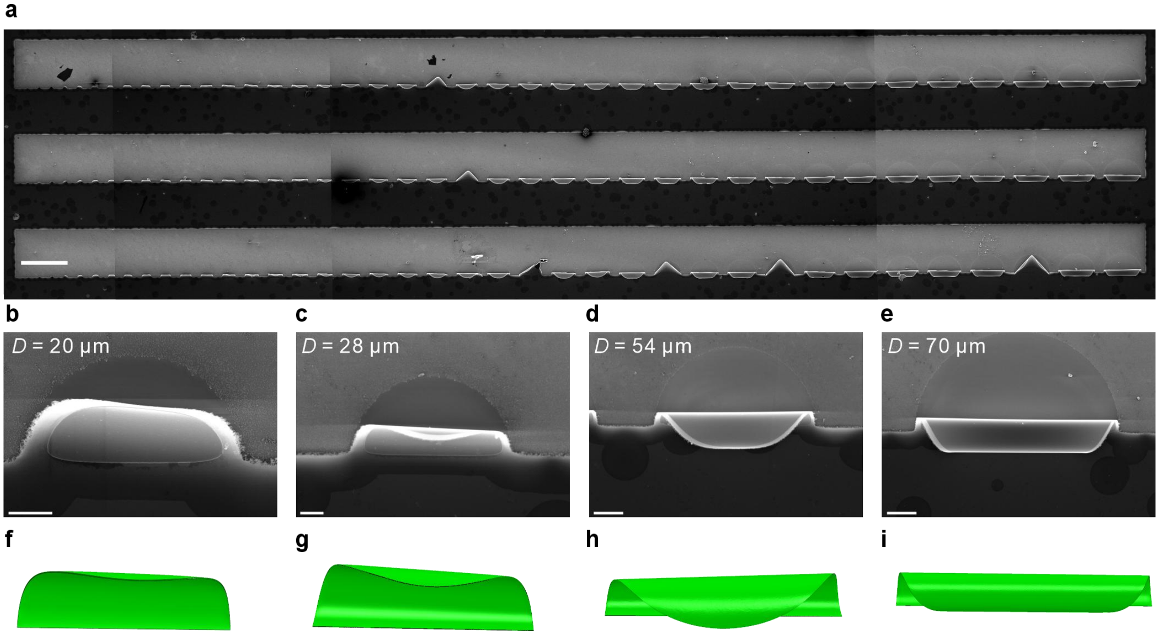

Supplementary Figure 12. SEM images of the self-assembled semicircle nanomembranes and FEM results of the multilevel design model. **a** Panorama SEM images of a self-assembled semicircle. Scale bar, 100 μm. SEM images of self-assembled semicircle nanomembranes with sizes of **b** D = 20 μm (scale bar, 4 μm), **c** D = 28 μm (scale bar, 4 μm), **d** D = 54 μm (scale bar, 10 μm), **e** D = 70 μm (scale bar, 10 μm). FEM results of self-assembled semicircle nanomembranes with sizes of **f** D = 20 μm, **g** D = 28 μm, **h** D = 54 μm, **i** D = 70 μm of self-assembled semicircular FEM simulation results.

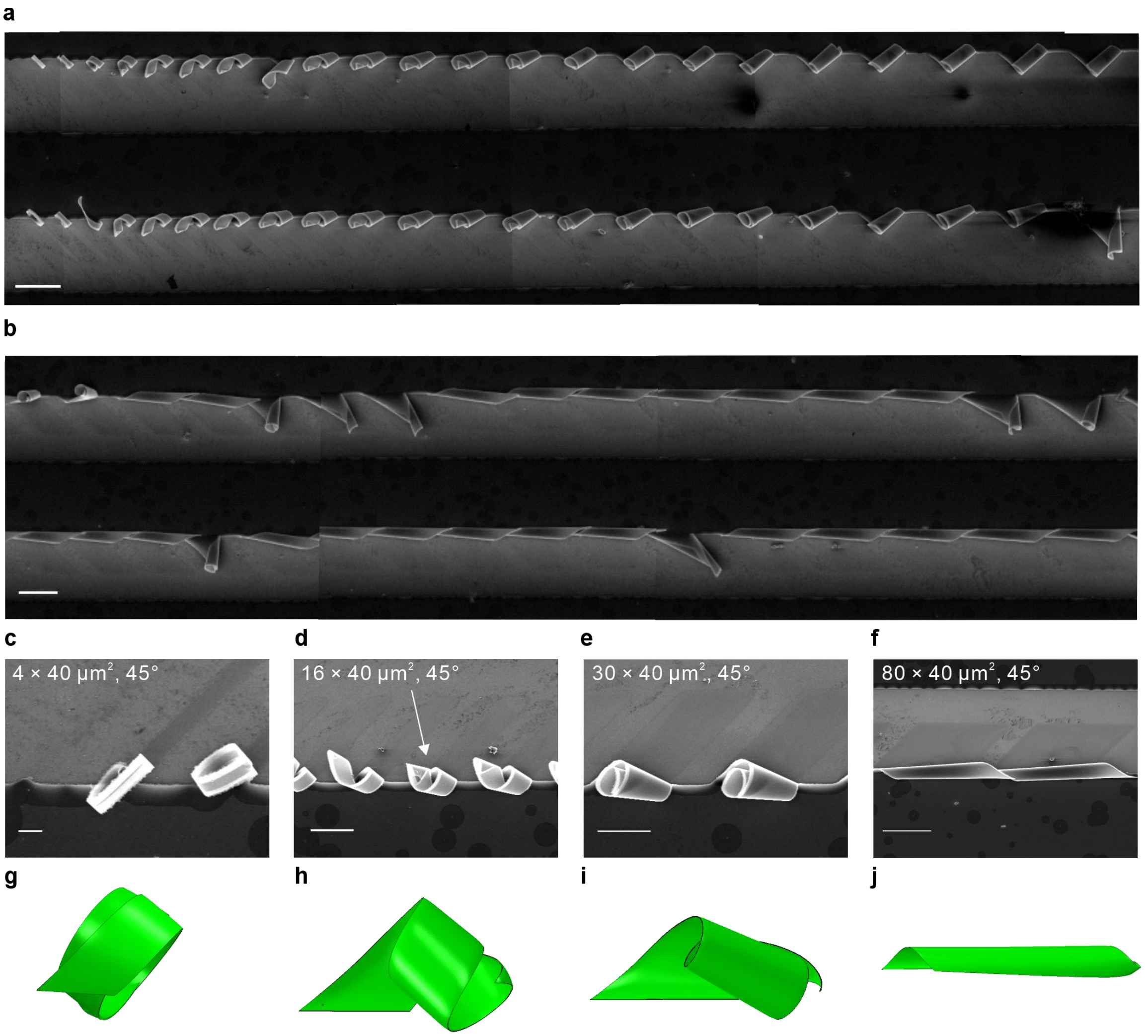

Supplementary Figure 13. SEM images of self-assembled parallelogram strips with different widths and FEM results of the multilevel design model. a, b Panorama SEM images of self-assembled parallelogram strip nanomembranes with different widths. Scale bars, 40 μm. SEM images of self-assembled parallelogram strip nanomembranes with sizes of **c** 4×40 μm^2, α_t = 45° (scale bar, 4 μm), **d** 16×40 μm^2, α_t = 45° (scale bar, 20 μm), **e** 30×40 μm^2, α_t = 45° (scale bar, 30 μm), and **f** 80×40 μm^2, α_t = 45° (scale bar, 4 0μm). FEM simulation results of self-assembled parallelogram strip nanomembranes with sizes of **g** 4×40 μm^2, α_t = 45°, **h** 16×40 μm^2, α_t = 45°, **i** 30×40 μm^2, α_t = 45°, and **j** 80×40 μm^2, α_t = 45°.

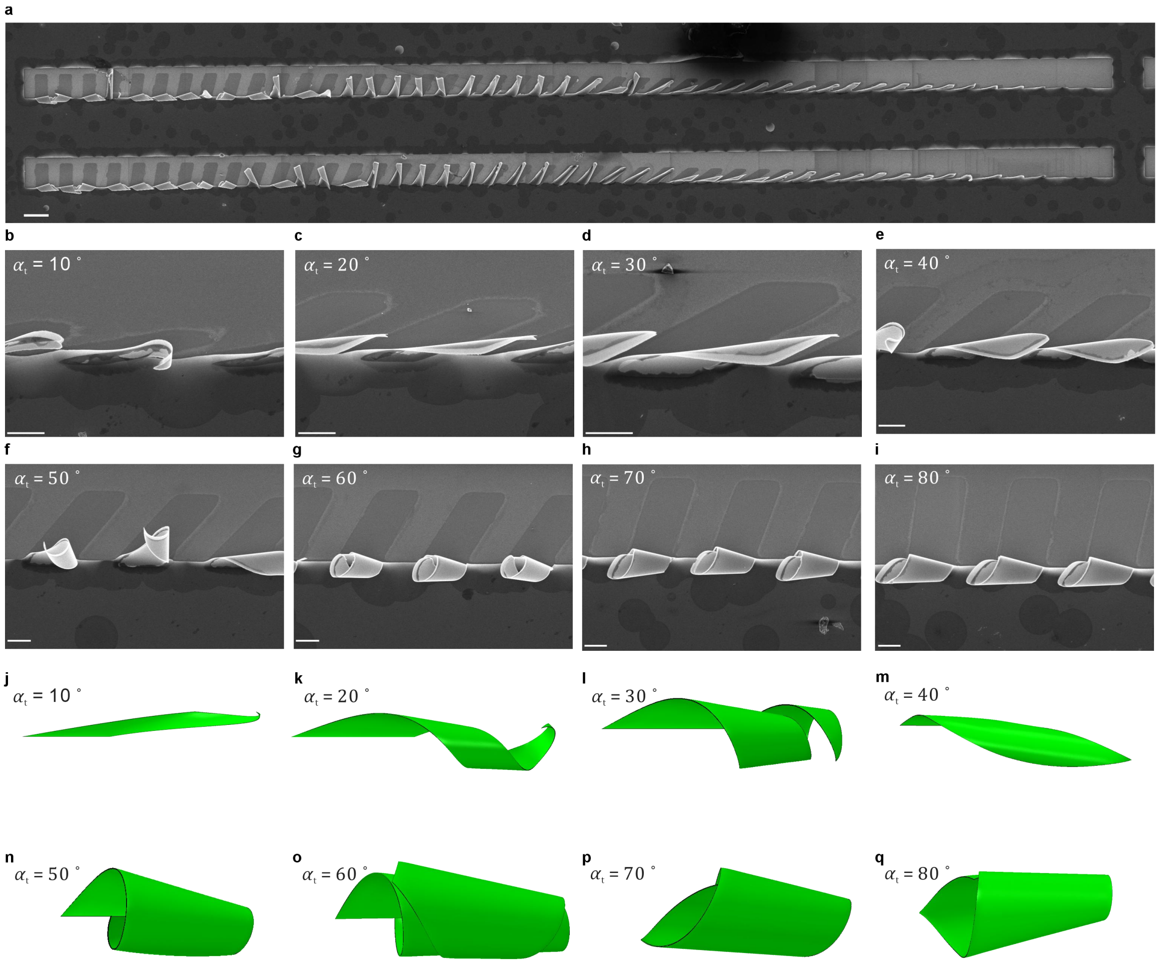

Supplementary Figure 14. SEM images of self-assembled parallelogram strips. a Panorama SEM images of self-assembled parallelogram strips. Scale bars, 40 µm. **b-i** SEM images of parallelogram strips with α_t = 10°-80° (step: 10°). Scale bars, 10 µm. **j-q** FEM results of parallelogram strips with α_t = 10°-80° (step: 10°).

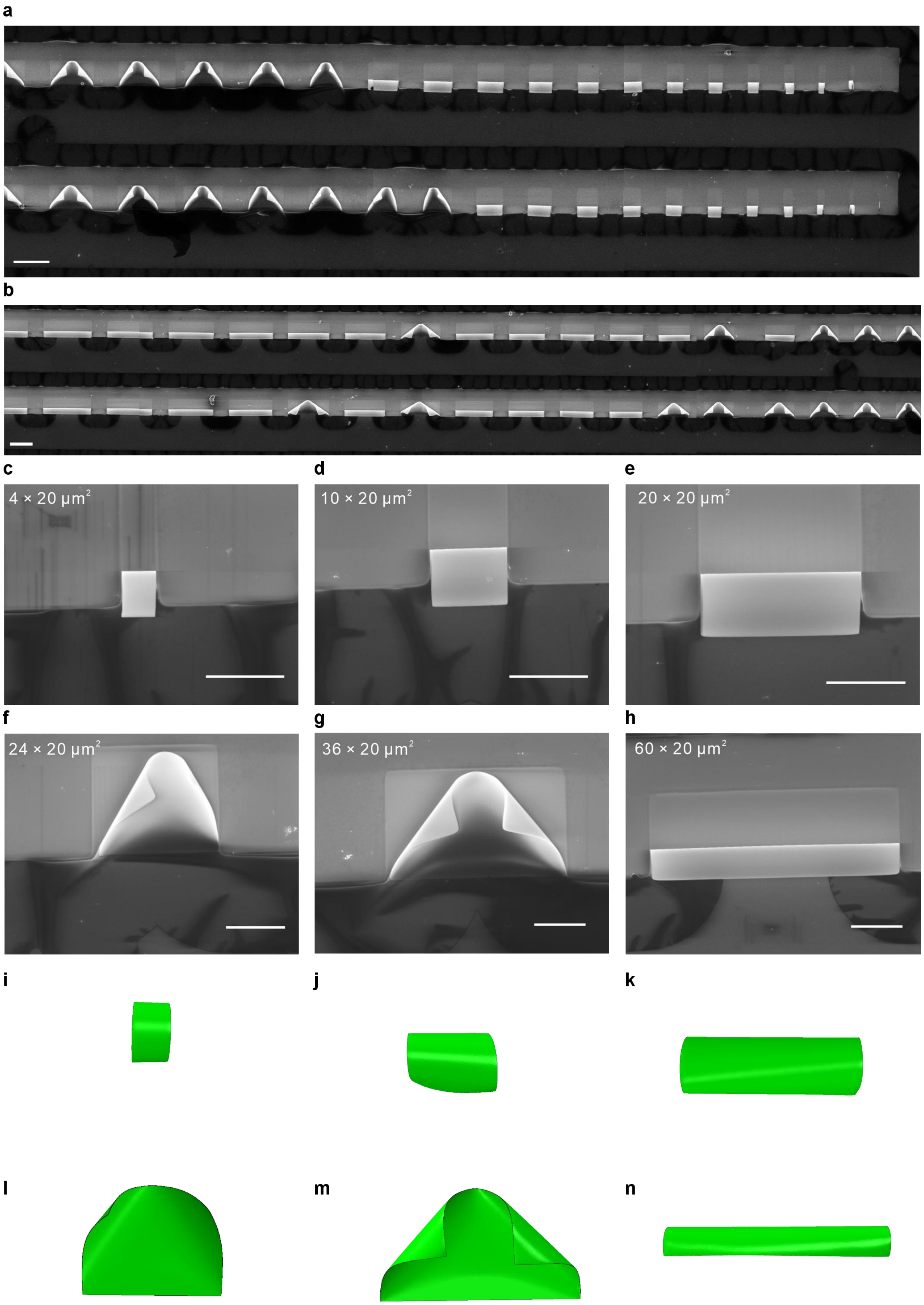

1160

1161 **Supplementary Figure 15. SEM images of self-assembled SiN_x nanomembrane and**

FEM results of the multilevel design model. a, b Panorama SEM images of self-assembled silicon nitride nanomembranes. Scale bars, 30 μm. SEM images of self-assembled SiN_x nanomembrane with sizes of **c** 4×20 μm^2, **d** 10×20 μm^2, **e** 20×20 μm^2, **f** 24×20 μm^2, **g** 36×20 μm^2, **h** 60×20 μm^2. Scale bars, 10 μm. FEM simulation results of self-assembled SiN_x nanomembranes with sizes of **i** 4×20 μm^2, **j** 10×20 μm^2, **k** 20×20 μm^2, **l** 24×20 μm^2, **m** 36×20 μm^2, **n** 60×20 μm^2.

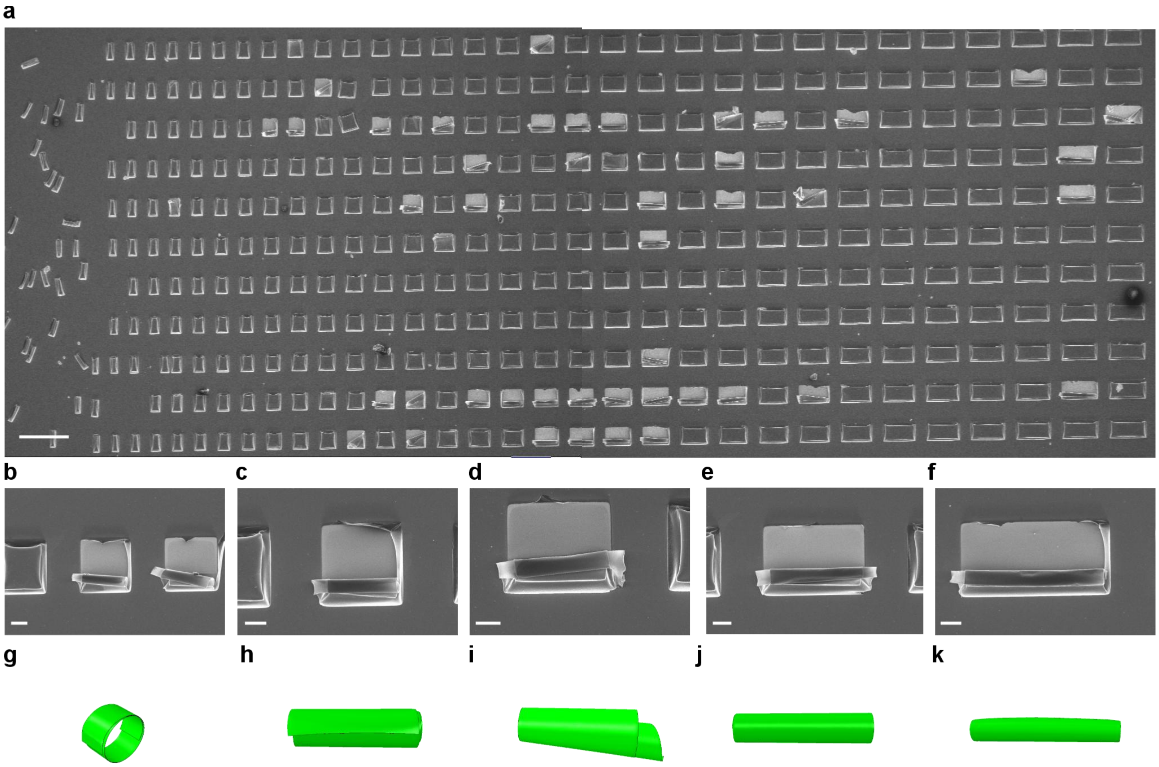

Supplementary Figure 16. SEM images of self-assembled NiTi alloy nanomembrane and FEM results of multilayer design model. a Panorama SEM image of self-assembled NiTi nanomembranes. Scale bar, 100 μm. SEM images of self-assembled nanomembranes with sizes of **b** 30×40 μm^2, **c** 38×40 μm^2, **d** 42×40 μm^2, **e** 60×40 μm^2, and **f** 72×40 μm^2. Scale bars, 10 μm. FEM simulation results of self-assembled nanomembranes with sizes of **g** 4×40 μm^2, **h** 10×40 μm^2, **i** 20×40 μm^2, **j** 40×40 μm^2, and **k** 60×40 μm^2.

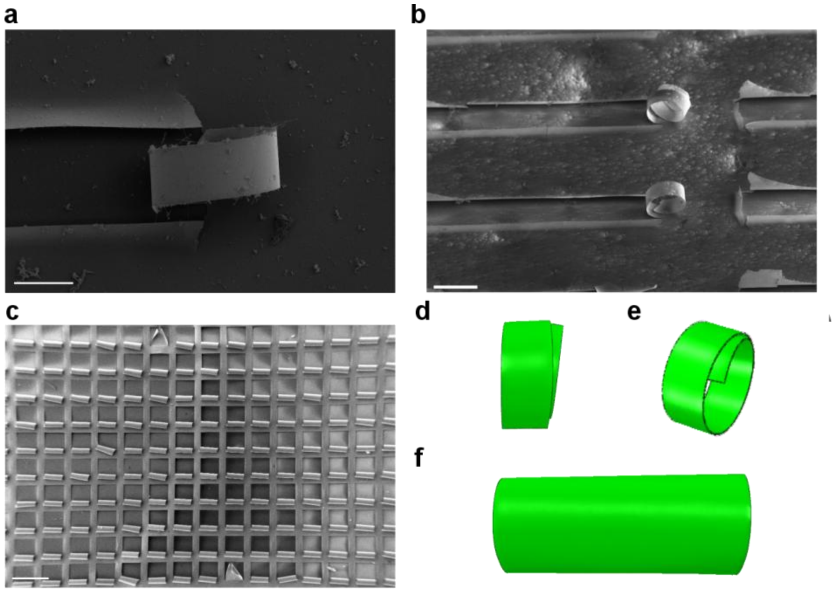

Supplementary Figure 17. SEM images of self-assembled VO_2/Cr nanomembrane and FEM results of the multilayer design model. a, b SEM images of 40×400 μm^2 self-assembled VO_2/Cr nanomembranes. Scale bar of **a**, 40 μm, Scale bar of **b**, 100 μm. **c** SEM image of 200×200 μm^2 self-assembled VO_2/Cr nanomembrane. Scale bars, 400 μm. FEM simulation results of self-assembled nanomembranes with sizes of **d, e** 40×400 μm^2 and **f** 200×200 μm^2

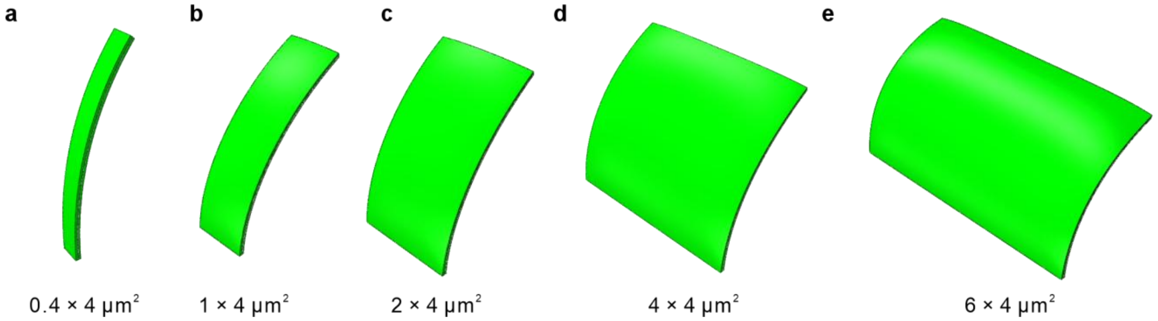

Supplementary Figure 18. FEM simulation results of double-layer rectangular nanomembraneswith a size factor of 0.1 times. Nanomembrane with pattern sizes of **a** 0.4×4 μm^2, **b** 1×4 μm^2, **c** 2×4 μm^2, **d** 4×4 μm, and **e** 6×4 μm^2.

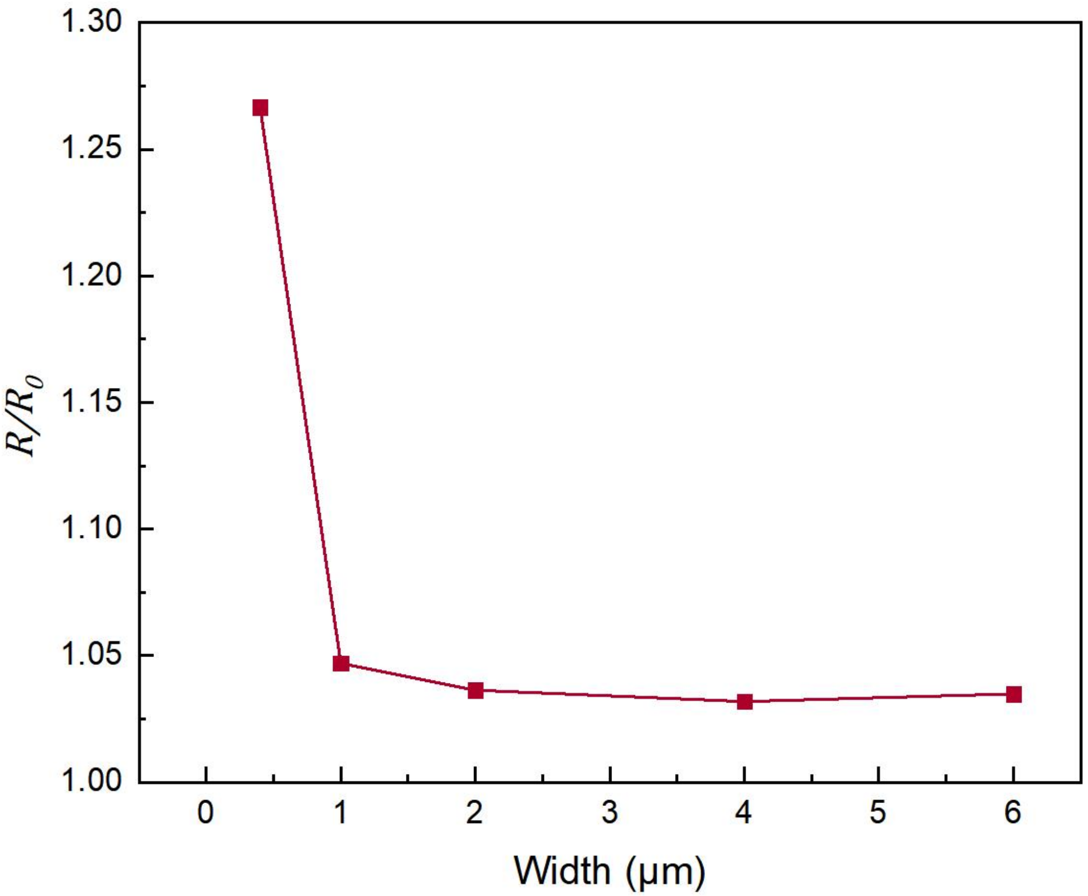

Supplementary Figure 19. Relationship between pattern width and tube radii. Tube radius ratio between the size of (0.4-6) ×4 μm^2 (R) and 60×40 μm^2 (R_0).

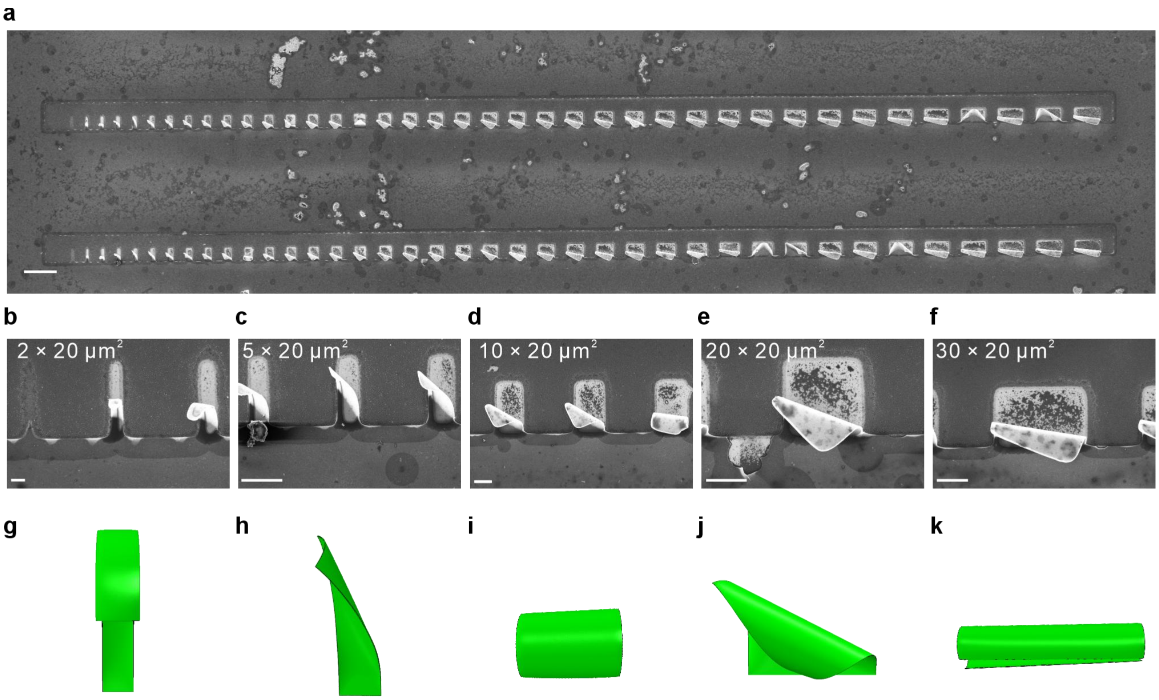

Supplementary Figure 20. SEM images and multilayer design model FEM results of a double-layer rectangular nanomembrane with a size factor of 0.5 times. a Panorama SEM images of self-assembled bilayer rectangular nanomembranes. Scale bar, 50 μm. SEM images double-layer rectangular nanomembranes with sizes of **b** 2×20 μm^2 (scale bar, 2 μm), **c** 5×20 μm^2 (scale bar, 2 μm), **d** 10×20 μm^2 (scale bar, 2 μm), **e** 20×20 μm^2 (scale bar, 2 μm), and **f** 30×20 μm^2 (scale bar, 2 μm). FEM simulation results of double-layer rectangular nanomembranes with sizes of **g** 2×20 μm^2, **h** 5×20 μm^2, **i** 10×20 μm^2, **j** 20×20 μm^2, and **k** 30×20 μm^2.

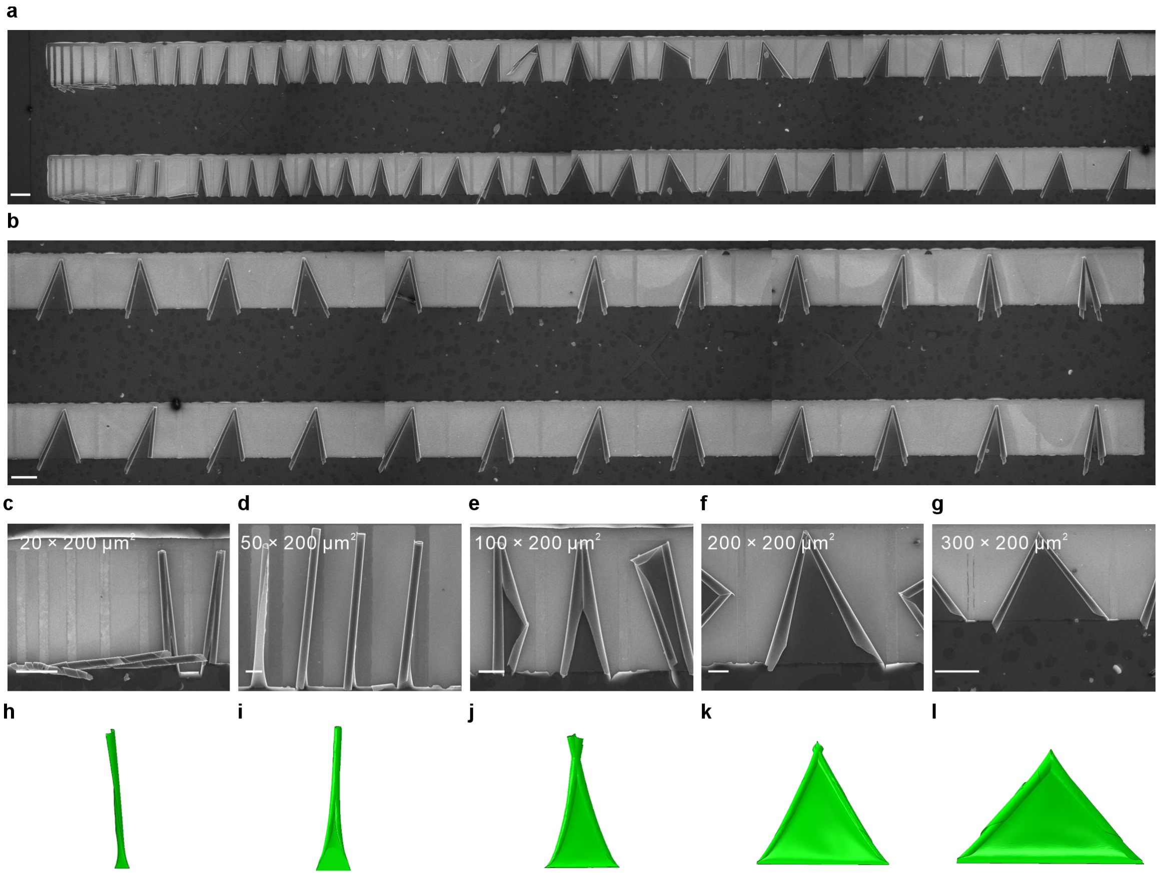

Supplementary Figure 21. SEM images and multilayer design model FEM results of a double-layer rectangular nanomembrane with a size factor of 5 times. a, b Panorama SEM images of self-assembled bilayer rectangular nanomembranes. Scale bars, 100 μm. SEM images of double-layer rectangular nanomembranes with sizes of **c** 20×200 μm^2 (scale bar, 100 μm), **d** 50×200 μm^2 (scale bar, 20 μm), **e** 100×200 μm^2 (scale bar, 40 μm), **f** 200×200 μm^2 (scale bar, 30 μm), and **g** 300×200 μm^2 (scale bar, 100 μm). FEM simulation results of double-layer rectangular nanomembranes with sizes of **h** 20×200 μm^2, **i** 50×200 μm^2, **j** 100×200 μm^2, **k** 200×200 μm^2, and **l** 300×200 μm^2

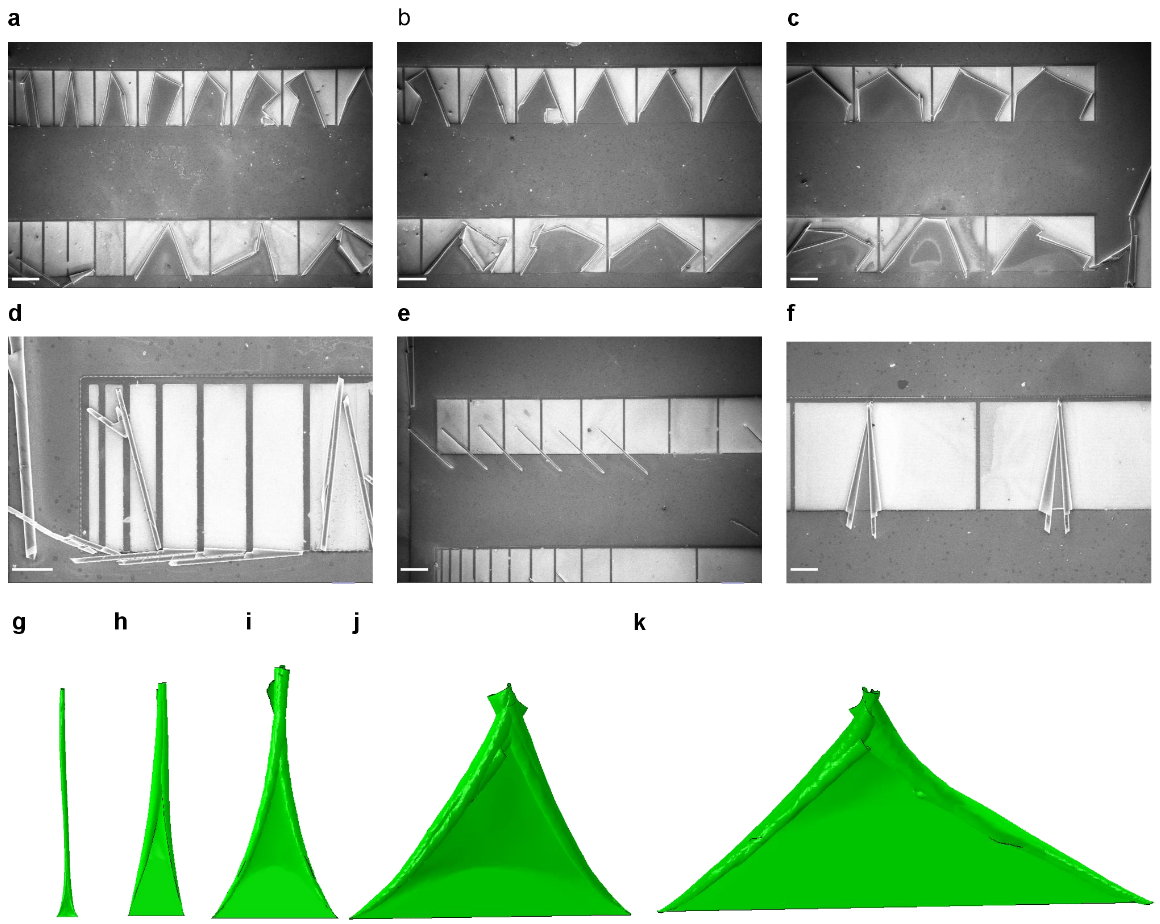

Supplementary Figure 22. SEM images and multilayer design model FEM results of double-layer rectangular nanomembranes with a size factor of 10 times. (A-F) SEM images of double-layer rectangular nanomembranes with a size factor of 10 times. FEM simulation results of double-layer rectangular nanomembranes with sizes of **g** 40×400 μm^2, **h** 100×400 μm^2, **i** 200×400 μm^2, **j** 400×400 μm^2, and **k** 600×400 μm^2.

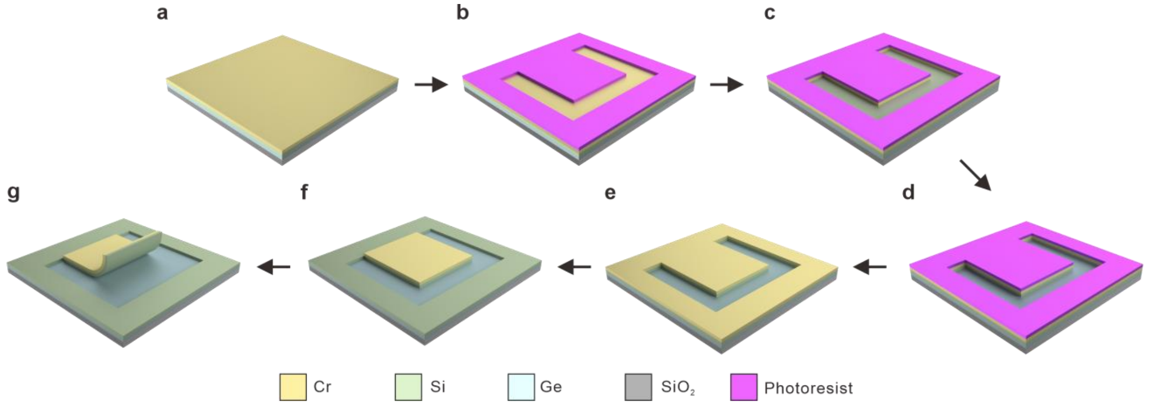

Supplementary Figure 23. Fabrication process of Si/Cr bilayer nanomembranes. a Ge/Si/Cr deposition on Si substrate. b Spin coating and define etching window via lithography. **c** Etching Cr layer of window by Cr etchant. **d** Etching excess Si layer by reactive ion etching. **e** Removing photoresist by acetone. **f** Remove redundant Cr layer by Cr etchant. **g** Releasing Si/Cr bilayer via etching Ge sacrificial layer.

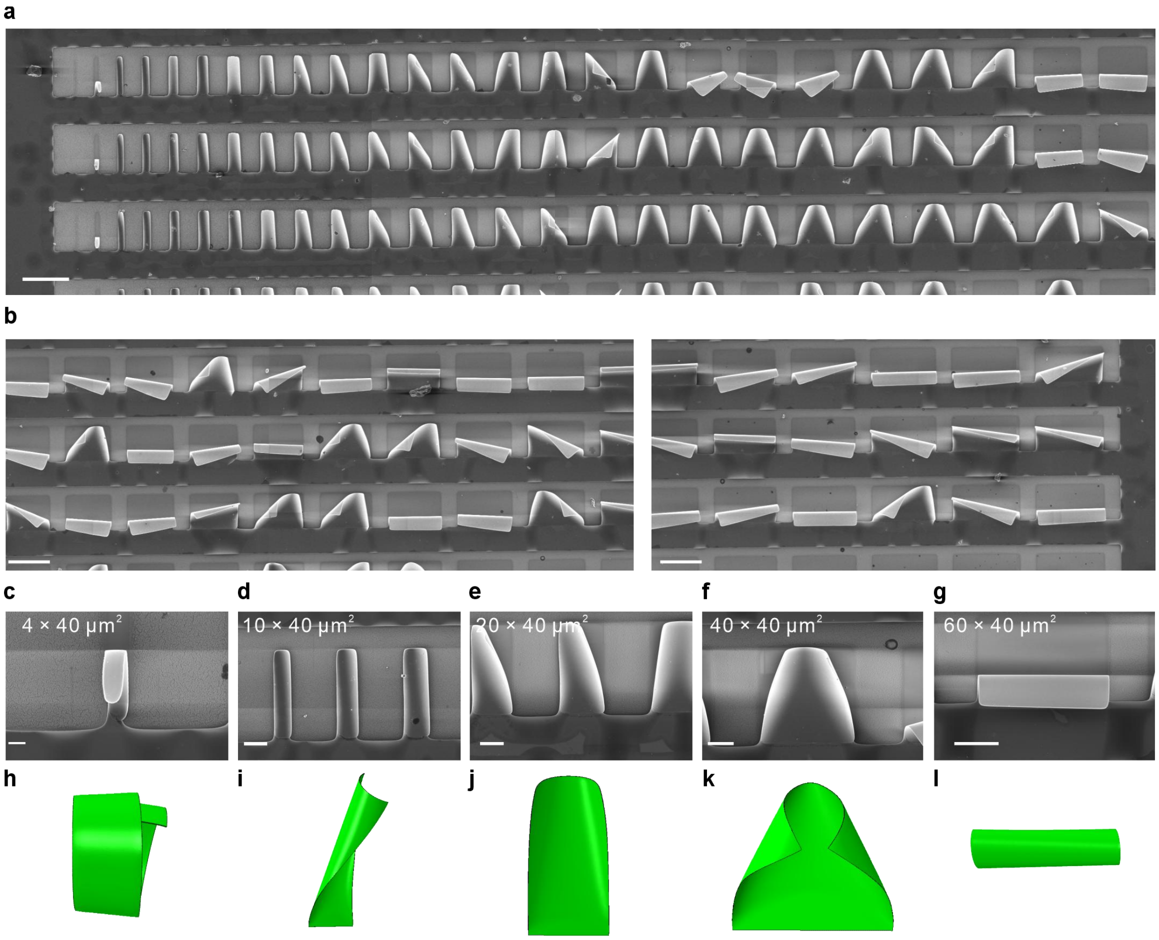

Supplementary Figure 24. SEM images and FEM results of Si (60 nm)/Cr (10 nm) double-layer rectangular nanomembrane (sample #1). a, b Panorama SEM images of self-assembled bilayer rectangular nanomembranes. Scale bars, 50 μm. SEM images of double-layer rectangular nanomembranes with different sizes of **c** 4×40 μm^2 (scale bar, 3 μm), **d** 10×40 μm^2 (scale bar, 10 μm), **e** 20×40 μm^2 (scale bar, 10 μm), **f** 40×40 μm^2 (scale bar, 10 μm), and **g** 60×40 μm^2 (scale bar, 20 μm). FEM simulation results of double-layer rectangular nanomembranes with different sizes of **h** 4×40 μm^2, **i** 10×40 μm^2, **j** 20×40 μm^2, **k** 40×40 μm^2, and **l** 60×40 μm^2

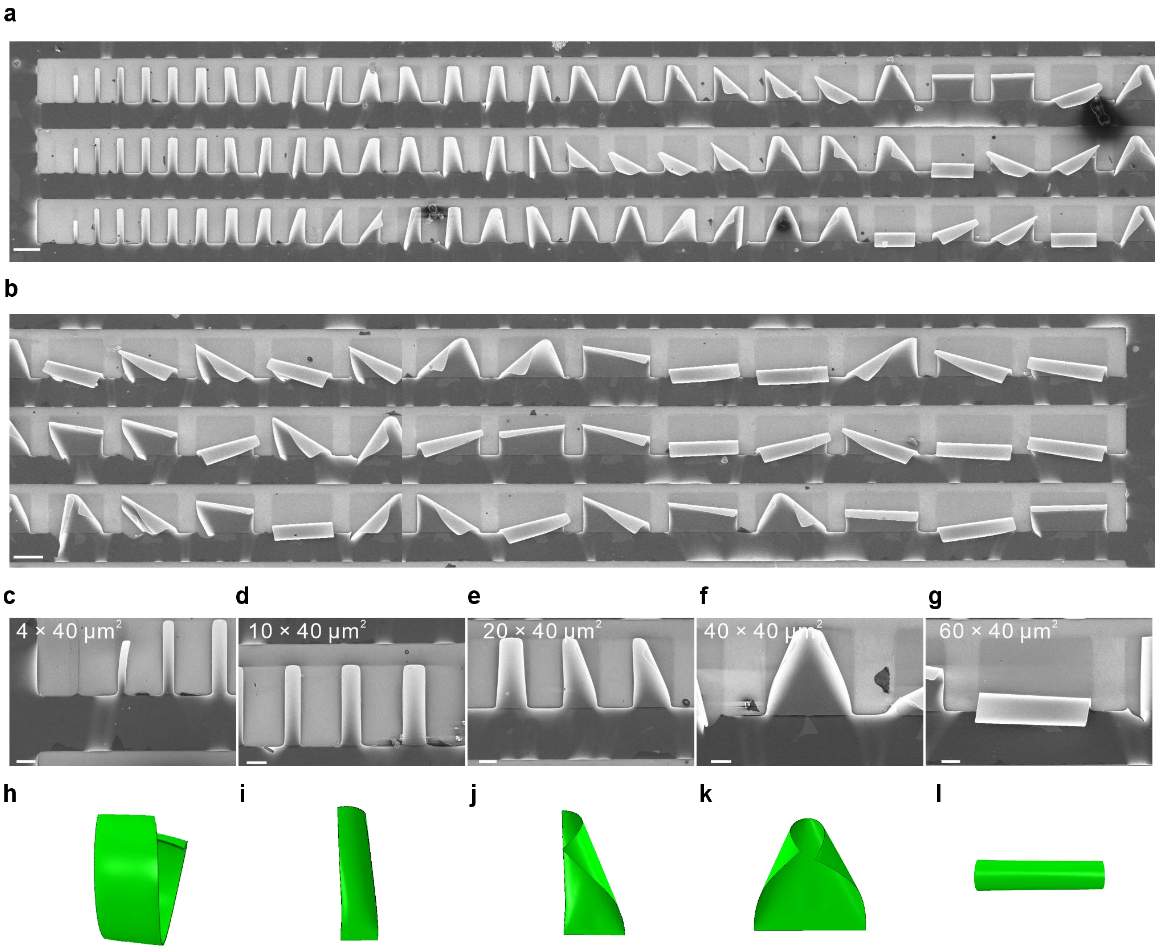

Supplementary Figure 25. SEM images and multilayer design model FEM results of Si (60 nm)/Cr (20 nm) double-layer rectangular nanomembrane (sample #2). a, b Panorama SEM images of self-assembled bilayer rectangular nanomembranes. SEM images of double-layer rectangular nanomembranes with different sizes of **c** 4×40 μm^2, **d** 10×40 μm^2, **e** 20×40 μm^2, **f** 40×40 μm^2, and **g** 60×40 μm^2. Scale bars, 10 μm. FEM simulation results of double-layer rectangular nanomembranes with different sizes of **h** 4×40 μm^2, **i** 10×40 μm^2, **j** 20×40 μm^2, **k** 40×40 μm^2, and **l** 60×40 μm^2

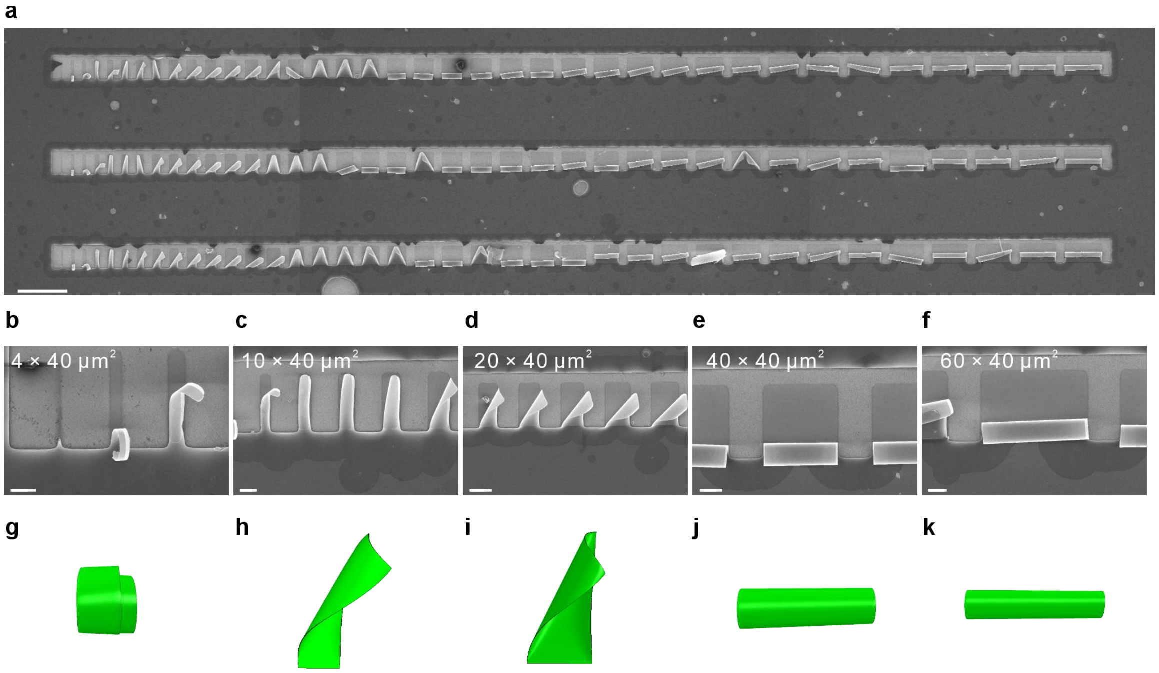

Supplementary Figure 26. SEM images and multilayer design model FEM results of Si (60 nm)/Cr (60 nm) double-layer rectangular nanomembrane (sample #3). a Panorama SEM images of self-assembled bilayer rectangular nanomembranes. Scale bar, 100 μm. SEM images of double-layer rectangular nanomembranes with different sizes of **b** 4×40 μm^2 (scale bar, 10 μm), **c** 10×40 μm^2 (scale bar, 10 μm), **d** 20×40 μm^2 (scale bar, 20 μm), **e** 40×40 μm^2 (scale bar, 10 μm), and **f** 60×40 μm^2 (scale bar, 10 μm). FEM simulation results of double-layer rectangular nanomembranes with different sizes of **g** 4×40 μm^2, **h** 10×40 μm^2, **i** 20×40 μm^2, **j** 40×40 μm^2, and **k** 60×40 μm^2.

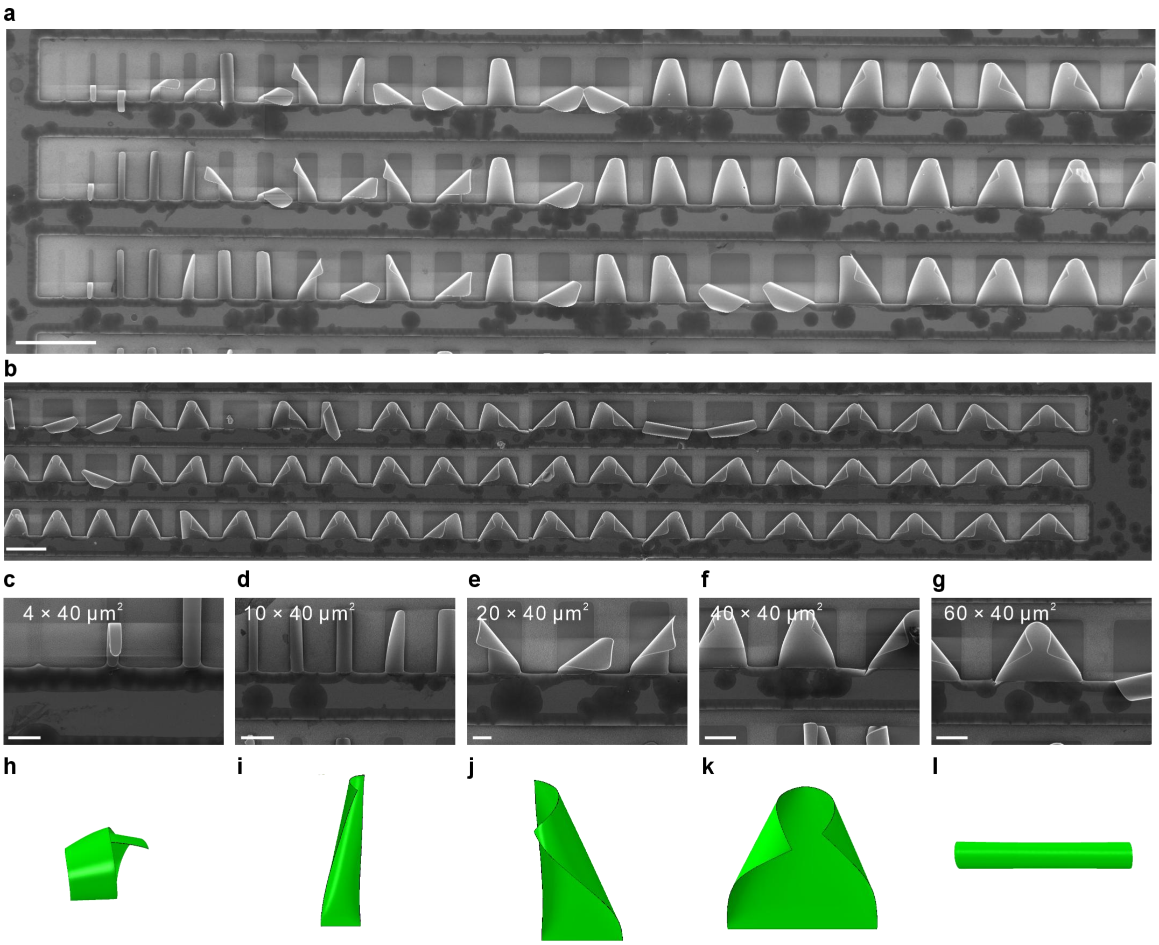

Supplementary Figure 27. SEM images and multilayer design model FEM results of Si (15 nm)/Cr (40 nm) double-layer rectangular nanomembrane (sample #4). a, b Panorama SEM images of self-assembled bilayer rectangular nanomembranes. Scale bars, 60 μm. SEM images of double-layer rectangular nanomembranes with different sizes of **c** 4×40 μm^2 (scale bar, 10 μm), **d** 10×40 μm^2 (scale bar, 20 μm), **e** 20×40 μm^2 (scale bar, 1 μm), **f** 40×40 μm^2 (scale bar, 20 μm), and **g** 60×40 μm^2 (scale bar, 20 μm). FEM simulation results of double-layer rectangular nanomembranes with different sizes of **h** 4×40 μm^2, **i** 10×40 μm^2, **j** 20×40 μm^2, **k** 40×40 μm^2, and **l** 60×40 μm^2

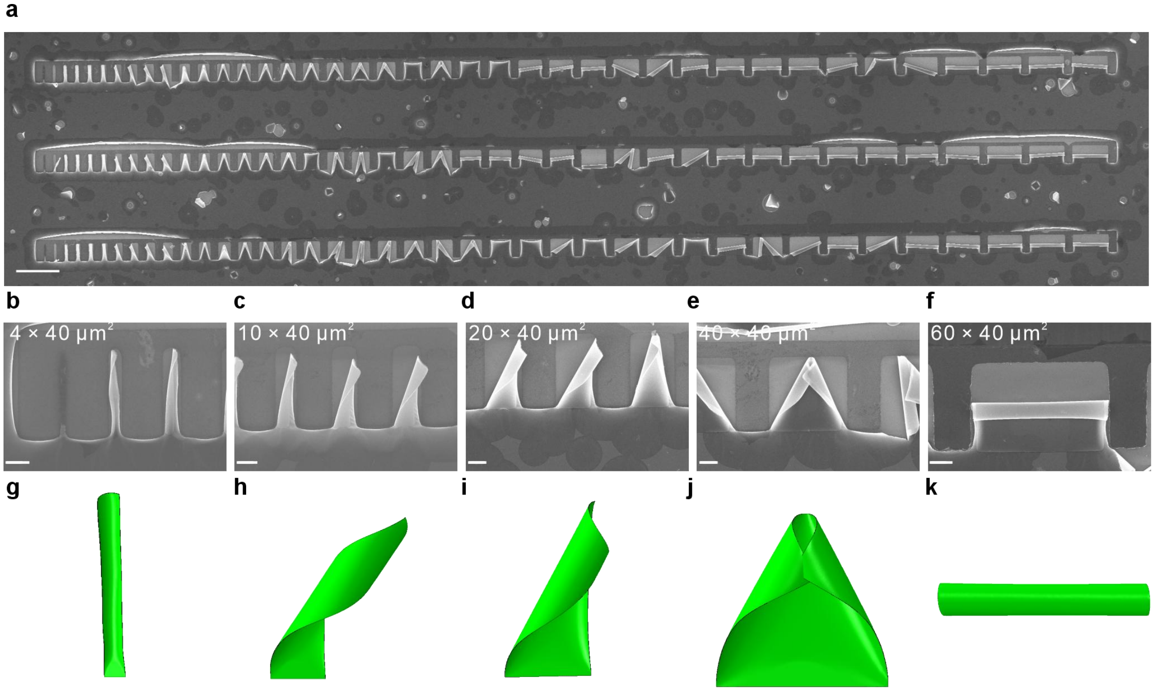

Supplementary Figure 28. SEM images and multilayer design model FEM results of Si (30 nm)/Cr (40 nm) double-layer rectangular nanomembrane (sample #5). a Panorama SEM images of self-assembled bilayer rectangular nanomembranes. Scale bar, 100 μm. SEM images of double-layer rectangular nanomembranes with different sizes of **b** 4×40 μm^2, **c** 10×40 μm^2, **d** 20×40 μm^2, **e** 40×40 μm^2, and **f** 60×40 μm^2. Scale bars, 10 μm. FEM simulation results of double-layer rectangular nanomembranes with different sizes of **g** 4×40 μm^2, **h** 10×40 μm^2, **i** 20×40 μm^2, **j** 40×40 μm^2, **k** 60×40 μm^2.

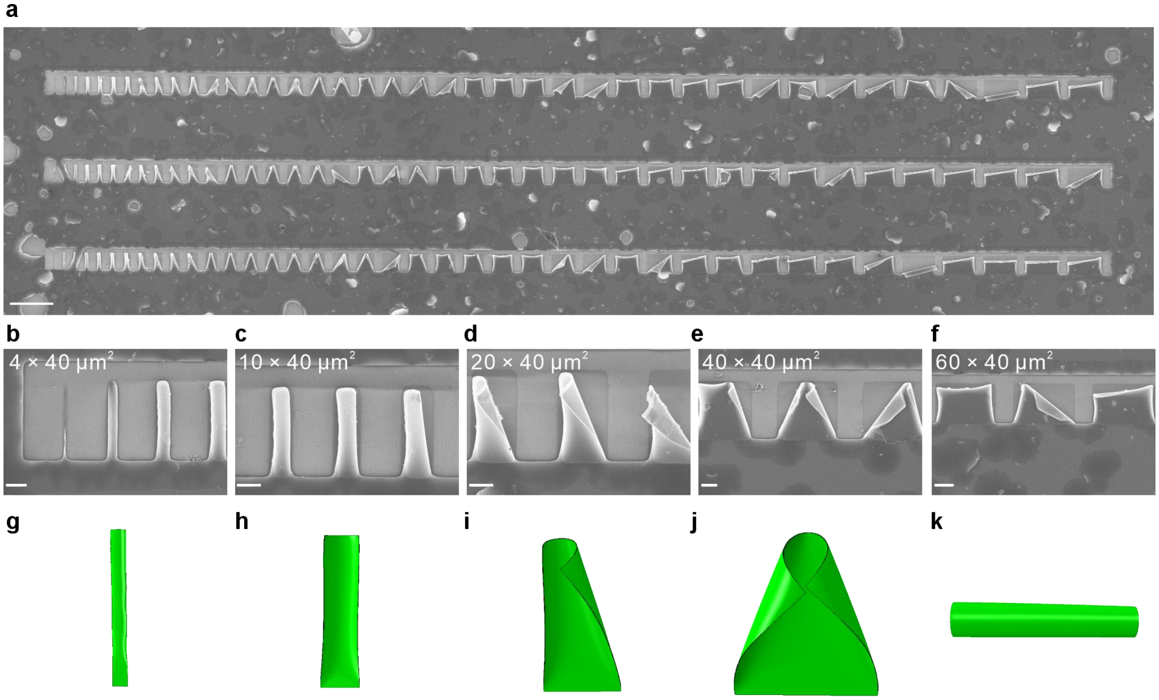

Supplementary Figure 29. SEM images and multilayer design model FEM results of Si (90 nm)/Cr (40 nm) double-layer rectangular nanomembrane (sample #6). a, b Panorama SEM images of self-assembled bilayer rectangular nanomembranes. Scale bar, 100 μm. SEM images of double-layer rectangular nanomembranes with different sizes of **b** 4×40 μm^2 (scale bar, 10 μm), **c** 10×40 μm^2 (scale bar, 10 μm), **d** 20×40 μm^2 (scale bar, 10 μm), **e** 40×40 μm^2, (scale bar, 10 μm) and **f** 60×40 μm^2 (scale bar, 20 μm). FEM simulation results of double-layer rectangular nanomembranes with different sizes of **g** 4×40 μm^2, **h** 10×40 μm^2, **i** 20×40 μm^2, **j** 40×40 μm^2, **k** 60×40 μm^2.

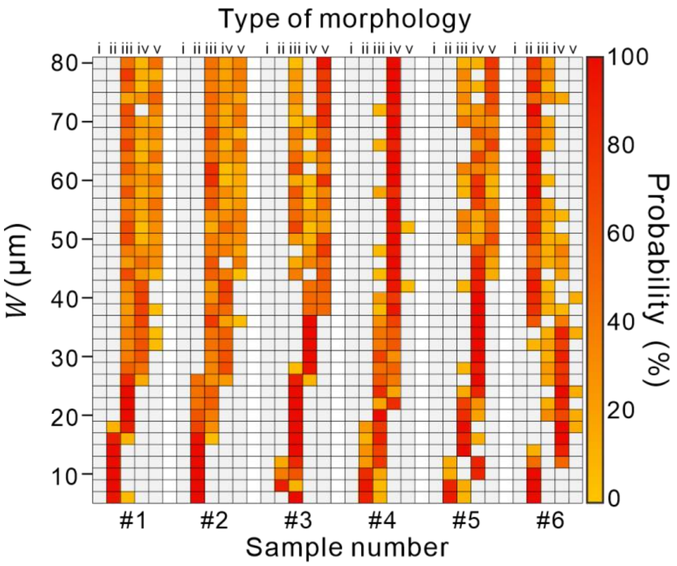

Supplementary Figure 30. Relationship between structures and widths of the samples.

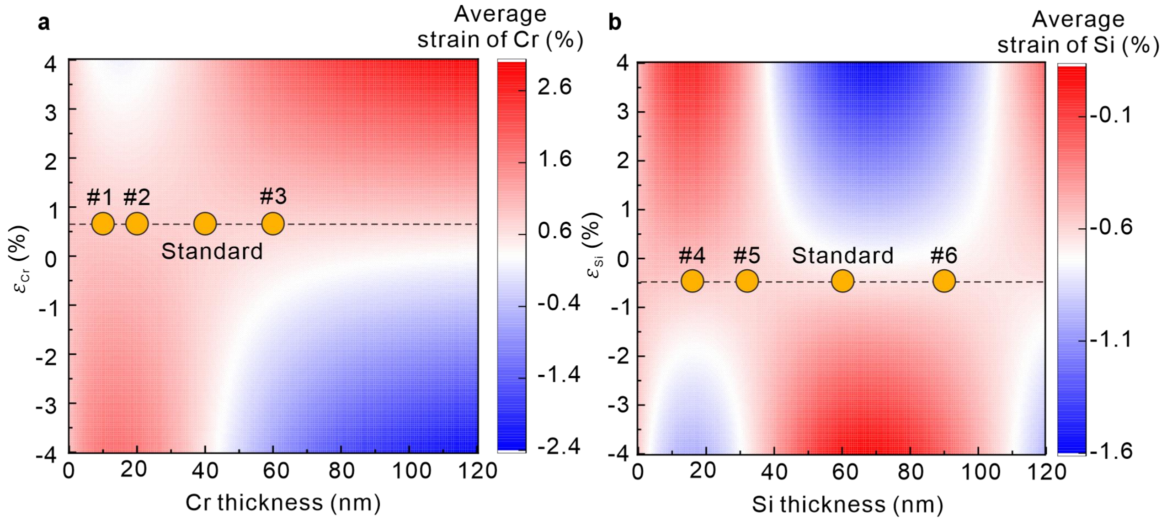

Supplementary Figure 31. Strain distribution of Si/Cr self-rolling microtubes under different nanomembrane thicknesses. a Dependence of the average strain of the Cr layer on the pre-strain of the Cr layer and the thickness of the Cr layer in Si/Cr microtubes. **b** Dependence of the average strain of the Si layer on the pre-strain of the Si layer and the thickness of the Si layer in Si/Cr microtubes.

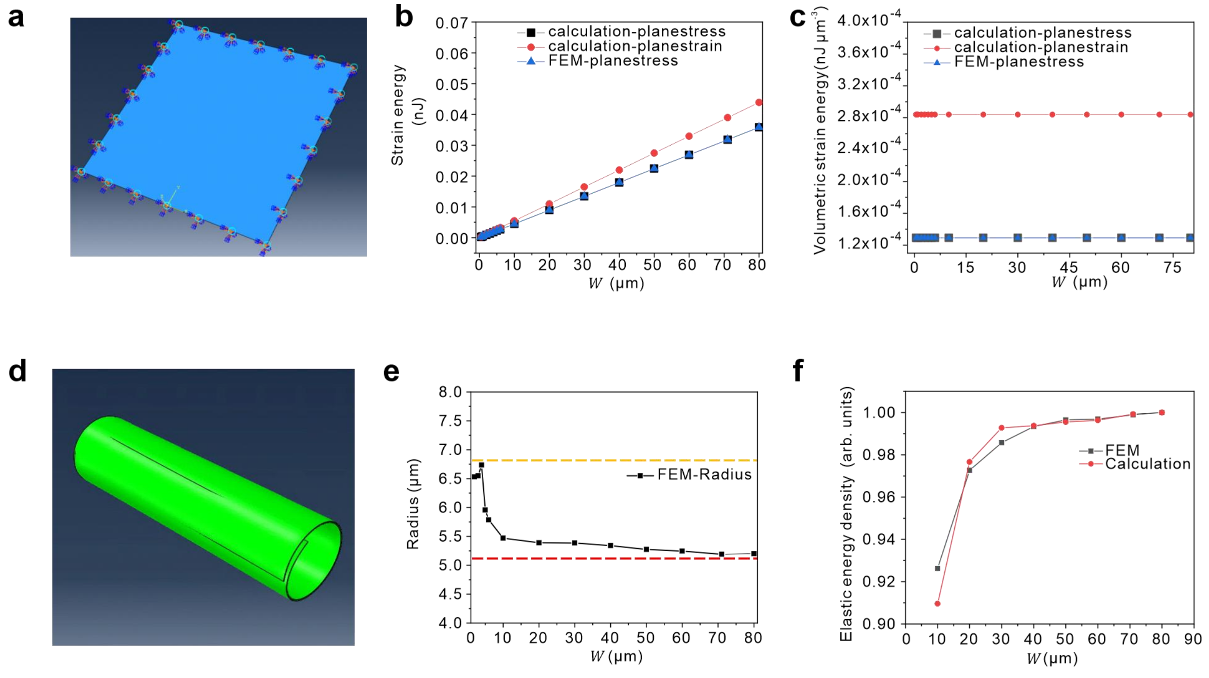

Supplementary Figure 32. Strain energy calculation model in pre-strained bilayer and unidirectional rolling tube. a Illustration of pre-strained bilayer nanomembrane. **b** Total elastic energy of pre-strained bilayer in FEM and analytical calculation. **c** Elastic energy density of pre-strained bilayer in FEM and analytical calculation. **d** Schematic diagram of unidirectional rolling tube. **e** Total elastic energy and **f** elastic energy density of unidirectional rolling tube in FEM and analytical calculation.

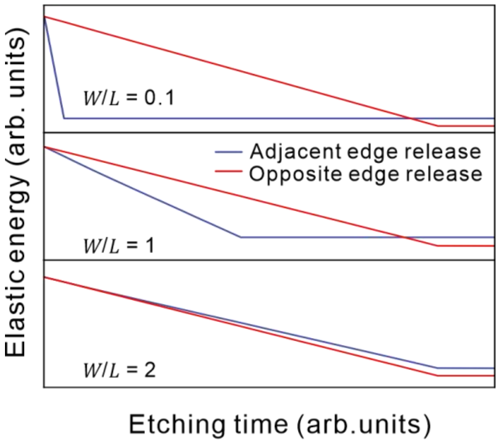

Supplementary Figure 33. Elastic energy of nanomembranes releasing from different direction. Elastic energy of nanomembranes releasing from adjacent edge and opposite edge for W/L = 0.1, 1, and 2.

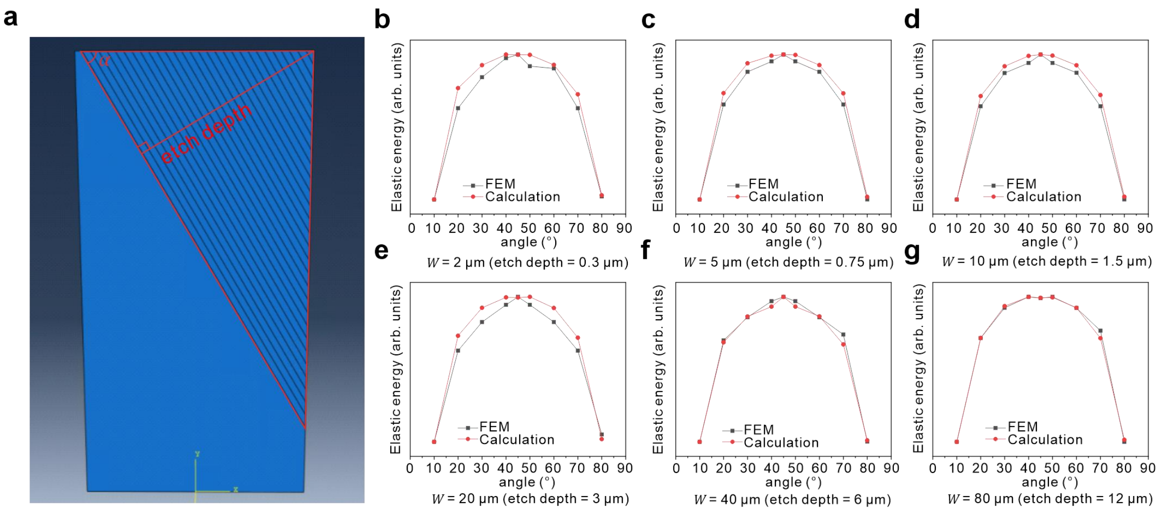

Supplementary Figure 34. Verification of strain energy calculation in unilateral-rolling in released triangle region. a model of single-side angular rolling. Total elastic energy of single-side angular rolling model in FEM and analytical calculation in **b** W = 2 μm, **c** W = 5 μm, **d** W = 10 μm, **e** W = 20 μm, **f** W = 40 μm, **g** W = 80 μm.

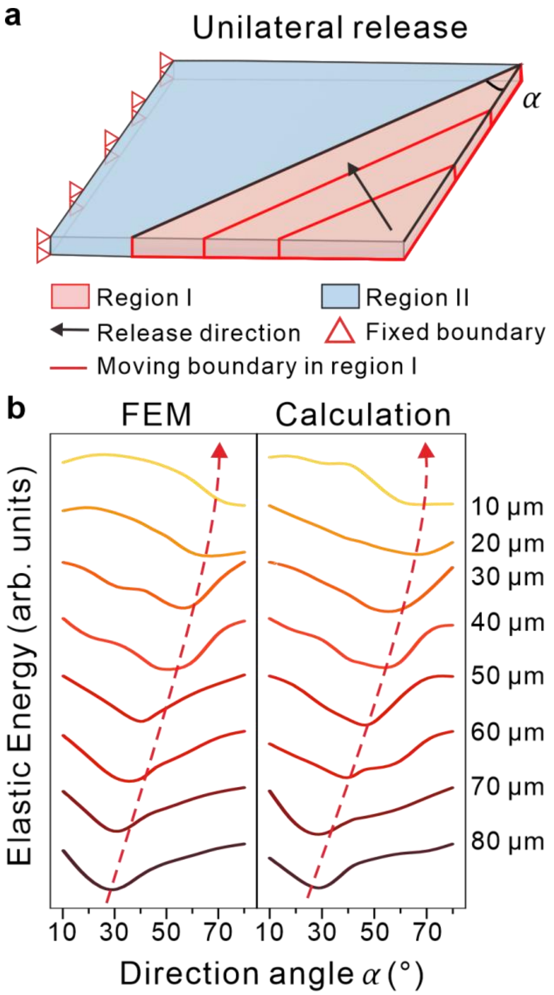

Supplementary Figure 35. Schematic diagram and elastic energy of unilateral release model. a Diagram of idealized unilateral release models that consist two regions released in chronological order. **b** Relative elastic energy of idealized unilateral release models with different direction angles and pattern widths via FEM simulation.

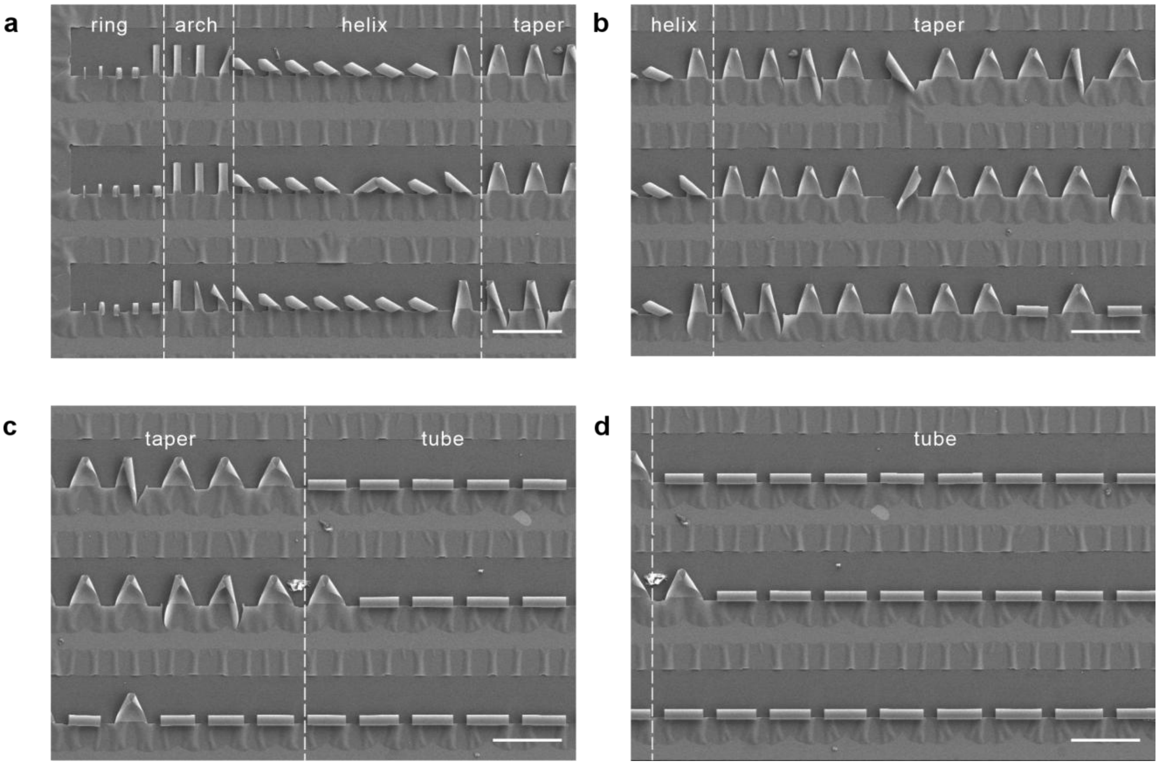

Supplementary Figure 36. SEM images for transition of Si/Cr bilayer in different size (L = 45 µm). a Morphology transition between ring, arch, helix, and taper structure. **b** Morphology transition between helix and taper structure. **c** Morphology transition between taper and tube structure. **d** Morphology of tube structure with a large pattern width (W > 40 µm). Scale bars, 100 µm.

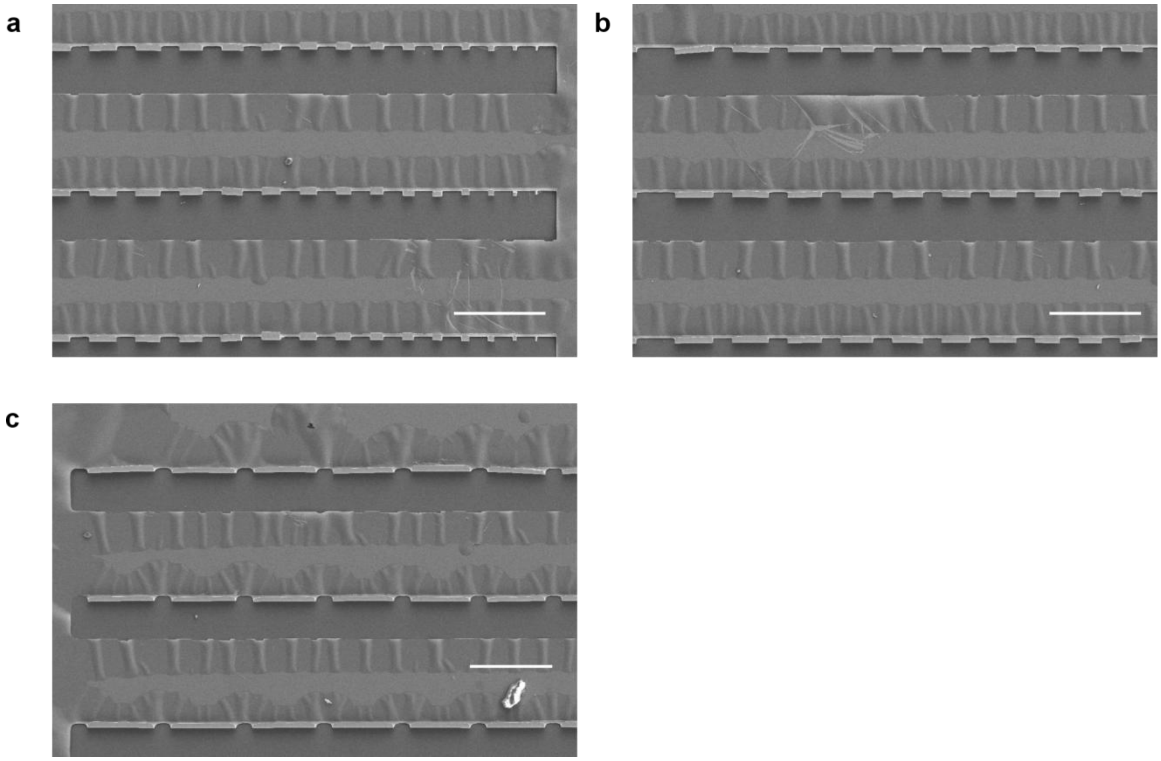

Supplementary Figure 37. SEM images for transition of Si/Cr bilayer in different size (*L* = 20 μm)**.** **a** SEM image of Si/Cr bilayer from *W* = 2 μm to *W* = 30 μm. **b** SEM image of Si/Cr bilayer from *W* = 22 μm to *W* = 40 μm. **c** SEM image of Si/Cr bilayer from *W* = 70 μm to *W* = 80 μm. Scale bars, 100 μm.

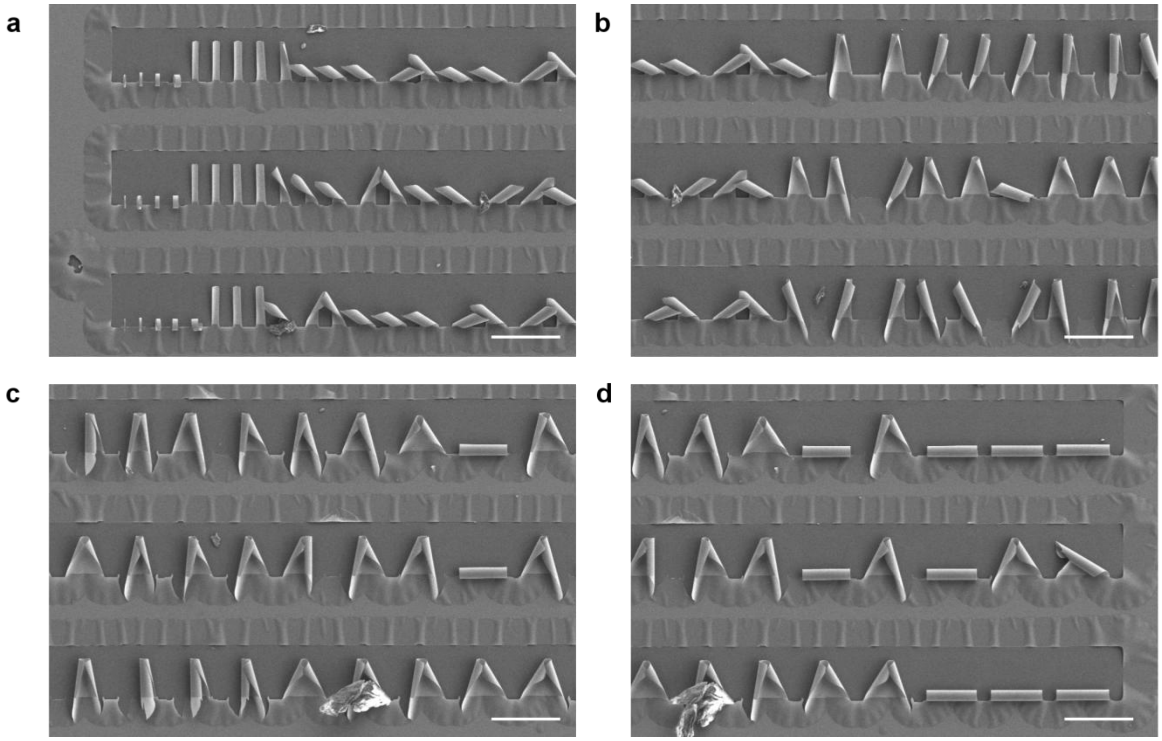

Supplementary Figure 38. SEM images for transition of Si/Cr bilayer in different size (L = 60 μm)**. a** SEM image of Si/Cr bilayer from W = 2 μm to W = 36 μm. **b** SEM image of Si/Cr bilayer from W = 32 μm to W = 56 μm. **c** SEM image of Si/Cr bilayer from W = 58 μm to W = 74 μm. **d** SEM image of Si/Cr bilayer from W = 66 μm to W = 80 μm. Scale bars, 100 μm.

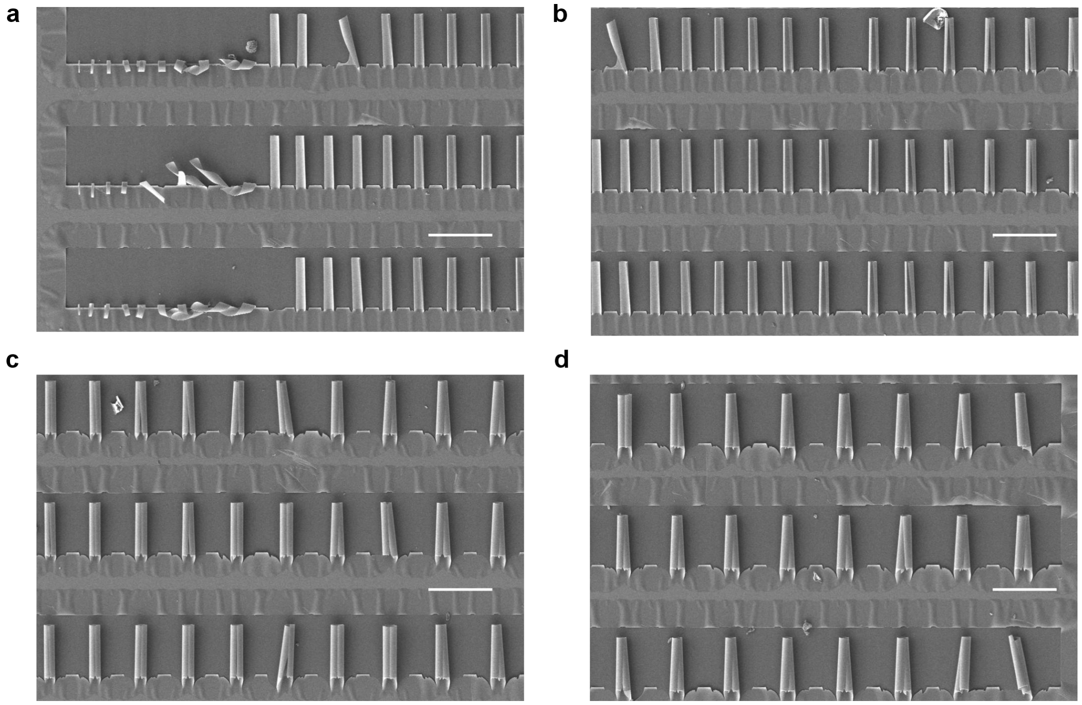

Supplementary Figure 39. SEM images for transition of Si/Cr bilayer in different size (L = 80 μm). **a** SEM image of Si/Cr bilayer from W = 2 μm to W = 36 μm. **b** SEM image of Si/Cr bilayer from W = 26 μm to W = 50 μm. **c** SEM image of Si/Cr bilayer from W = 46 μm to W = 64 μm. **d** SEM image of Si/Cr bilayer from W = 66 μm to W = 80 μm.Scale bars, 100 μm.

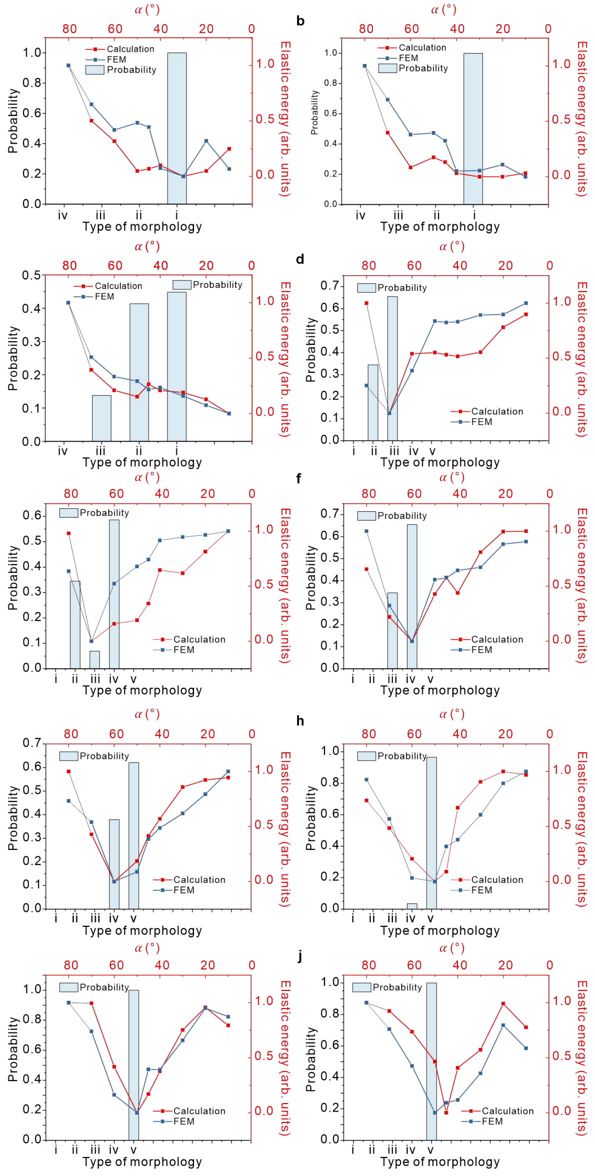

Supplementary Figure 40. Relationship between probability of morphological in different pattern and changes in corresponding elastic energy density of Si/Cr bilayer. Statistical graph of pattern with L = 40 μm, **a** W = 4 μm, **b** W = 8 μm, **c** W = 10 μm, **d** W = 20 μm, **e** W = 30 μm, **f** W = 40 μm, **g** W = 50 μm, **h** W = 60 μm, **i** W = 70 μm, and **j** W = 80 μm.

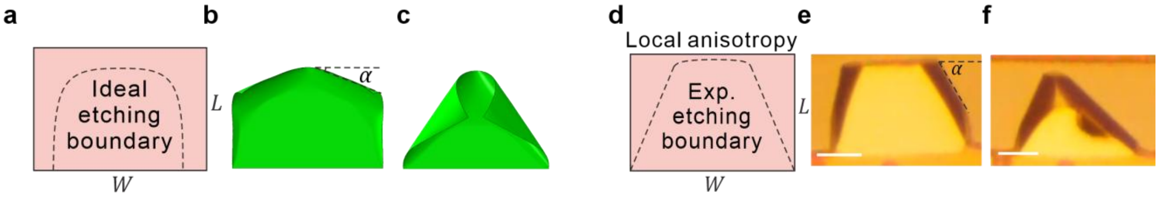

Supplementary Figure 41. Non-ideal situations in FEM and experiments. a-c Ideal etching boundary as envisioned in the FEM model. **d-f** The local anisotropic release process and optical images exist in the experiment. Scale bars, 20 μm.

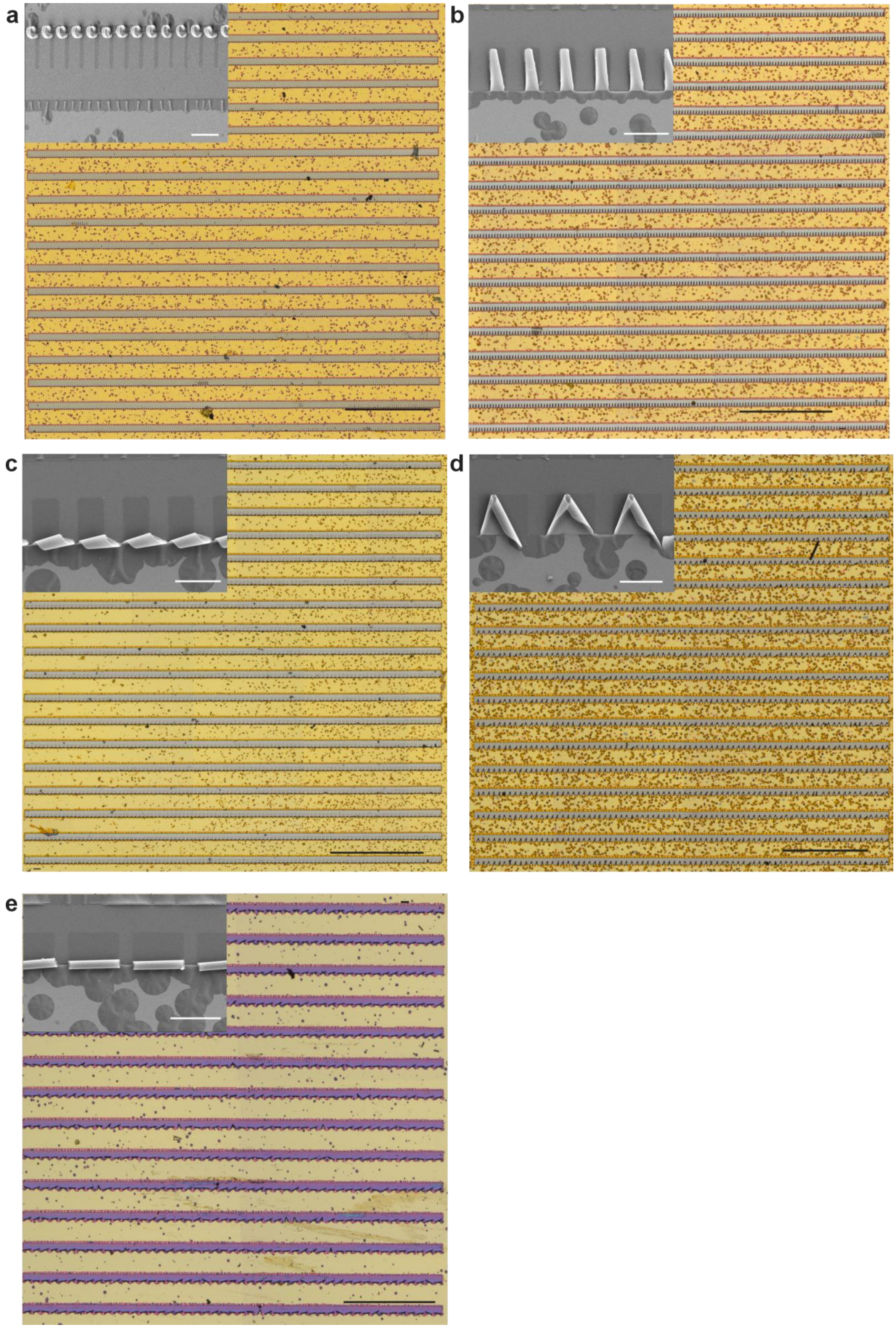

1345

Supplementary Figure 42. Images of microstructure arrays. Optical images and SEM images (inset) of **a** ring (scale bar, 40 μm), **b** arch (scale bar, 40 μm), **c** helix (scale bar, 40 μm), **d** taper (scale bar, 40 μm), and **e** tube structures (scale bar, 60 μm). Scale bars of optical images, 1 mm.

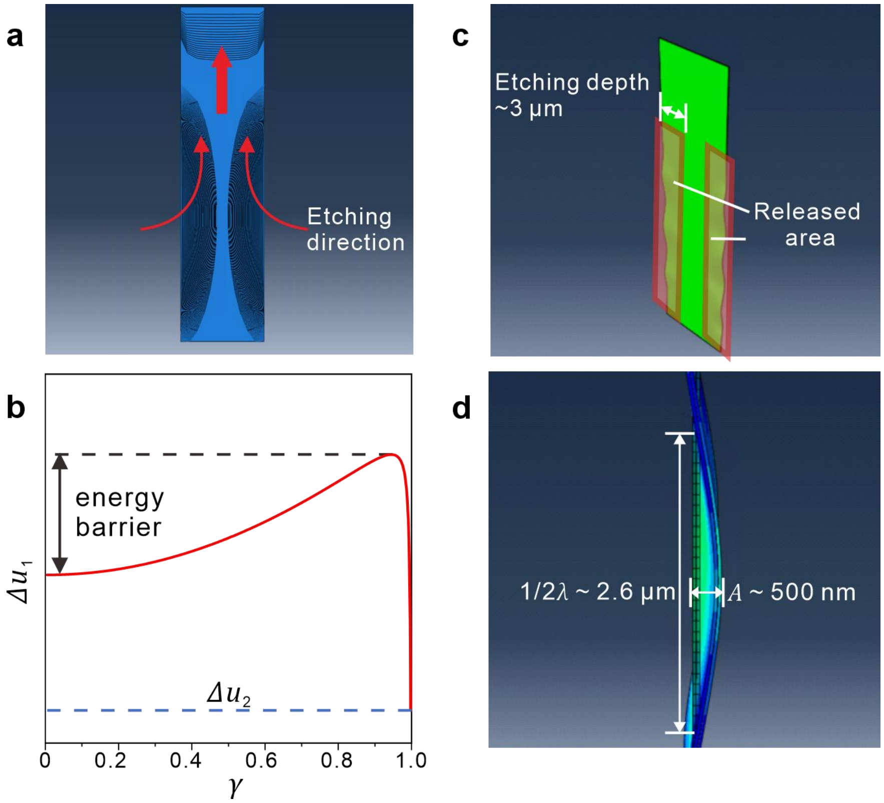

Supplementary Figure 43. Multilevel model in bottom etching. **a** quasistatic mechanical FEM model in fixed end etching (L = 40 μm, W = 10 μm). **b** Relative elastic energy between rolling from vertical direction and rolling from wrinkle direction. **c** Wrinkles of Si/Cr bilayer in incomplete etching process in FEM. **d** Amplitude and half wavelength of wrinkles.

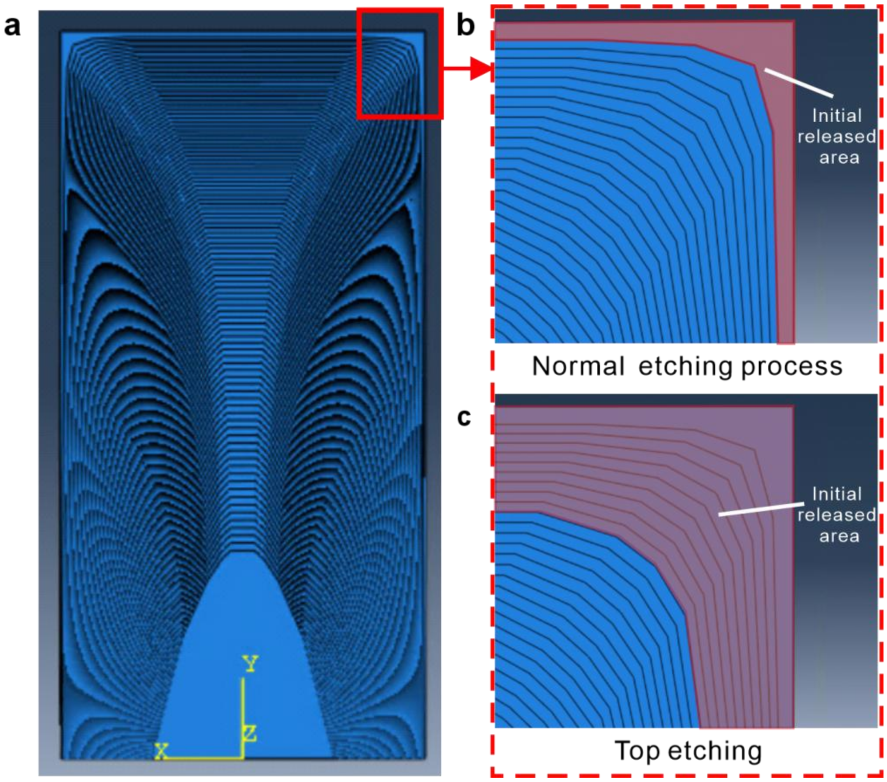

Supplementary Figure 44. Multilevel model in top etching. a Quasistatic mechanical FEM model (L = 40 μm, W = 20 μm). Initial released area in **b** normal etching process and **c** top etching process.

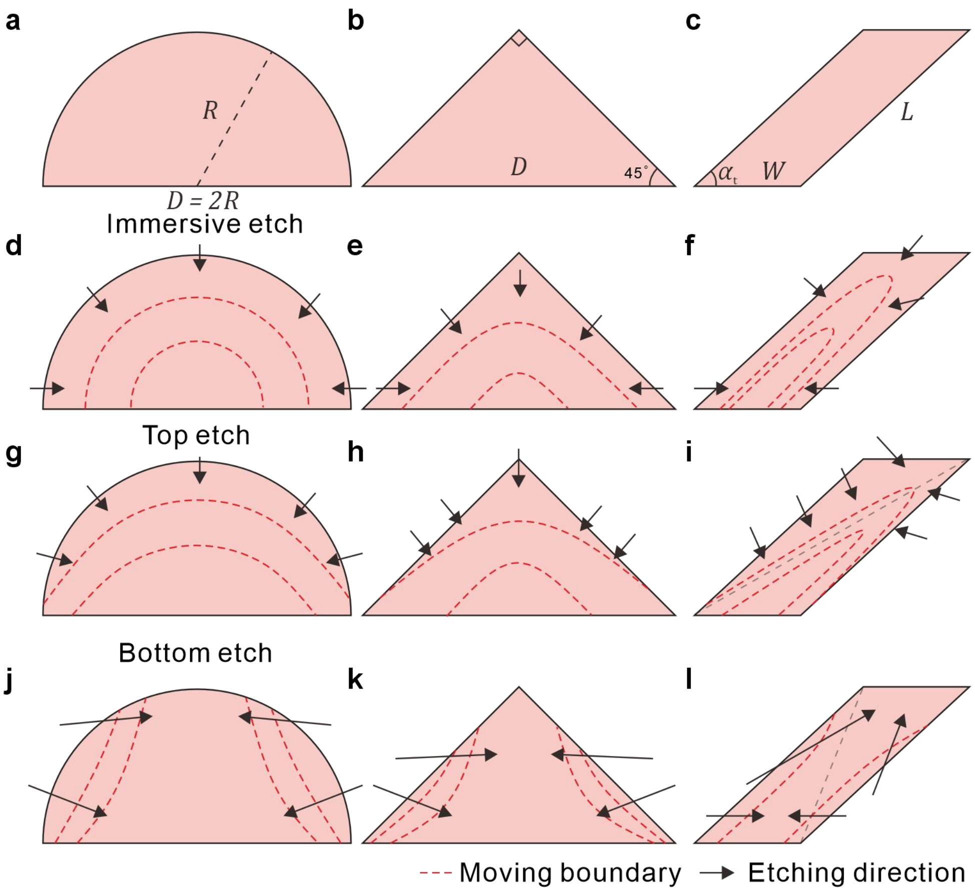

Supplementary Figure 45. Schematic diagram of the etching process of different shapes. The red dotted line in the figure is the moving boundary of the sacrificial layer, and the black arrow is the etching direction. Geometric parameters of **a** semicircle, **b** isosceles right triangle, **c** parallelogram strip. Schematic diagram of the sacrificial layer boundary changes of **d** semicircle, **e** isosceles right triangle, and **f** parallelogram strip that are completely immersed in the etchant. Schematic diagram of the boundary changes of the sacrificial layer during top etching of **g** semicircle, **h** isosceles right triangle, and **i** parallelogram strip. Schematic diagram of the boundary changes of the sacrificial layer during bottom etching of **j** semicircle, **k** isosceles right triangle, and **l** parallelogram strip.

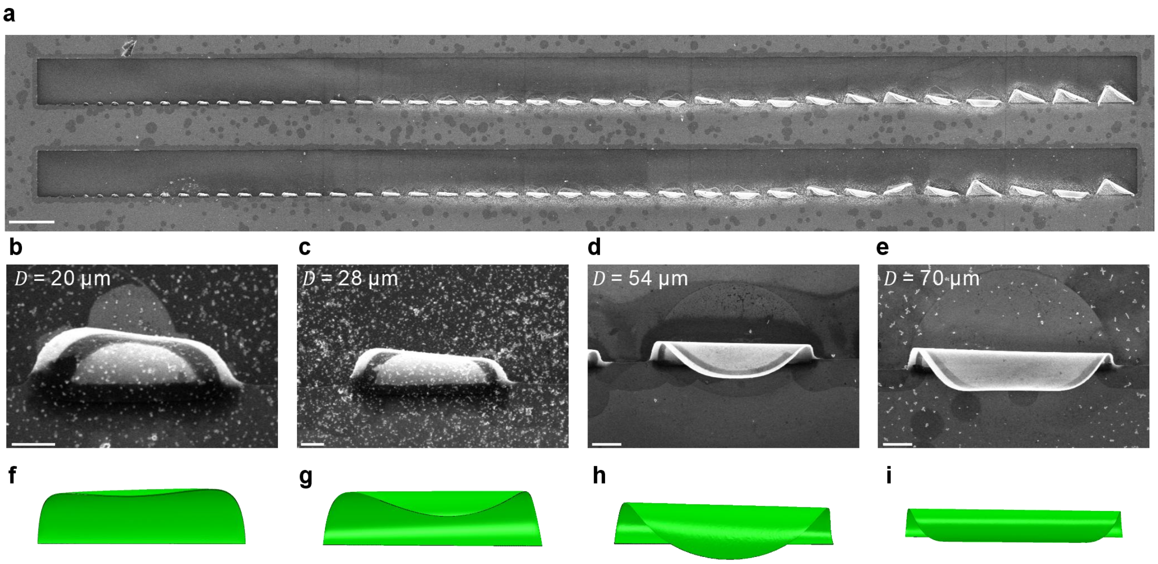

Supplementary Figure 46. SEM images and FEM results of the bottom-etched self-assembled semicircle of the multilevel design model. a Panorama SEM images of the bottom-etched self-assembled semicircle. Scale bars, 100 μm. SEM images of bottom-etched self-assembled semicircles with sizes of **b** *D* = 20 μm (scale bar, 4 μm), **c** *D* = 28 μm (scale bar, 4 μm), **d** *D* = 54 μm (scale bar, 10 μm), and **e** *D* = 70 μm (scale bar, 10 μm). TEM results of bottom-etched self-assembled semicircles with sizes of **f** *D* = 20 μm, **g** *D* = 28 μm, **h** *D* = 54 μm, and **i** *D* = 70 μm.

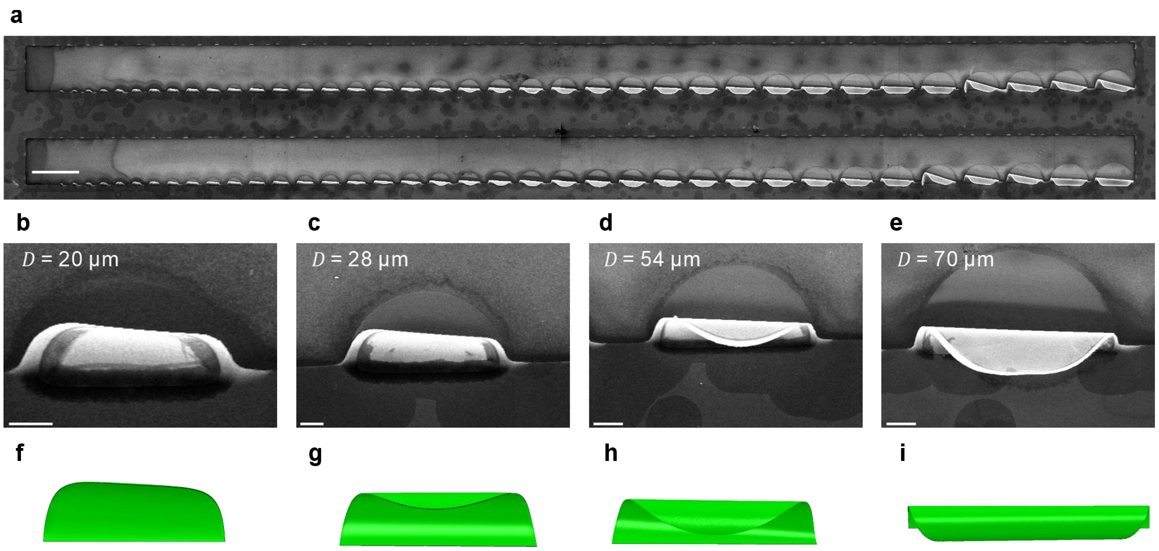

Supplementary Figure 47. SEM images and FEM results of the top-etched self-assembled semicircle of the multilevel design model. a Panorama SEM images of the top-etched self-assembled semicircle. SEM images of top-etched self-assembled

semicircles with sizes of **b** D = 20 μm (scale bar, 4 μm), **c** D = 28 μm (scale bar, 4 μm), **d** D = 54 μm (scale bar, 10 μm), and **e** D = 70 μm (scale bar, 10 μm). FEM results of top-etched self-assembled semicircles with sizes of **f** D = 20 μm, **g** D = 28 μm, **h** D = 54 μm, and **i** D = 70 μm.

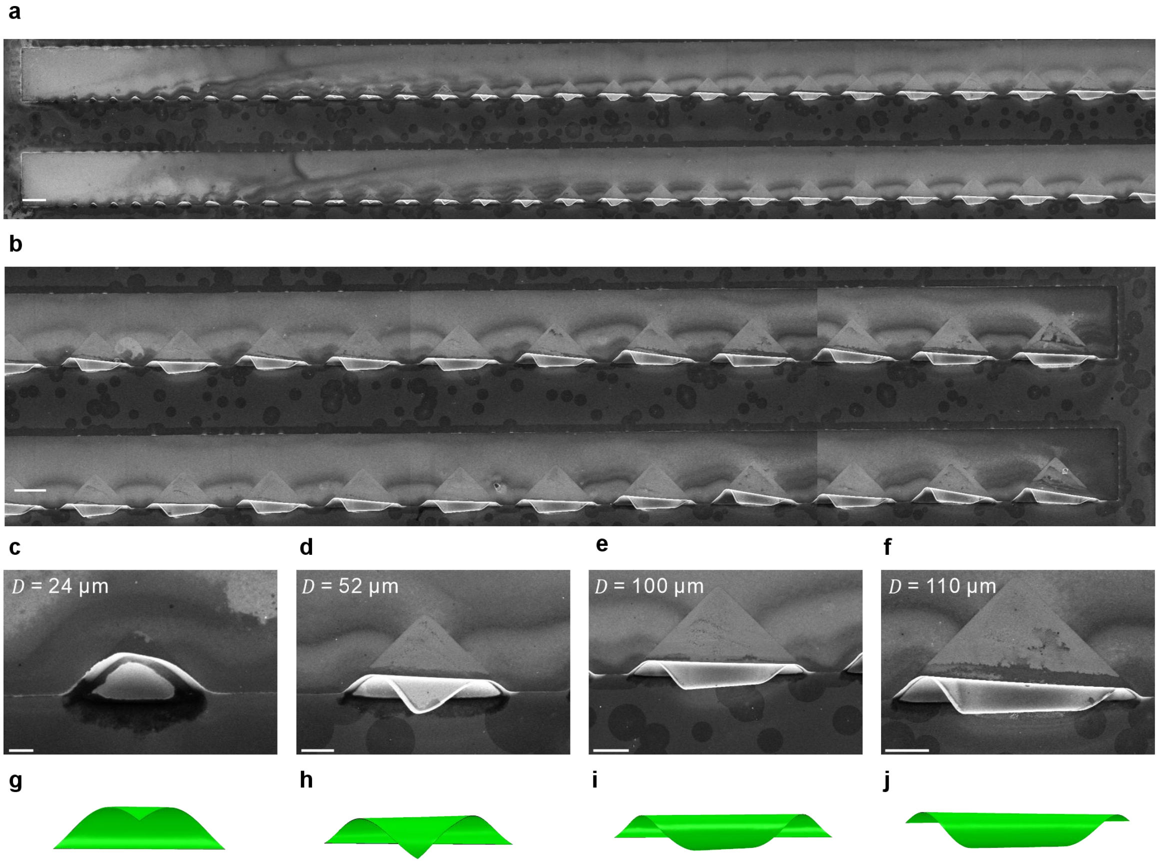

Supplementary Figure 48. SEM images and FEM results of the bottom-etched self-assembled triangle. a, b Panorama SEM images of bottom-etched self-assembled triangles. Scale bars, 40 μm. SEM images of bottom-etched self-assembled triangles with sizes of **c** D = 24 μm (scale bar, 4 μm), **d** D = 52 μm (scale bar, 10 μm), **e** D = 100 μm (scale bar, 20 μm), and **f** D = 110 μm (scale bar, 20 μm). FEM simulation results of the bottom-etched triangle with sizes of **g** D = 24 μm, **h** D = 52 μm, **i** D = 100 μm, and **j** D = 110 μm.

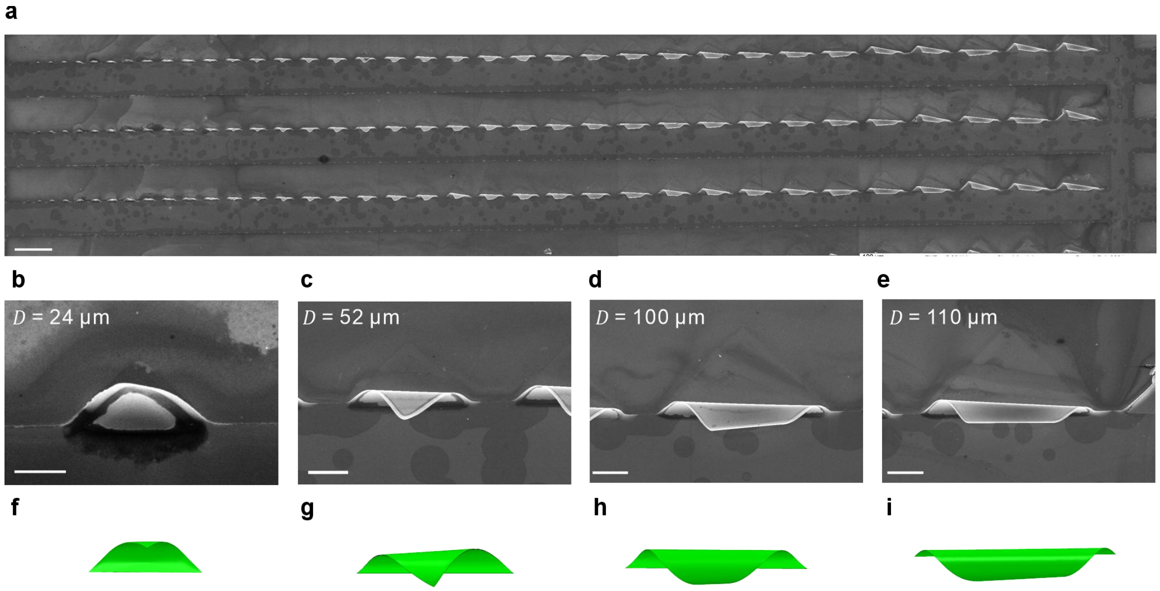

Supplementary Figure 49. SEM images and FEM results of the top-etched self-assembled triangle. **a** Panorama SEM images of the top-etched self-assembled triangle. Scale bar, 100 μm. SEM images of top-etched self-assembled triangles with sizes of **b** *D* = 24 μm (scale bar, 10 μm), **c** *D* = 52 μm (scale bar, 10 μm), **d** *D* = 100 μm (scale bar, 20 μm), and **e** *D* = 110 μm (scale bar, 20 μm). FEM simulation results of the top-etched triangle with sizes of **f** *D* = 24 μm, **g** *D* = 52 μm, **h** *D* = 100 μm, and **i** *D* = 110 μm.

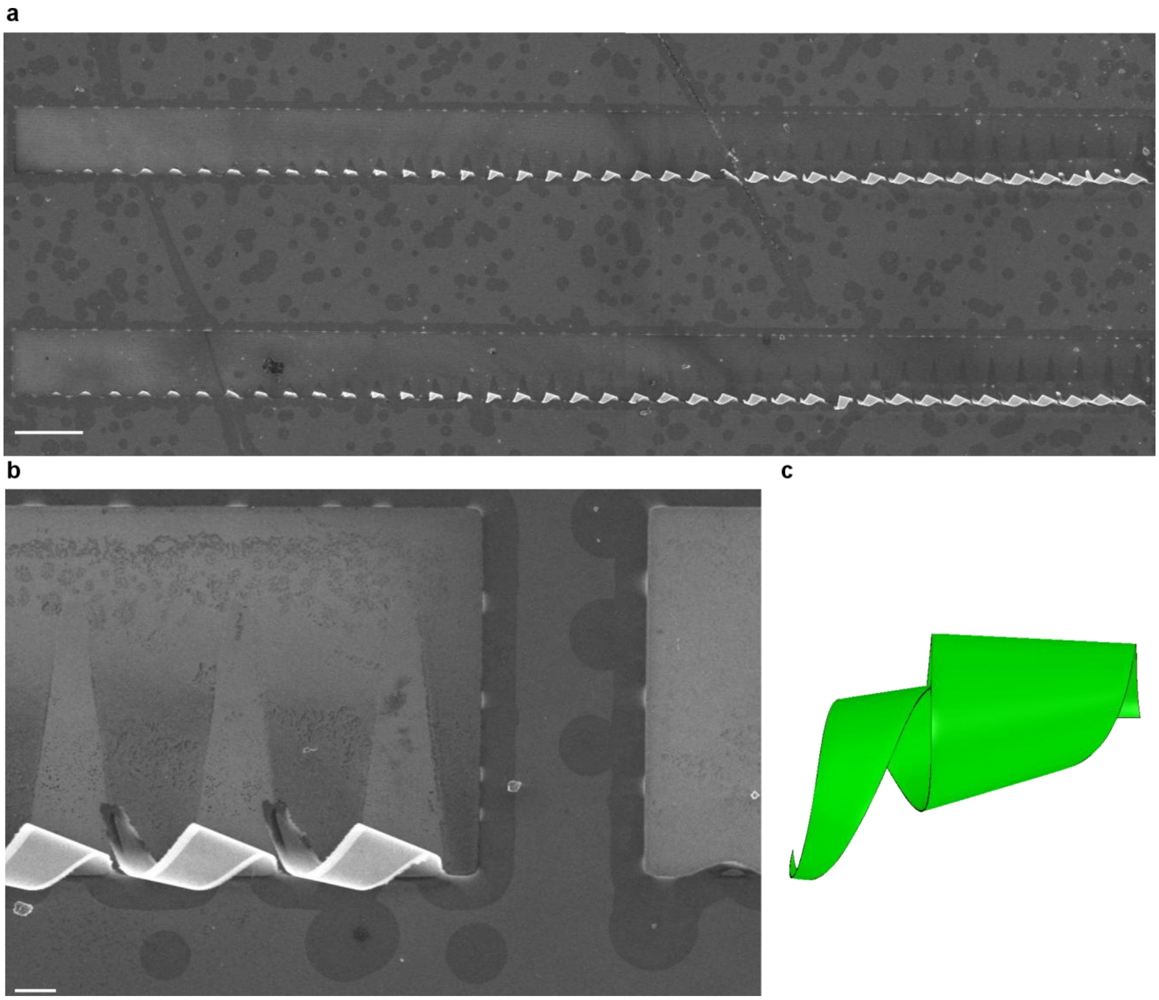

Supplementary Figure 50. SEM images and FEM results of immersive-etched self-assembled triangles of different heights. a Panorama SEM images of immersive-etched self-assembled triangles of different heights. Scale bar, 100 μm. **b** SEM images and **c** FEM simulation results of immersive-etched self-assembled triangles with D = 20 μm and triangle bottom height H = 80 μm. Scale bar, 10 μm.

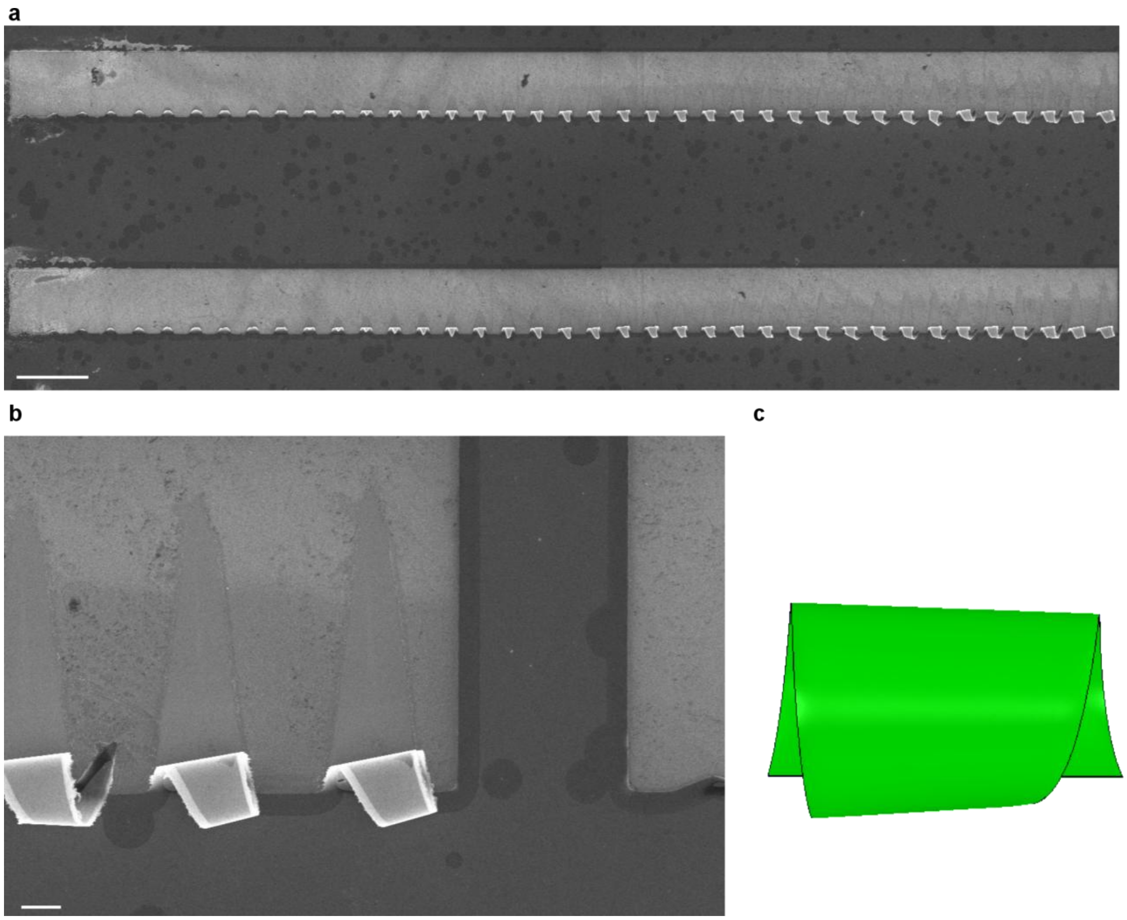

Supplementary Figure 51. SEM images and FEM results of bottom-etched self-assembled triangles with different heights. a Panorama SEM images of self-assembled triangles with different heights etched on the bottom. Scale bar, 100 μm. **b** SEM images and **c** FEM simulation results of bottom-etched self-assembled triangles with D = 20 μm and H = 80 μm. Scale bar, 10 μm.

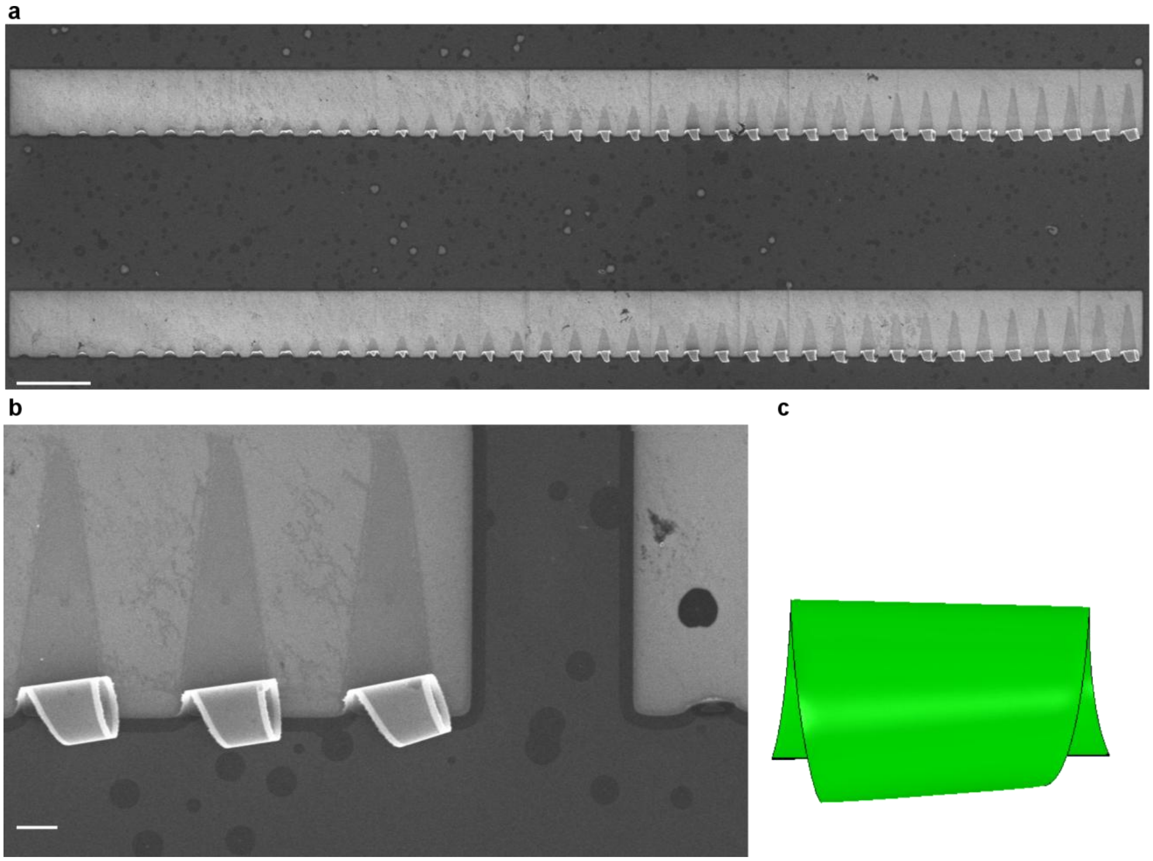

Supplementary Figure 52. SEM images of self-assembled triangles with different heights etched on the top and FEM results of the multilevel design model. a Panorama SEM images of self-assembled triangles with different heights etched on the top. Scale bars, 100 μm. **b** SEM images and **c** FEM simulation results of top-etched self-assembled triangles with D = 20 μm and H = 80 μm. Scale bars, 10 μm.

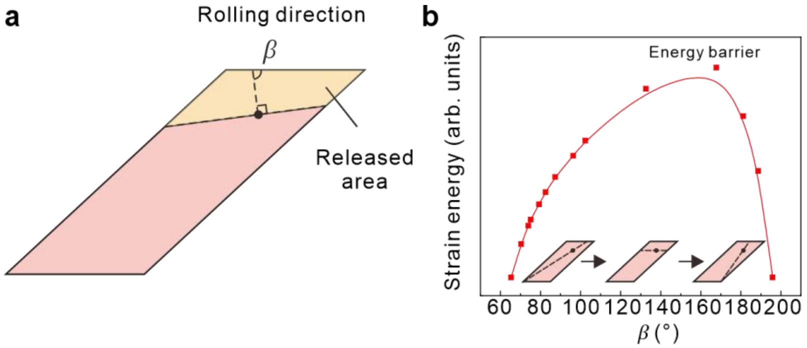

Supplementary Figure 53. Schematic diagram of the identification of multi-stable states during the etching process. a Schematic diagram of the etching direction in a parallelogram. **b** The strain energy-etching direction relationship diagram shows a strain energy barrier between the two lowest energy points.

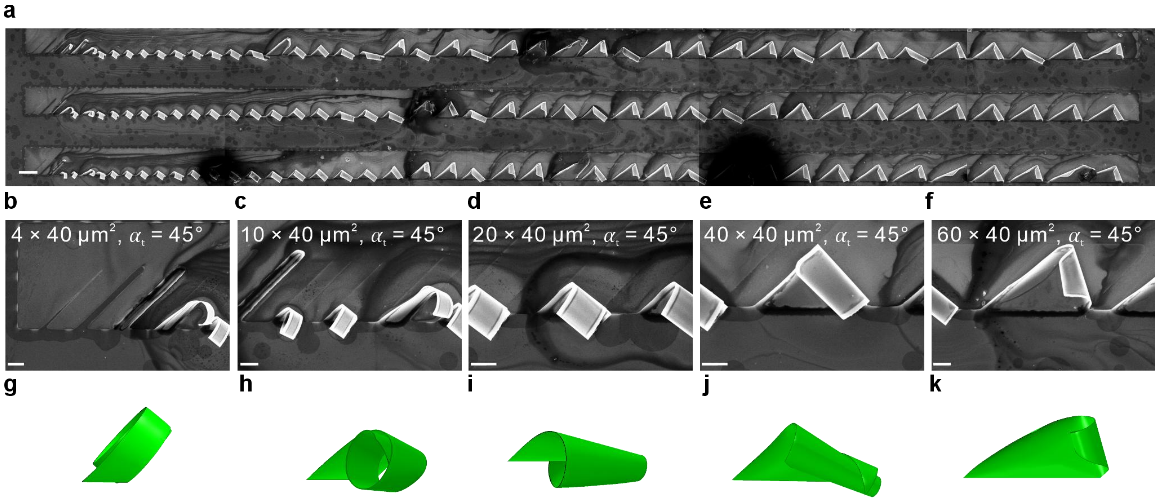

Supplementary Figure 54. SEM images and FEM results of immersive-etched parallelogram strips with different widths. a Panorama SEM images of immersive-etched self-assembled parallelogram strip nanomembranes with different widths. Scale bar, 40 μm. SEM images of self-assembled parallelogram strip immersive-etched nanomembrane with sizes of **b** 4×40 μm^2, α_t = 45°, **c** 10×40 μm^2, α_t = 45°, **d** 20×40 μm^2, α_t = 45°, **e** 40×40 μm^2, α_t = 45°, and **f** 60×40 μm^2, α_t = 45°. Scale bars, 10 μm. FEM simulation results of self-assembled immersive-etched parallelogram strip nanomembrane with sizes of **g** 4×40 μm^2, α_t = 45°, **h** 10×40 μm^2, α_t = 45°, **i** 20×40 μm^2, α_t = 45°, **j** 40×40 μm^2, α_t = 45°, and **k** 60×40 μm^2, α_t = 45°.

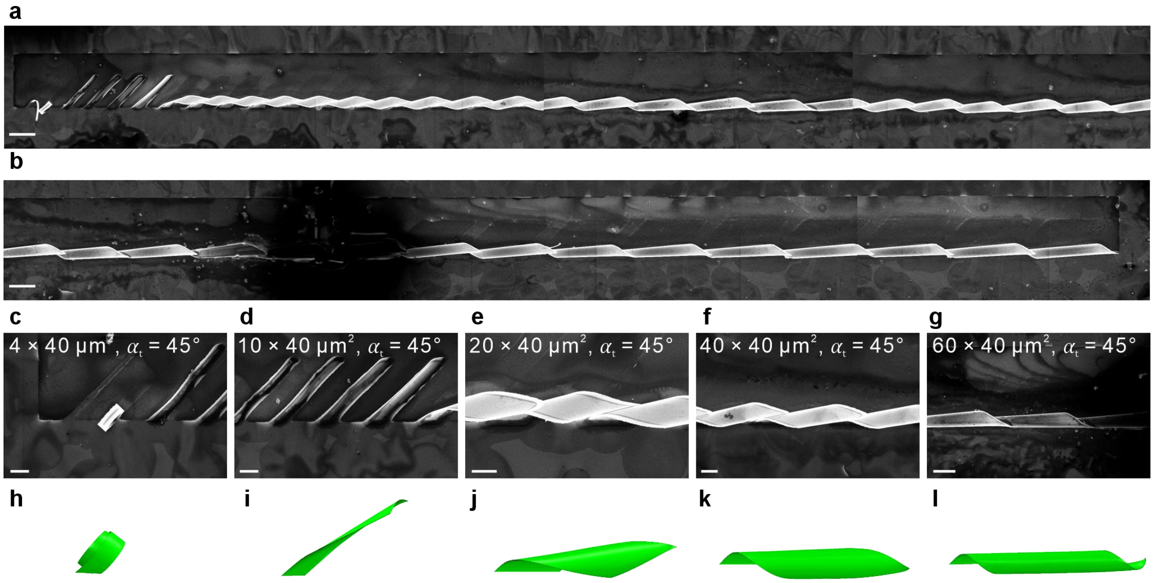

Supplementary Figure 55. SEM images and FEM results of bottom-etched parallelogram strips with different widths. a, b Panorama SEM images of bottom-etched self-assembled parallelogram strip nanomembranes with different widths. Scale bars, 30 μm. SEM images of self-assembled parallelogram strip immersive-etched nanomembrane with sizes of **c** 4×40 μm^2 , $\alpha_t = 45°$ (scale bar, 10 μm), **d** 10×40 μm^2, $\alpha_t = 45°$ (scale bar, 10 μm), **e** 20×40 μm^2, $\alpha_t = 45°$ (scale bar, 10 μm), **f** 40×40 μm^2, $\alpha_t = 45°$ (scale bar, 10 μm), and **g** 60×40 μm^2, $\alpha_t = 45°$ (scale bar, 20 μm). FEM simulation results of self-assembled immersive-etched parallelogram strip nanomembrane with sizes of **h** 4×40 μm^2, $\alpha_t = 45°$, **i** 10×40 μm^2, $\alpha_t = 45°$, **j** 20×40 μm^2, $\alpha_t = 45°$, **k** 40×40 μm^2, $\alpha_t = 45°$, and **l** 60×40 μm^2, $\alpha_t = 45°$.

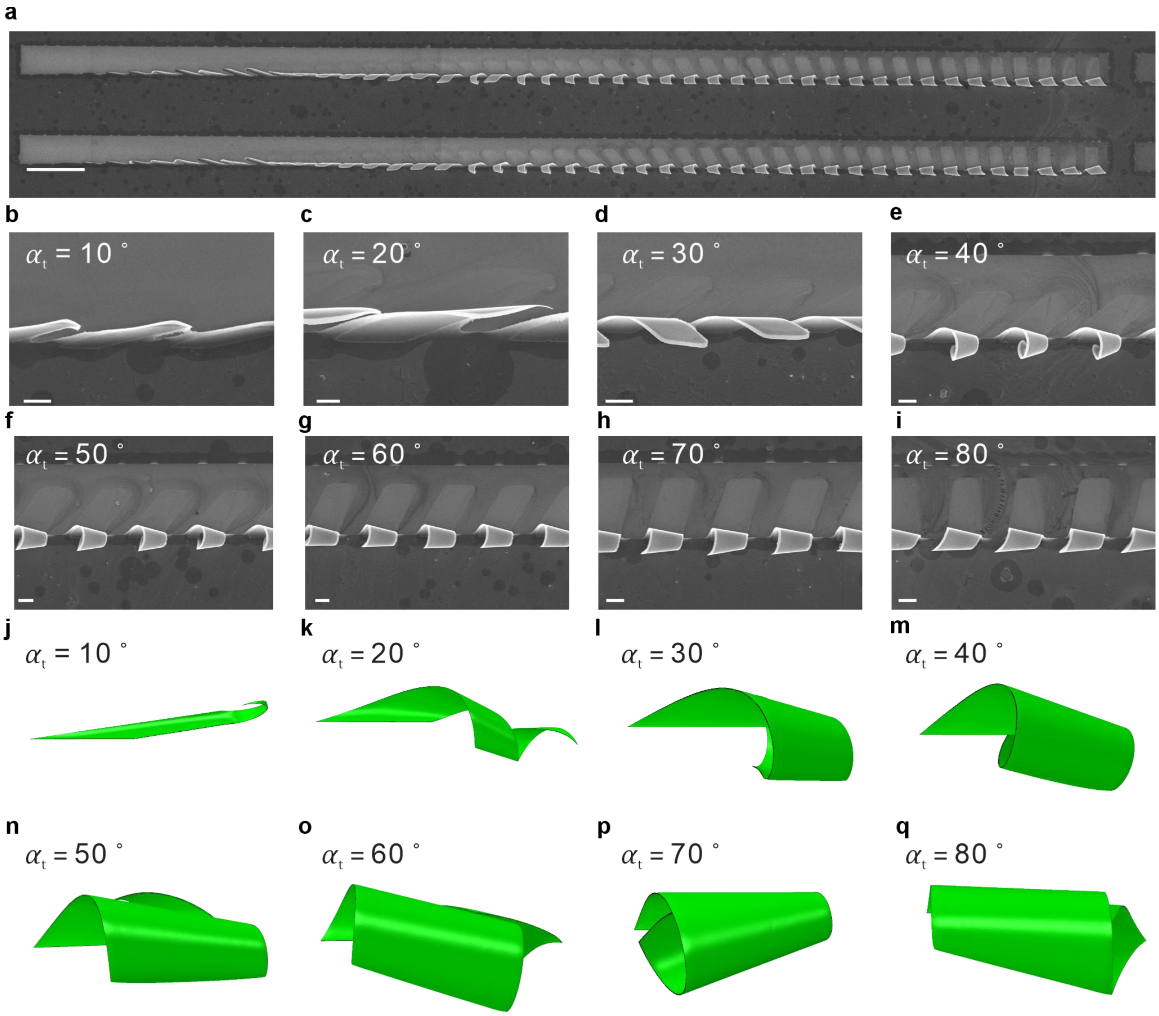

Supplementary Figure 56. SEM images and FEM results of the bottom-etched self-assembled parallelogram strips with different tilt angles. a Panorama SEM images of immersion etching of self-assembled parallelogram strips at different tilt angles. Scale

bars, 100 μm. SEM images of bottom-etched self-assembled parallelogram strips with angles of **b-i** α_t = 10°-80°. Scale bars, 10 μm. FEM results of bottom-etched self-assembled parallelogram strips with angles of **j-q** α_t = 10°-80°.

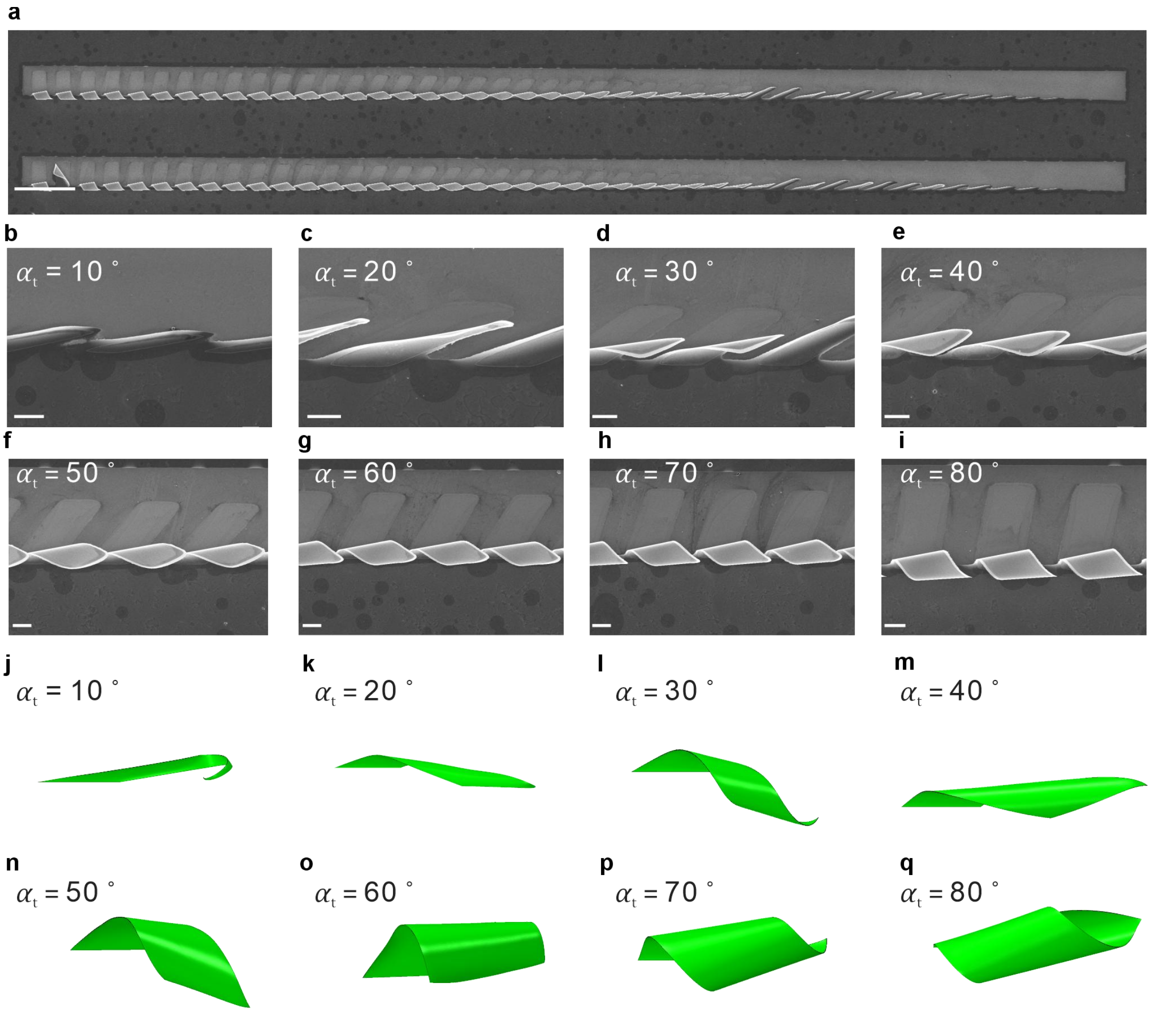

Supplementary Figure 57. SEM images and FEM results of top-etched self-assembled parallelogram strips with different tilt angles. a Panorama SEM images of immersion etching of self-assembled parallelogram strips at different tilt angles. Scale bars, 100 μm. SEM images of top-etched self-assembled parallelogram strips with angles of **b-i** α_t = 10°-80°. Scale bars, 10 μm. FEM results of top-etched self-assembled parallelogram strips with angles of **j-q** α_t = 10°-80°.

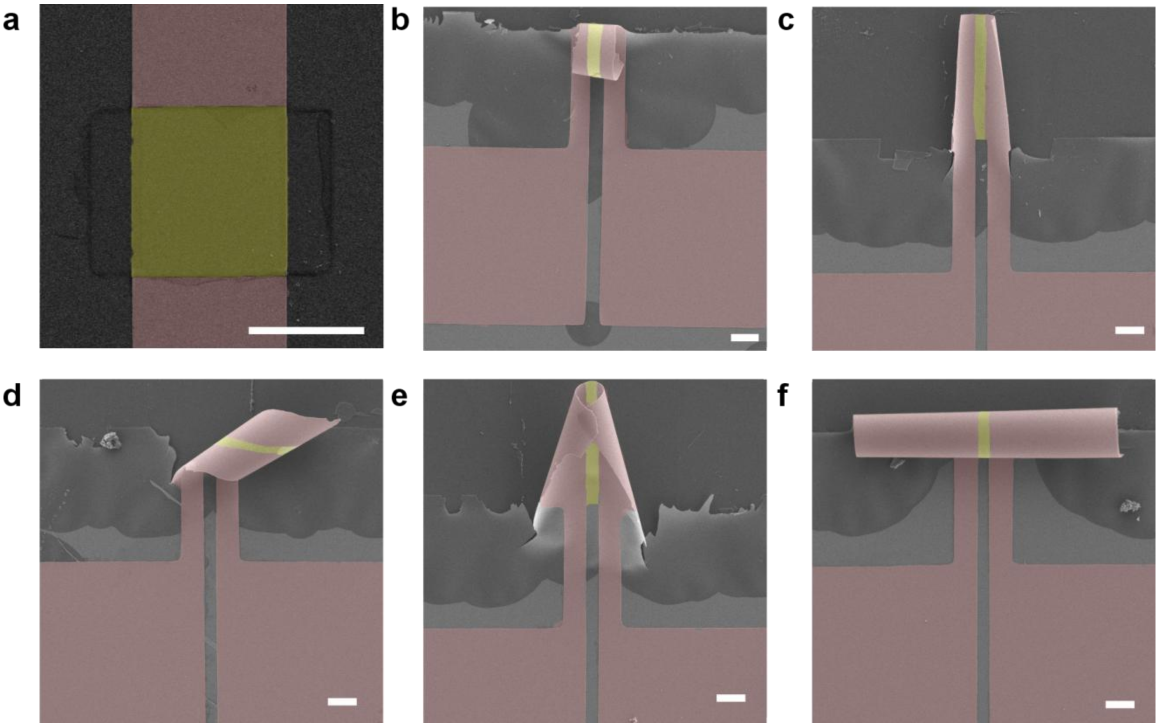

Supplementary Figure 58. SEM images for Si/Cr photodetectors in different structures. Photodetectors in **a** planar, **b** ring, **c** arch, **d** helix, **e** taper, and **f** tube structures. Scale bars, 10 μm.

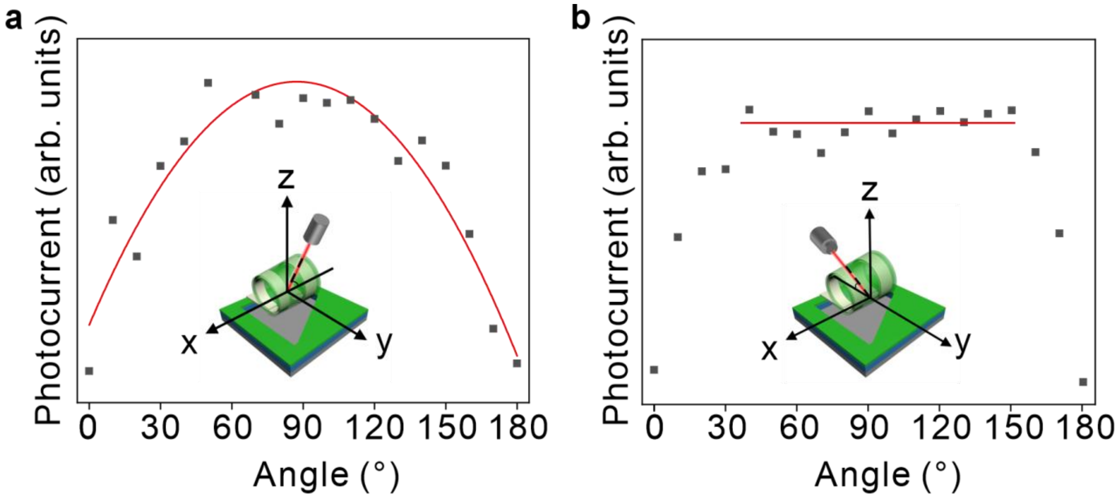

Supplementary Figure 59. Omnidirectional photocurrent of photodetectors. Angular photocurrent of tubular photodetector illuminated from **a** x axis and **b** y axis.

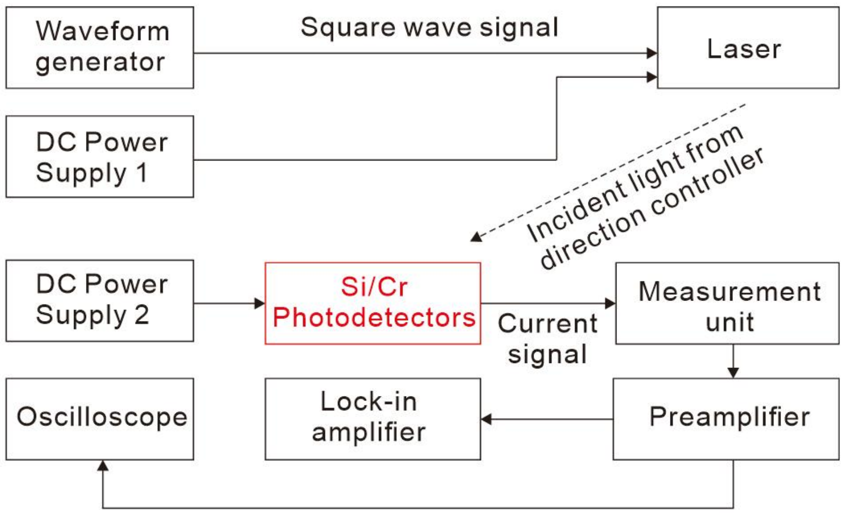

Supplementary Figure 60. Schematic diagram of 3D structure Si/Cr photodetector testing equipment.

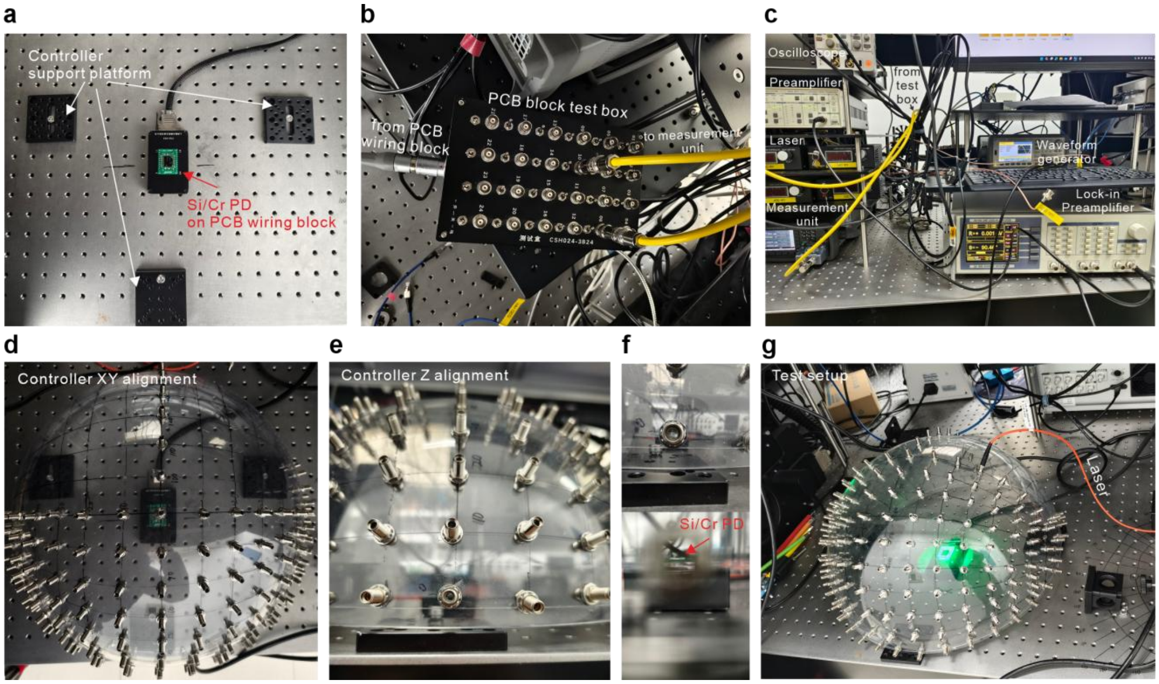

Supplementary Figure 61. Testing Setup for 3D structured photodetector. a Positioning of device and controller support platform. **b** Connection of devices, test boxes, and test units. **c** Test unit connection and schematic diagram. The **d** XY plane of the incident light direction controller is aligned with the **e-f** Z axis. **g** Schematic diagram of photodetection testing process.

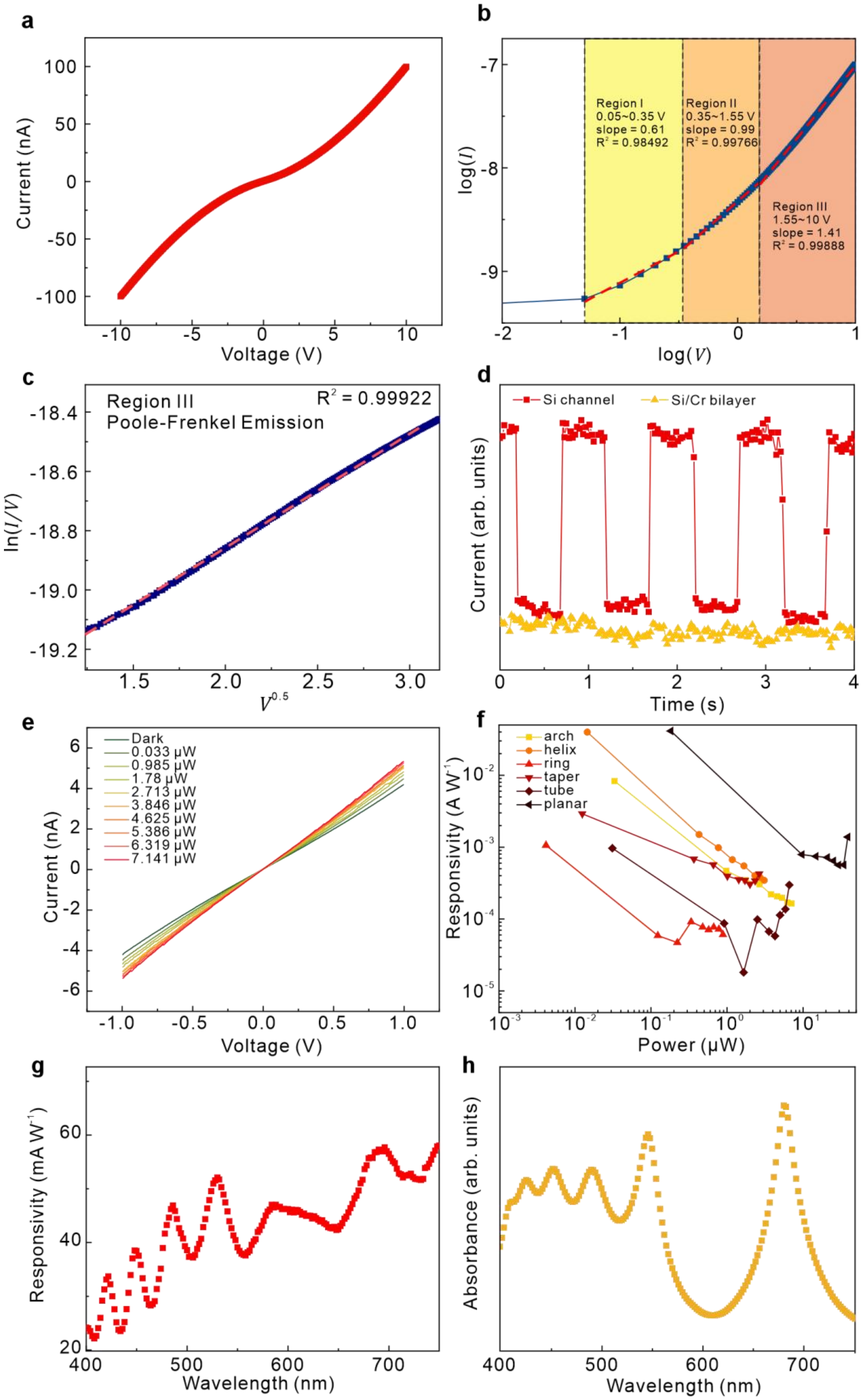

Supplementary Figure 62. Electrical properties and photoelectric responsivity of Si/Cr photodetector. **a** *I*-*V* curve and **b** log(*I*)-log(*V*) curve of arch-type photodetector. **c**

The $\ln(I/V)$- $\sqrt{V}$ relationship curve of arch-type photodetector in the poole-frenkel emission region. **d** Photoresponse of *channel* area and metal-semiconductor junction area of arch-type photodetector. **e** *I*-*V* curves of arch-type photodetectors under 520 nm laser irradiation with different powers. **f** Responsivity-power relationship diagram of photodetectors with different structures. **g** Responsivity-wavelength relationship of arch-type photodetector. **h** Simulated absorbance of Ge/Si/Cr in planar SiO_2 (35 nm)/Ge (50 nm)/Si (60 nm)/Cr (40 nm) multilayer.

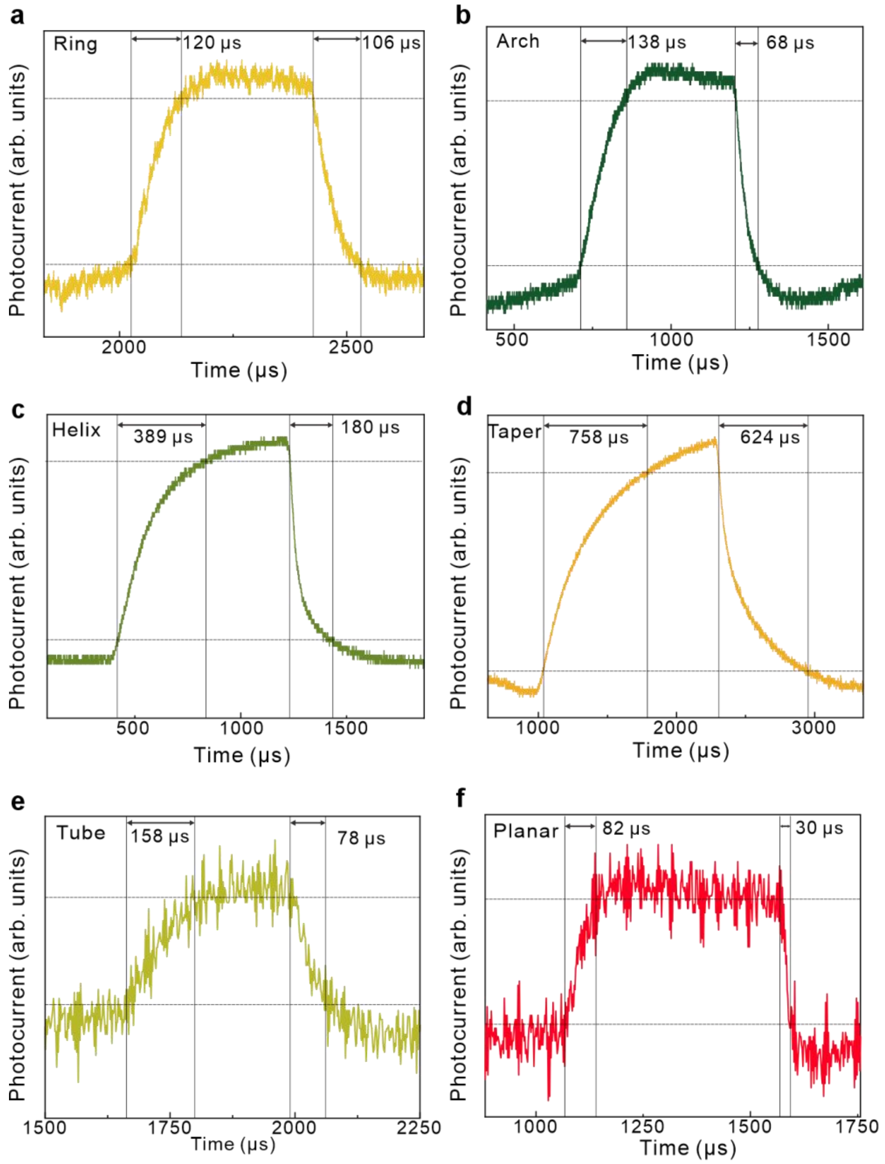

Supplementary Figure 63. Response time of different photodetectors. Response time of **a** ring, **b** arch, **c** helix, **d** taper, **e** tube, and **f** planar Si/Cr photodetectors.

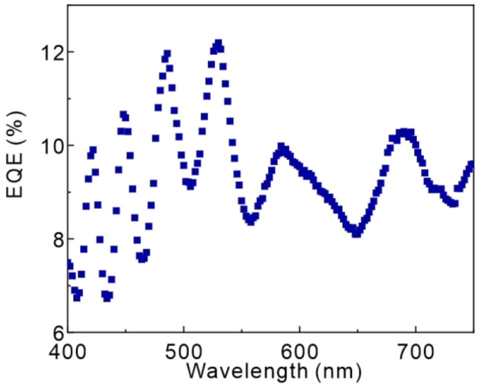

Supplementary Figure 64. EQE-wavelength relationship of the arch-type photodetector.

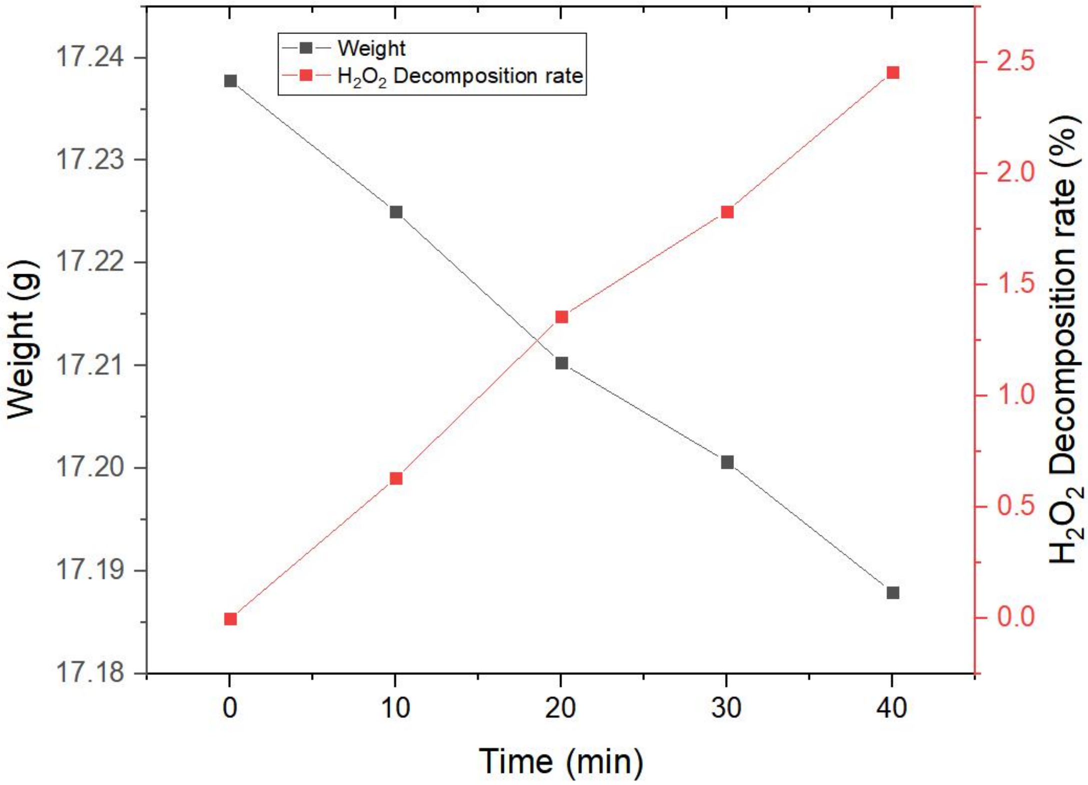

Supplementary Figure 65. Weight and H_2O_2 decomposition rate under 75 ℃ of heating.

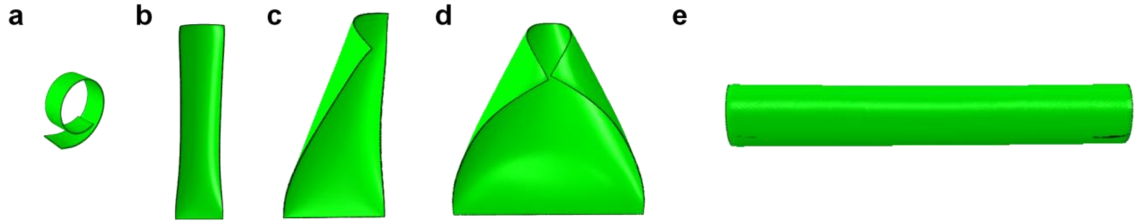

Supplementary Figure 66. FEM results of steady reaction-diffusion model. FEM simulation results of **a** ring, **b** arch, **c** helix, **d** taper, and **e** tube.

1503

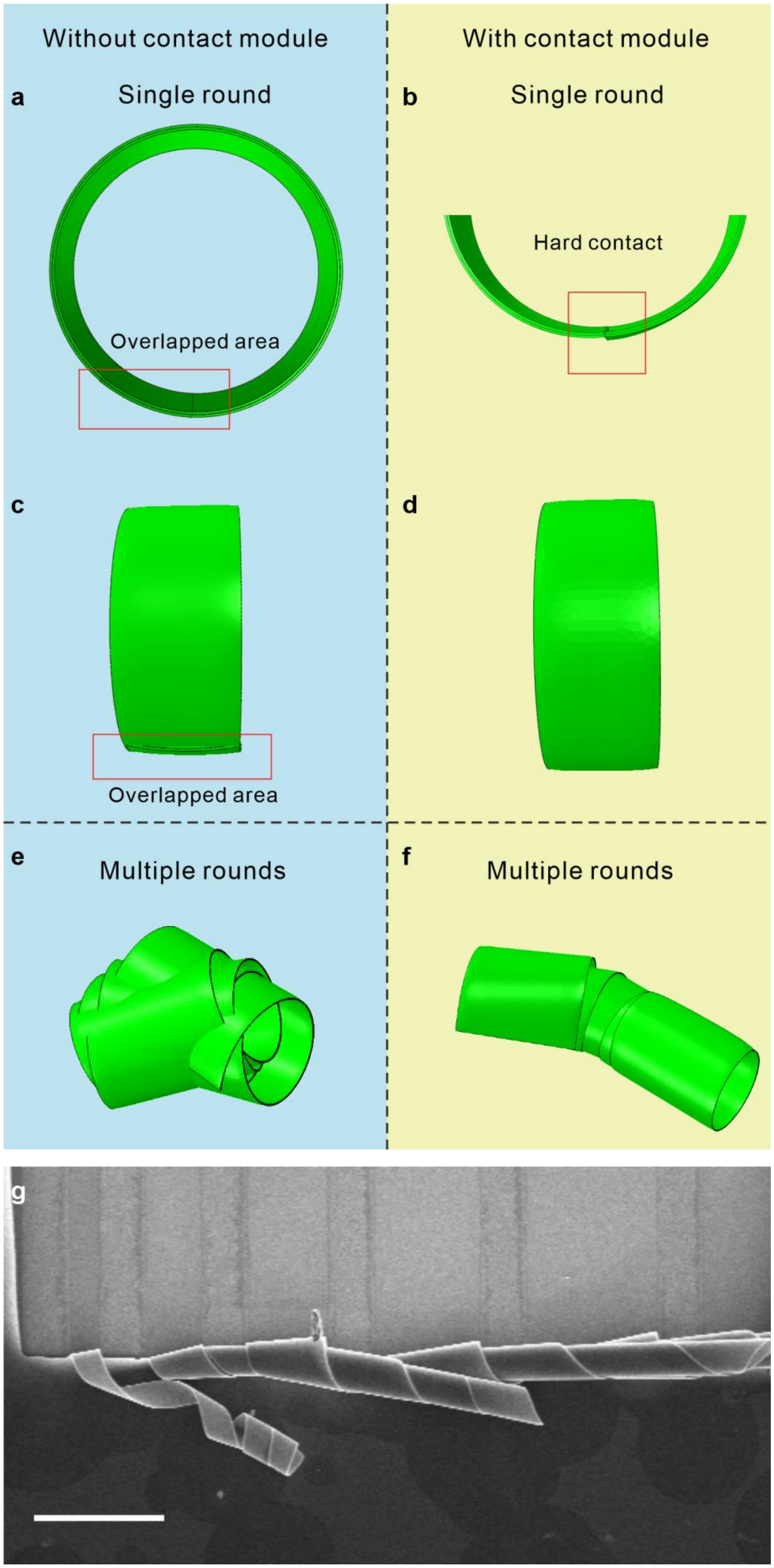

Without contact module
With contact module
a
Single round
Overlapped area
b
Single round
Hard contact
c
Overlapped area
d
e
Multiple rounds
f
Multiple rounds
g

Supplementary Figure 67. Schematic diagram of contact module in the multilevel design model. a, **c** FEM simulation results of the multilevel model in the case of single-turn rolling without the contact module. **b**, **d** FEM simulation results of the multilevel model in the case of single-turn rolling when applying the contact module. **e** FEM simulation results of the multilevel model in the case of multi-turn rolling without applying the contact module. **f** FEM simulation results of the multilevel model in the case of multi-turn rolling when applying the contact module. **g** SEM images of multi-turn rolling Si/Cr nanomembrane. Scale bars, 50 μm.

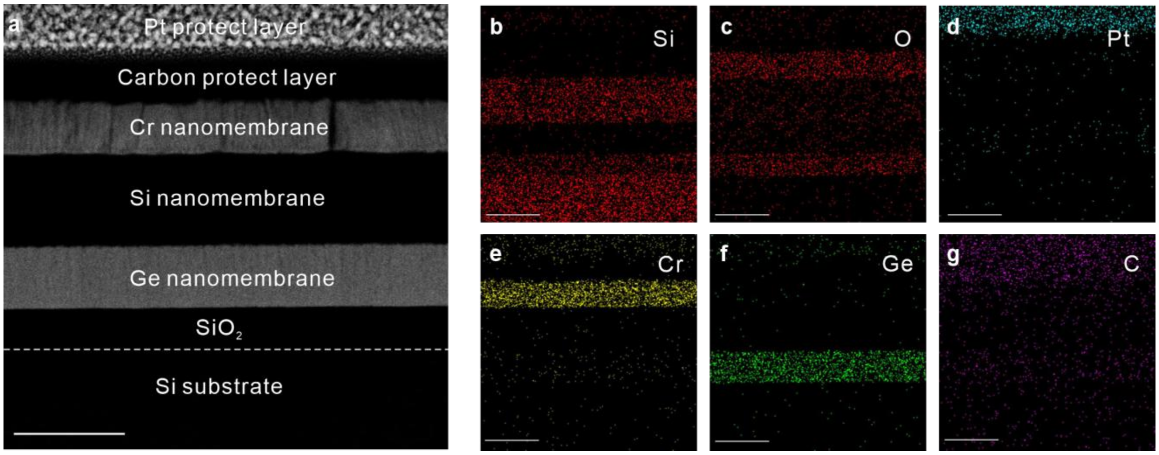

Supplementary Figure 68. Characterization of the cross-sectional structure and element distribution of the nanomembrane sample. a HAADF-STEM image of the cross-section of the sample. EDX images of the distribution of **b** Si, **c** O, **d** Pt, **e** Cr, **f** Ge, and **g** C elements in the cross section of the sample. Scale bars, 100 nm.

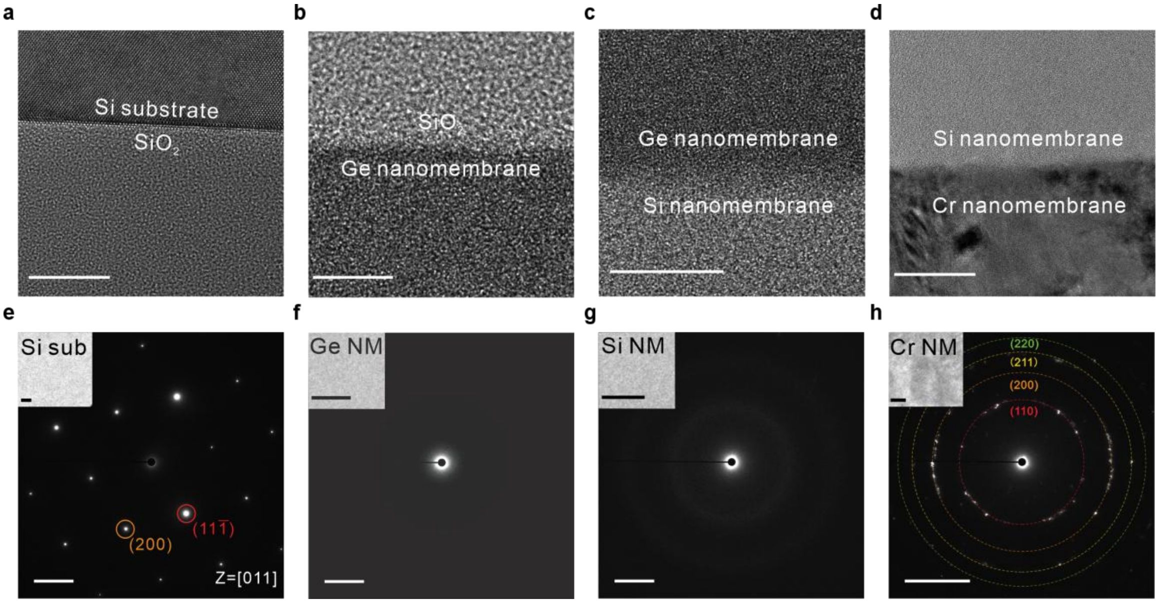

Supplementary Figure 69. Characterization of the interface morphology of the nanomembrane sample substrate and analysis of the crystal form of each layer of nanomembrane. HRTEM images of **a** silicon substrate/silicon oxide (scale bar, 10 nm), **b** silicon oxide/germanium nanomembrane (scale bar, 5 nm), **c** germanium nanomembrane/silicon nanomembrane (scale bar, 10 nm), **d** silicon nanomembrane/chromium nanomembrane interface (scale bar, 10 nm). SAED images of **e** silicon substrate, **f** germanium nanomembrane, **g** silicon nanomembrane, and **h** chromium nanomembrane (the inset is the TEM images of the corresponding SAED area). Scale bars of SAED in **e-h**, 5 nm^{-1}. Scale bars of TEM in **e-h**, 20 nm.

Supplementary Tables

Supplementary Table 1. Si/Cr nanomembrane sample numbers and corresponding parameters.

Sample number	Si thickness (nm)	Cr thickness (nm)
1	60	10
2	60	20
3	60	60
4	15	40
5	30	40
6	90	40
Standard	60	40

Supplementary Table 2. Comparison of photodetection performance with other photodetectors in this work.

Detector material	Detection mechanism	Detection range	Responsivity	Photoconversion efficiency	Response time/response frequency	Source
Si (60 nm)	Photoconductive	400-750 nm	52.5 mA W^{-1} @ 530 nm	12.2%@ 530 nm (EQE)	138μs (rise)/68μs(decay)	This work
Si(bulk)	Photovoltaic	350-1100 nm	640 mA W^{-1} @ 980 nm	82.2%@ 980 nm (EQE)	10 ns (rise)/10 ns (decay)	THORLABS FDS100-CAL
Si/Graphene	Photovoltaic	1300-1600 nm	30 mA W^{-1} @ 1550 nm	10%@1550 nm (IQE)	~18 GHz	Ref. 31
Si (bulk)	Photoconducti	400-700	~4 A W^{-1} @540 nm	~900%@ 540 nm	30 ms	JCHL GL48569

	ve	nm				photoresistor
Silicon (300 nm)	Photovoltaic	400-800 nm	80 mA W^{-1} @600 nm	-	9 GHz	Ref. 32
Si(bulk):Ag	Photoconductive	400-1200 nm	1.71 A W^{-1} @800 nm	266%@800 nm (EQE)	12.5 μs (rise)/15.9 μs (decay)	Ref. 33
CdTe nanoparticles	Photoconductive	365 nm, 500-800 nm	4 mA W^{-1} (visible wavelength)	-	134 μs	Ref. 34
β-Ga_2O_3	Photoconductive	200-300 nm	61.3 A W^{-1} @254 nm	30000% @254 nm	3 ms (rise)/35 ms (decay)	Ref. 35

Supplementary References

1. Roussel, M. R. (2019) Reaction–diffusion equations. in *Nonlinear Dynamics* (Morgan & Claypool Publishers), pp 13-11-13-16.
2. Lide, D. R. (2004) *CRC handbook of chemistry and physics* (CRC press).
3. White, F. M. (1979) *Fluid mechanics* (Tata McGraw-Hill Education).
4. Carter, R. E., Kinetic model for solid-state reactions. *J. Chem. Phys.* **34**, 2010-2015 (1961).
5. Chang, R., Goldsby, K. (2010) *General chemistry* (McGraw-Hill Education).
6. Shin, C. B., Economou, D. J., Effect of transport and reaction on the shape evolution of cavities during wet chemical etching. *J. Electrochem. Soc.* **136**, 1997-2004 (1989).
7. Noyes, A. A., Whitney, W. R., The rate of solution of solid substances in their own solutions. *J. Am. Chem. Soc.* **19**, 930-934 (1897).
8. Kosior, D., Wiertel-Pochopien, A., Kowalczuk, P. B., Zawala, J. Bubble Formation and Motion in Liquids: A Review. *Minerals.* 13(9). (2023). doi.org/10.3390/min13091130.
9. Samkhaniani, N., Ansari, M. R., Numerical simulation of superheated vapor bubble rising in stagnant liquid. *Heat Mass Transf.* **53**, 2885-2899 (2017).
10. Huang, W., et al., Three-dimensional radio-frequency transformers based on a self-rolled-up membrane platform. *Nat. Electron.* **1**, 305-313 (2018).
11. Sang, L., et al., Monolithic radio frequency SiN_x self-rolled-up nanomembrane interdigital capacitor modeling and fabrication. *Nanotechno.* **30**, 364001 (2019).
12. Kim, M.-S., Lee, H.-T., Ahn, S.-H., Laser Controlled 65 Micrometer Long Microrobot Made of Ni-Ti Shape Memory Alloy. *Adv. Mater. Techno.* **4**, 1900583 (2019).
13. Wu, B., et al., One-step rolling fabrication of VO_2 tubular bolometers with polarization-sensitive and omnidirectional detection. *Sci. Adv.* **9**, eadi7805 (2023).
14. Li, X., et al., Self-rolling of vanadium dioxide nanomembranes for enhanced multi-level solar modulation. *Nat. Commun.* **13**, 7819 (2022).

15. Huang, W., et al., Precision structural engineering of self-rolled-up 3D nanomembranes guided by transient quasi-static FEM modeling. *Nano Lett.* **14**, 6293-6297 (2014).
16. Xu, B., et al., Stimuli-responsive and on-chip nanomembrane micro-rolls for enhanced macroscopic visual hydrogen detection. *Sci. Adv.* **4**, eaap8203 (2018).
17. Landau, L. D., Lifshitz, E. M., Atkin, R., Fox, N. (2020) The Theory of Elasticity. *Physics of Continuous Media*, (CRC Press), pp 167-178.
18. Mei, Y., et al., Fabrication, Self-Assembly, and Properties of Ultrathin AlN/GaN Porous Crystalline Nanomembranes: Tubes, Spirals, and Curved Sheets. *ACS Nano* **3**, 1663-1668 (2009).
19. Nikishkov, G. P., Curvature estimation for multilayer hinged structures with initial strains. *J. Appl. Phys.* **94**, 5333-5336 (2003).
20. Hsueh, C.-H., Modeling of elastic deformation of multilayers due to residual stresses and external bending. *J. Appl. Phys.* **91**, 9652-9656 (2002).
21. Chun, I. S., et al., Geometry effect on the strain-induced self-rolling of semiconductor membranes. *Nano Lett.* **10**, 3927-3932 (2010).
22. Li, B., et al., Dependence of doubly curved regions on drying method in the fabrication of long-side rolled-up III-V microtubes. *Appl. Phys. Lett.* **103**, 051909 (2013).
23. Alben, S., Balakrisnan, B., Smela, E., Edge effects determine the direction of bilayer bending. *Nano Lett.* **11**, 2280-2285 (2011).
24. Cendula, P., et al., Bending and wrinkling as competing relaxation pathways for strained free-hanging films. *Phys. Rev. B* **79**, (2009).
25. Cendula, P., et al., Directional roll-up of nanomembranes mediated by wrinkling. *Nano Lett.* **11**, 236-240 (2011).
26. Armon, S., Efrati, E., Kupferman, R., Sharon, E., Geometry and Mechanics in the Opening of Chiral Seed Pods. *Science* **333**, 1726-1730 (2011).
27. Guo, Q., Zheng, H., Chen, W., Chen, Z., Modeling Bistable behaviors in Morphing Structures through Finite Element Simulations. *Biomed. Mater. Eng.* **24**, 557-562 (2014).

28. Seffen, K. A., Guest, S. D., Prestressed Morphing Bistable and Neutrally Stable Shells. *J. Appl. Mech.* **78**, 011002 (2010).

29. Zhou, G., et al., Coexistence of Negative Differential Resistance and Resistive Switching Memory at Room Temperature in TiOx Modulated by Moisture. *Adv. Electron. Mater.* **4**, 1700567 (2018).

30. Sze, S. M., Li, Y., Ng, K. K. (2021) *Physics of semiconductor devices* (John wiley & sons).

31. Pospischil, A., et al., CMOS-compatible graphene photodetector covering all optical communication bands. *Nat. Photon.* **7**, 892-896 (2013).

32. Xu, H., et al., High-Performance Lateral Avalanche Photodiode Based on Silicon-on-Insulator Structure. *IEEE Electron Device Lett.* **43**, 1077-1080 (2022).

33. Qiu, X., et al., Trap Assisted Bulk Silicon Photodetector with High Photoconductive Gain, Low Noise, and Fast Response by Ag Hyperdoping. *Adv. Opt. Mater.* **6**, 1700638 (2018).

34. Naje, A. N., Muhammed, G. S., Murad, H. I., Improvement of CdTe nanoparticles photoconductive detector by adding metal nanoparticles. *J. Optics* (2023). doi.org/10.1007/s12596-023-01165-2

35. Shen, G., et al., Solar-blind UV communication based on sensitive β-Ga_2O_3 photoconductive detector array. *Appl. Phys. Lett.* **123**, 041103 (2023).

36. Rogalski, A. (2000) *Infrared detectors* (CRC press).

37. Yun, J., et al., Effect of deposition temperature on electron-beam evaporated polycrystalline silicon thin-film and crystallized by diode laser. *Appl. Phys. Lett.* **104**, 242102 (2014).

38. Michael, A., et al., Investigation of E-Beam Evaporated Silicon Film Properties for MEMS Applications. *J. Microelectromech. Syst.* **24**, 1951-1959 (2015).

39. Haynes, W. M. (2014) *CRC handbook of chemistry and physics* (CRC press).
