# Supplementary material for: Multilevel design and construction in nanomembrane rolling for three-dimensional angle-sensitive photodetection
Source: Nat Commun. 2024 Apr 9;15:3066. doi: 10.1038/s41467-024-47405-2 (PMC11004118; doi:10.1038/s41467-024-47405-2)
Supplement: Supplementary file 3 — Description of Additional Supplementary Files [file 41467_2024_47405_MOESM3_ESM.pdf]

Description of additional supplementary files

Legends for Supplementary Movie 1 to 7

Supplementary Movie 1. Etchant concentration and flux distribution during etching process. This movie demonstrates the concentration and flux distribution of etchant in different patterns during etching process.

Supplementary Movie 2. Flow velocity during etching process. This movie demonstrates the flow velocity of different patterns when a bubble rises up around the pattern.

Supplementary Movie 3. Etching process of pre-strained nanomembranes. This movie demonstrates the optical movie of etching process of Si/Cr nanomembrane with different sizes.

Supplementary Movie 4. Selective etching by controlling etchant level by tilting wafer. This movie exhibits an etching direction selection via different tilting directions.

Supplementary Movie 5. Structural forming of pre-strained nanomembrane in top etching. This movie demonstrates quasistatic releasing process of pre-strained nanomembrane from top etching, which finally morph into a taper structure.

Supplementary Movie 6. Structural forming of pre-strained nanomembrane in bottom etching. This movie demonstrates quasistatic releasing process of pre-strained nanomembrane from bottom etching. When the nanomembrane is released incompletely, two sides of nanomembrane buckle up to build an energy barrier, which hinder the formation of taper structure, and finally turn into tube structure.

Supplementary Movie 7. Reconfiguration of Si/Cr microstructure via surface tension. This movie exhibits the structure reconfiguration of assembled Si/Cr nanomembrane from ring structure to planar pattern, and then reassembled into ring from planar pattern.
